# NIR Driven Pd/Cerium Oxide Nano‐Heterojunction for Enhanced Salvaging Sepsis Induced Acute Liver Injury via Reprogramming Redox Homeostasis in Synergy with Inducing Autophagy

**DOI:** 10.1002/advs.202417252

**Published:** 2025-06-29

**Authors:** Tao Qin, Lian Qin, Yang Zhao, Yin Chen, Qianyue Liu, Xiaoguang Lin, Yongfeng Lan, Yaohui Huang, Yan Liu, Ke Zhang, Lifan Pan, Jiaxiao Li, Kunpeng Duan, Hao Liang, Mingjing Yin, Guiyang Fan, Lian Liu, Yu Deng, Lin Liao, Danke Su, Ming Gao, Junyu Lu

**Affiliations:** ^1^ Department of Emergency Guangxi Medical University Cancer Hospital Nanning Guangxi 530201 China; ^2^ Intensive Care Unit The Second Affiliated Hospital of Guangxi Medical University Nanning Guangxi 530007 China; ^3^ Medical Imaging Center Guangxi Medical University Cancer Hospital Nanning Guangxi 530021 China; ^4^ Department of Clinical Laboratory Key Laboratory of Clinical Laboratory Medicine of Guangxi Department of Education The First Affiliated Hospital of Guangxi Medical University Nanning Guangxi 530021 China; ^5^ Key Laboratory of Micro‐Nanoscale Bioanalysis and Drug Screening Education Department of Guangxi Zhuang Autonomous Region Nanning Guangxi 530021 China; ^6^ Life Sciences Institute Guangxi Medical University Nanning Guangxi 530021 China; ^7^ Department of Anesthesiology Guangxi Medical University Cancer Hospital Nanning Guangxi 530201 China

**Keywords:** autophagy induction, keap1/Nrf‐2/HO‐1 pathway activation, redox homeostasis reprogramming, ROS scavenging, sepsis induced acute liver injury

## Abstract

Sepsis induced acute liver injury (SALI), is a type of acute and severe disease that is generally characterized by producing significant amounts of reactive oxygen species (ROS) in liver tissue, and in response to excessive ROS, producing huge amounts of inflammatory factors by hepatocytes. Considering the crucial role of ROS in SALI, a Pd doped CeO_2_ (CP) nano‐heterojunction with enhanced ROS scavenging capacity is developed to act as a catalytic nanomedicine for the treatment of SALI. Combining with near infrared (NIR) irradiation, it exhibits excellent scavenging capacity of ROS including hydroxyl radical (•OH), superoxide anion (•O_2_^−^), as well as singlet oxygen (^1^O_2_) for CP. Significantly, it also demonstrates the excellent antioxidant and anti‐inflammatory activities for lipopolysaccharides (LPS) stimulated macrophages (RAW264.7), and cecum ligation and puncture (CLP) treated C57BL/6J mice via reducing intracellular ROS levels, decreasing inflammatory factors expression levels, as well as activating Keap1/Nrf‐2/HO‐1 pathway to reprogram redox homeostasis, induce cellular autophagy, reduce systemic inflammation and promote liver tissue repair, finally achieving the alleviation of SALI. It provides a promising therapeutic strategy of CP+NIR with high efficacy and biosafety for the management of SALI.

## Graphical abstract

In vivo SALI therapy is achieved by scavenging intracellular ROS levels, downregulating inflammatory factors expression levels, inducing macrophage M2 directional polarization, and activating Keap1/Nrf‐2/HO‐1 pathway to reprogram redox homeostasis, induce cellular autophagy, reduce systemic inflammation, and promote liver tissue repair.

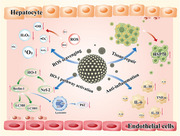

## 1 Introduction

Sepsis has been widely regarded as a life threatening multiple organs dysfunction caused by systemic inflammatory response to infection^[^ ^1^, ^2^ ^]^ When sepsis occurs, the liver is one of the main sites for inflammatory reactions and bacterial clearance, easily leading to acute liver injury (ALI).^[^ ^3^ ^]^ In the meantime, antibiotics therapy is an extremely important strategy of sepsis therapy. Nevertheless, with the extensive and inappropriate use of antibiotics, it can lead to multi‐drug resistant, and significantly increase the incidence of ALI.^[^ ^4^, ^5^ ^]^ Thus, related to adverse clinical outcomes, sepsis induced acute liver injury (SALI) has become one of severe public health issues in the world. At present, there is lack of effective strategies to prevent and treat SALI in clinical practice. Therefore, developing novel efficient therapeutic strategies is of great urgency.

SALI is mainly characterized by hepatocellular insufficiency. During hepatocyte metabolism, excessive generation of metabolite derived by antibiotics can deplete intracellular glutathione (GSH), subsequently leading to the formation of excessive reactive oxygen species (ROS) inside mitochondria.^[^ ^6^, ^7^ ^]^ And mitochondrial dysfunction ultimately caused hepatocyte necrosis. Apart from direct damage to hepatocytes, excessive ROS can stimulate them to secrete inflammatory factors like interleukin‐6 (IL‐6), tumor necrosis factor‐α (TNF‐α) and interleukin‐1 beta (IL‐1β), causing the activation of neutrophils and macrophages to induce inflammation.^[^ ^8^, ^9^ ^]^ The above conditions finally result in a vicious circle and intensify damage to hepatocytes. Thus, the therapeutic approach focused on ROS scavenging, further reprogramming redox homeostasis is expected to occupy a pivotal position in the prevention and treatment of SALI.^[^ ^10^, ^11^ ^]^ On the other hand, as an important self‐protection mechanism, autophagy is an evolutionary conserved degradation system. It participates in the formation of autophagosomes, and integration with lysosomes to degrade matters, which can circulate into the cytoplasm for cellular supplementation.^[^ ^12^ ^]^ The initiation of autophagy is crucial for cell survival and function, as it can remove damaged organelles and abnormal proteins, and provide additional energy.^[^ ^13^ ^]^ Previous studies confirmed that autophagy was helpful to restrict sustained NOD‐like receptor family pyrin domain containing 3 (NLRP3) inflammasome activation, balance inflammatory response and cell survival, and clear damaged cells, for alleviating sestrin 2 (SESN2) upregulation induced by lipopolysaccharide (LPS), pneumonia induced by Staphylococcus aureus, and sepsis related renal dysfunction.^[^ ^14^, ^15^ ^]^ Luo, etc. demonstrated that acute respiratory distress syndrome (ARDS) patients with the low expression of runt‐related transcription factor 1 (RUNX1) exhibited a low clearance efficiency of damaged mitochondria. Inducing RUNX1 expression was helpful to promoting mitochondrial autophagy, and further alleviating liver inflammation.^[^ ^16^ ^]^ These studies collectively suggested that inducing autophagy would be clinically beneficial for the treatment of SALI. However, due to their low biostability and biosafety, autophagy inducers are not suitable for clinical application.

With the development of nanotechnology, nanozymes are a kind of nanoparticles (NPs) with one or several catalytic activities of superoxide dismutase (SOD), catalase (CAT) and glutathione peroxidase (GPX), and could be applied in various biomedical fields such as biosensing, medical diagnosis, and diseases therapy.^[^ ^17^, ^18^, ^19^ ^]^ Among them, as an essential trace element, cerium (Ce) element can participate in various physiological activities, and play a significant role in maintaining intracellular redox homeostasis.^[^ ^20^, ^21^ ^]^ Notable, CeO_2_ NPs have been employed as ROS scavengers to alleviate inflammation related diseases like acute kidney injury,^[^ ^22^ ^]^ ARDS,^[^ ^23^ ^]^ bone regeneration,^[^ ^24^ ^]^ diabetic wound healing,^[^ ^25^ ^]^ anti‐tumor therapy^[^ ^26^, ^27^ ^]^ etc., attributed by the valence state transition between Ce^4+^/Ce^3+^, and oxygen vacancy. Though showing great potentials, the relatively low catalytic performances of CeO_2_ hinder its practical applications as biomedicines. Consequently, regulating the enzymatic catalytic activities of CeO_2_ NPs is essential for their extensive application. Recent studies have shown that the final enzymatic activities are significantly enhanced for Ce‐based nanozymes by doping with other elements like Au,^[^ ^28^ ^]^ Cu,^[^ ^29^ ^]^ Mn,^[^ ^30^ ^]^ Pt,^[^ ^31^ ^]^ et al to generate rich defects. Significantly, assisted by photothermal effect,^[^ ^32^ ^]^ ultrasonic driven,^[^ ^33^ ^]^ radiation or UV stimuli,^[^ ^34^ ^]^ the general properties are boosted for the engineered Ce‐based NPs, presenting the optimal catalytic functions with a low dosage. Meanwhile, Pd NPs have gained a great deal of interests for their good biocompatibility, colloidal stability, and multiple enzyme activities.^[^ ^35^ ^]^ Generally, NPs have remarkable size effects, and the smaller they are, the better their overall behaviors.^[^ ^36^ ^]^ Thus, ultrasmall Pd doped NPs possess stronger enzyme catalytic capacity and near infrared (NIR) absorbance, contributed to enhanced diseases therapy efficacies.^[^ ^37^, ^38^ ^]^ However, to our knowledge, there is currently no report on developing a novel Pd/CeO_2_ nano‐heterojunction (CP) as a NIR stimulated nanozyme to improve ROS scavenging efficiency for SALI therapy. Whether CP can induce cellular autophagy, and be used to treat inflammation related diseases also have not been investigated.

**Figure 1:**
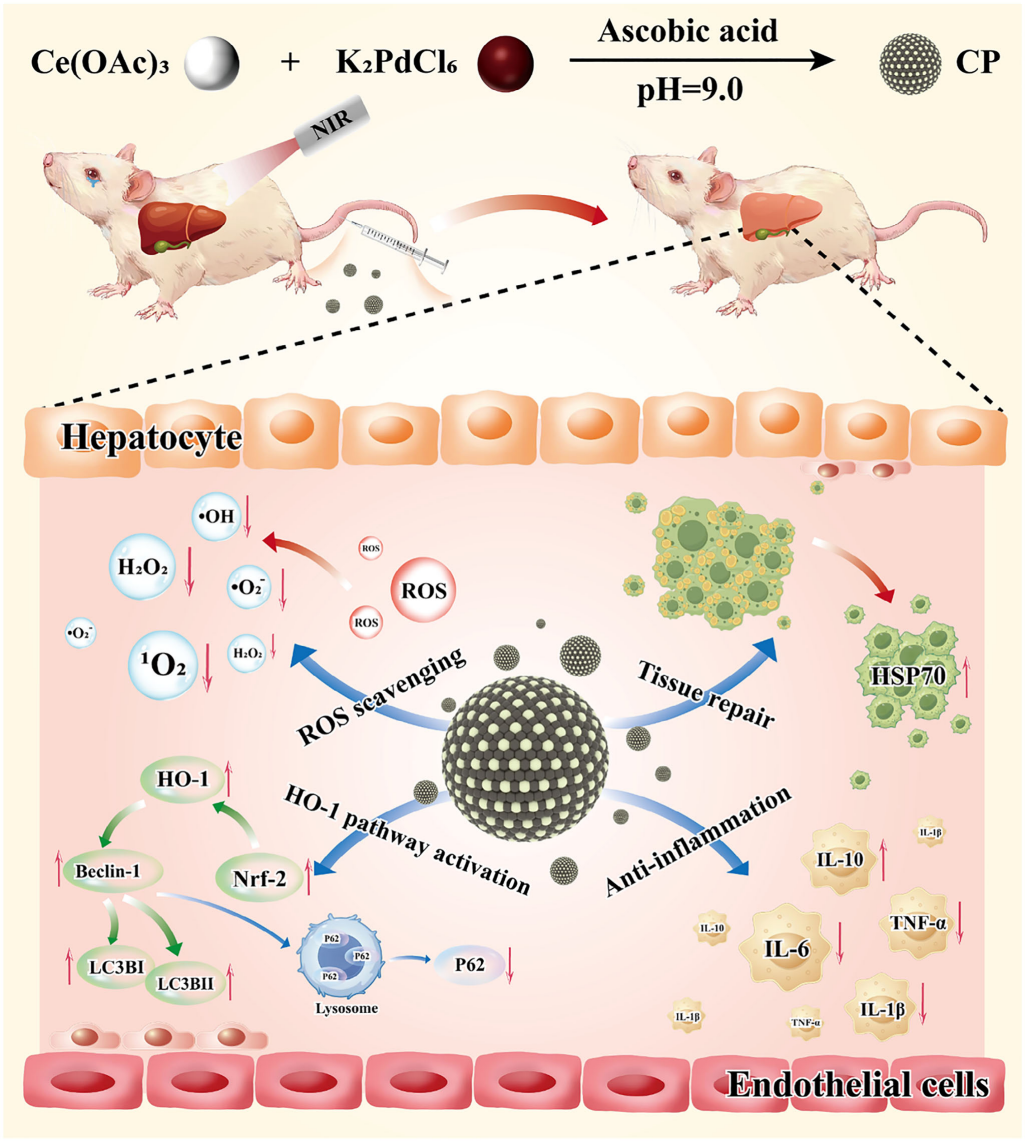
Schematic illustration of the preparation of CP, and the corresponding in vivo therapy of SALI mice model via scavenging intracellular ROS levels, downregulating inflammatory factors expression levels, inducing macrophage M2 polarization, and activating Keap1/Nrf‐2/HO‐1 pathway to reprogram redox homeostasis, induce cellular autophagy, reduce systemic inflammation, and promote liver tissue repair.

Herein, we reported a one‐pot synthetic method of the biocompatible CP. CP could scavenge multiple ROS, thereby efficiently reducing lipid peroxidation, and inhibiting cell death. In terms of mechanism, after being uptake by macrophages, CP mainly resided in cytoplasm, thereby clearing intracellular ROS, downregulating inflammatory factors, and activating kelch‐like epichlorohydrin‐associated protein 1/nuclear factor‐E2‐related factor 2/heme oxygenase‐1 (Keap1/Nrf‐2/HO‐1) pathway. Furthermore, it exhibited good biosafety, and was mainly enriched in the liver by intravenous (IV) injection of CP. Most significantly, combined with NIR irradiation, CP more efficiently reprogrammed liver redox homeostasis, induced hepatocellular autophagy, and accelerated liver tissue repair, achieving the enhanced alleviation of SALI (**Figure** **1**). To sum up, the good biocompatibility, outstanding ROS scavenging, and robust autophagy activation by the strategy of CP+NIR made it as a promising candidate for the prevention and therapy of ROS derived diseases.

## 2 Results and Discussion

### 2.1 Preparation and Physicochemical Characterization

**Figure 2:**
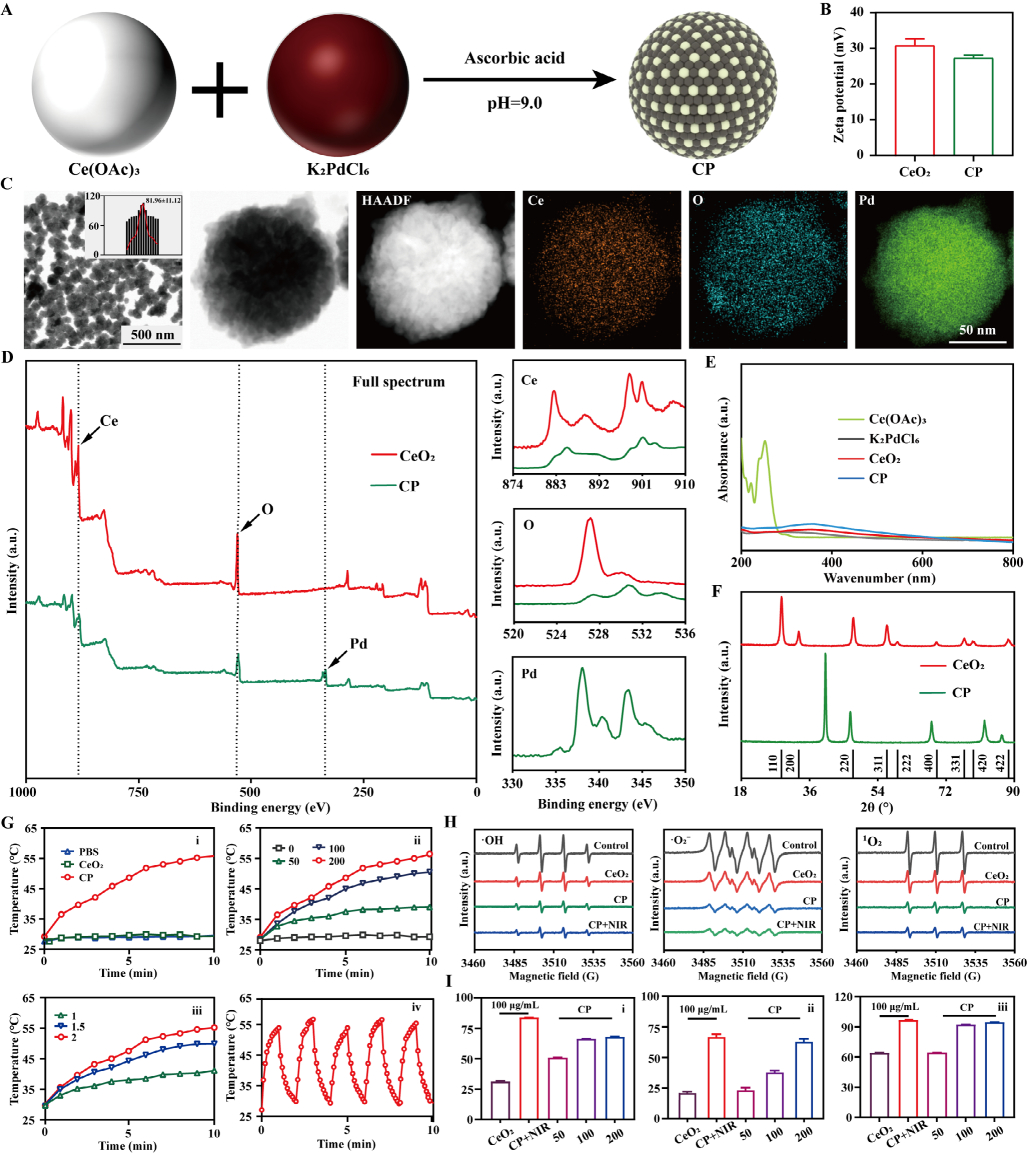
Preparation and physicochemical characterization of CP. A) Synthesis procedure of CP. B) Zeta potential of CeO_2_ and CP. C) TEM‐mapping images of CP, and the corresponding element composition (HAADF, Ce, O and Pd images). D) XPS results of CeO_2_ and CP (Full spectrum, and Ce, O and Pd detailed spectrum) E) UV–vis results of Ce(OAc)_3_, K_2_PdCl_6_, CeO_2_ and CP. F) XRD results of CeO_2_ and CP. G) Photothermal effects of PBS, CeO_2_ and CP with the same concentration of 200 µg mL^−1^ under NIR irradiation (2 W cm^−2^) (i), different concentration (0, 50, 100, and 200 µg mL^−1^) of CP under NIR irradiation (2 W cm^−2^) (ii), 200 µg mL^−1^ CP under different NIR irradiation intensity (1, 1.5, and 2 W cm^−2^) (iii) versus time, and the photothermal stability of 200 µg mL^−1^ CP under NIR irradiation (2 W cm^−2^) for 5 “on” and “off” cycles (iv). H) ROS scavenging ability of CeO_2_ and CP with the same concentration of 200 µg mL^−1^ by ESR: ·OH, ·O_2_^−^ and ^1^O_2_. I) ROS scavenging capacity by H_2_O_2_ (i), ·OH (ii) and ·O_2_^−^ (iii) testing kits: CeO_2_ and CP with the same concentration of 200 µg mL^−1^, and CP with 50, 100, and 200 µg mL^−1^.

CP was fabricated by the reduction reaction of Ce(OAc)_3_ and K_2_PdCl_6_ with the molar ratio of 1:1. In brief, Ce(OAc)_3_ and K_2_PdCl_6_ were dissolved in deionized (DI) water before the reduction of ascorbic acid. After adjusting the pH to weak alkaline environment (pH = 9.0), the solution reacted for 24 h (**Figure** **2**A). And then the solution was centrifuged at 12 000 rpm for 15 min, and re‐dispersed with DI water. It was repeated for three times before freeze drying to obtain CP. Specifically, for preparing CeO_2_, no K_2_PdCl_6_ was added into the reaction. Compared to CeO_2_ (faint yellow), it became dark for CP (Figure S1, Supporting Information) after the reduction reaction of Ce(OAc)_3_ (white) and K_2_PdCl_6_ (red). From Figure 2B, no obvious changes of zeta potential happened between CeO_2_ and CP with the zeta potential of 31.00 ± 2.17 and 27.27 ± 0.83 mV, respectively. From transmission electron microscopy combined with energy dispersive spectroscopy (TEM‐mapping) images, CP was in spherical shape with the diameter of 81.96 ± 11.12 nm (Figure 2C). And the obvious Ce, O, and Pd elements were observed for CP by mapping. Meanwhile, from X‐ray photoelectron spectroscopy (XPS), obvious Ce, O, and Pd elements were observed for CP while only Ce and O happened for CeO_2_ (Figure 2D). The extra C element was shown for CeO_2_ and CP possibly due to the existence of carbon film during preparation. From inductively coupled plasma mass spectrometer (ICP‐MS), the Ce and Pd elements were (8.46 ± 0.91)% and (8.02 ± 0.74)%, respectively for CP (Table S1, Supporting Information). Besides, the surface topography of CP was also investigated by atomic force microscope (AFM). It was around tens nm height for CP (Figure S2, Supporting Information). As illustrated in Figure 2E, obvious ultraviolet (UV) curve was observed for Ce(OAc)_3_ while no curves existed for K_2_PdCl_6_, CeO_2_ and CP. Meanwhile, compared to CeO_2_ with an obvious peak at 464 cm^−1^, there was a significant peak existed at 1567 cm^−1^ for CP by Raman spectroscopy (Figure S3, Supporting Information). And the crystallization structure of CP was shown in Figure 2F. Compared to CeO_2_ with the standard peaks existed at 28.5°, only four specific peaks happened at 40.1°, 46.7°, 68.2°, and 88.2° for CP.

Besides, to investigate their dispersion and stability in different physiological environment, CeO_2_ and CP were immersed in different solutions, where Dulbecco’s modified eagle medium (DMEM) and fetal bovine serum (FBS) corresponding to normal cellular microenvironment, and 5 mM H_2_O_2_ was equal to the inflammatory environment of induced cells.^[^ ^39^, ^40^ ^]^ As imaged in Figure S4, Supporting Information, CeO_2_ was well dispersed in different solutions, and almost totally precipitated into the bottom at 6 h. However, CP was also well dispersed in all solutions, and started to be deposited on the bottom at 12 h for FBS and 5 mM H_2_O_2_, and at 24 h for PBS. Generally, the dispersion of CP was better than that of CeO_2_. In the meantime, in vitro photothermal effects were also investigated. For PBS and CeO_2_, there were almost no changes of temperature during NIR irradiation for 10 min. However, for CP with the concentration of 200 µg mL^−1^, its temperature gradually increased versus time during irradiation, and reached 55.8 °C after 10 min’ irradiation (Figure S5, Supporting Information and i of Figure 2G). And for CP, increasing its concentration also contributed to improve photothermal effect, which was 29.3, 39.0, 50.5, and 56.4 °C for 0, 50, 100, and 200 µg mL^−1^ CP at 10 min, respectively (Figure S6, Supporting Information and ii of Figure 2G). Additionally, increasing the irradiation intensity from 1 to 2 W cm^−2^ also improved the corresponding temperatures from 41.1 to 55.2 °C for CP with the same concentration of 200 µg mL^−1^ (Figure S7, Supporting Information and iii of Figure 2G). Significantly, it also displayed stable photothermal effect of CP, with reversible heating and cooling process (iv of Figure 2G).

Furthermore, ROS scavenging capacities were also investigated. As shown in Figure 2H, comparing with control group with strong intensity of ·OH, CeO_2_ slightly decreased its intensity. CP obviously decreased the intensity of ·OH, enhanced by CP+NIR. It presented the same tendency for the scavenging of ·O_2_^−^ and ^1^O_2_ with the order of intensity: control group < CeO_2_ < CP < CP+NIR. In addition, ROS testing kits were also utilized to test the scavenging ability of ROS. As illustrated in i of Figure 2I and Table S2, Supporting Information, the H_2_O_2_ scavenging ratio was (31.00 ± 0.77)%, (66.02 ± 0.38)%, and (83.78 ± 0.17)% for CeO_2_, CP and CP+NIR of with the concentration of 100 µg mL^−1^. If increasing the concentration of CP from 50 to 200 µg mL^−1^, the H_2_O_2_ scavenging ratio jumped from (50.61 ± 0.65)% to (67.71 ± 0.77)% (Table S3, Supporting Information). Similarly, CP+NIR presented the best scavenging ratio of ·OH ((67.66 ± 1.42)%) and ·O_2_^−^ ((96.50 ± 0.20)%), followed by CP ((38.75 ± 0.57)% and (92.10 ± 0.28)%) and CeO_2_ ((21.77 ± 0.43)% and (63.94 ± 0.53)) (Tables S2 and S3, Supporting Information). If increasing the concentration of CP, the corresponding ·OH and ·O_2_^−^ also increased (ii and iii of Figure 2I). Additionally, to confirm the photothermal effects on ROS scavenging, NIR irradiation alone was also considered. As illustrated in Figure S8, Supporting Information, for OH and O_2_^−^ scavenging ratio, it was (44.40 ± 0.90)% and (73.91 ± 0.05)% for CP, enhanced to (76.79 ± 1.15)% and (82.61 ± 3.01)% for CP+NIR, respectively. However, NIR irradiation alone only contributed to (3.21 ± 0.21)% and (8.67 ± 5.22)% for OH and ·O_2_^−^ scavenging ratio, possibly due to experimental errors.^[^ ^39^, ^40^ ^]^

From the above, it confirmed the successful preparation of CP, in spherical shape with the diameter of 81.96 ± 11.12 nm, and zeta potential of 27.27 ± 0.83 mV. Compared to CeO_2_, almost no changes in chemical and molecular structure happened for CP. And slight changes in zeta potential happened between CeO_2_ and CP. Generally, positively charged ultrasmall metals doped CeO_2_ did not prominently contribute the changes of zeta potential, consistent with the previous results.^[^ ^41^, ^42^ ^]^ However, obvious changes were observed in the element composition and crystallization structure of CP. Compared to CeO_2_, for CP, it displayed improved dispersion and stability, and photothermal effects due to Pd doping, reducing the surface energy. Significantly, the enhanced ROS scavenging capacities of CP+NIR was achieved, attributed by the heterojunction structure and Pd doping together with NIR enhanced charge movement.^[^ ^39^, ^40^ ^]^ Increasing the concentration of CP always was helpful to improving photothermal effect and ROS scavenging. Significantly, the photothermal effect of CP was enhanced by increasing the irradiation intensity, and its ROS scavenging capacities were also enhanced by NIR irradiation, speeding up the movement of metal atoms. NIR irradiation alone did not effectively contribute to ROS scavenging capacities.

### 2.2 Biological Function Evaluation in Cellular Level

**Figure 3:**
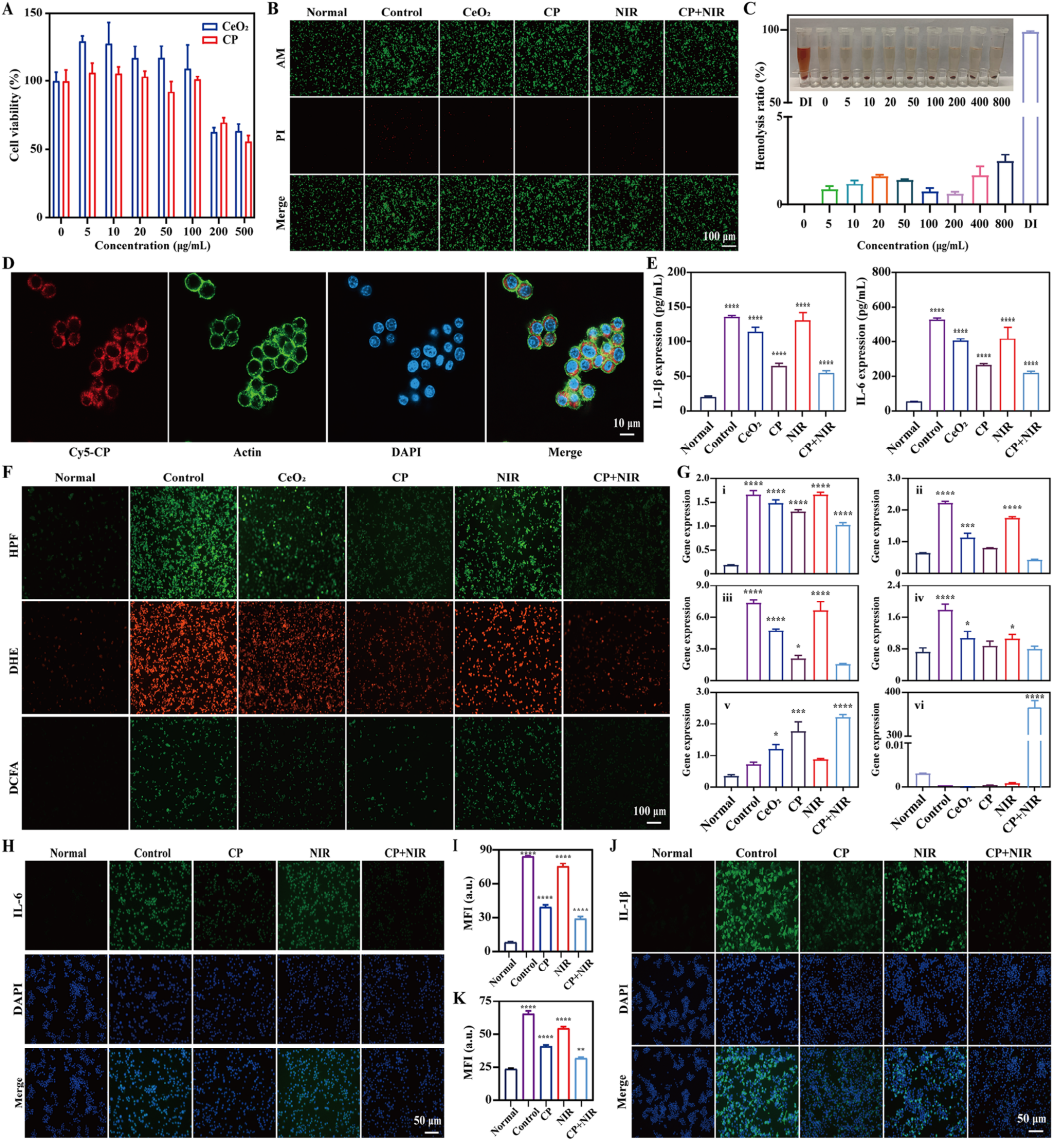
A) Cell viability of CeO_2_ and CP with the concentrations from 0 to 500 µg mL^−1^. B) Live/dead staining images of treated cells. C) Blood biocompatibility of CP with the concentrations from 0 to 800 µg mL^−1^. D) Cellular uptake images of cells incubated with Cy5‐CP for 3 h by confocal microscopy. E) Inflammatory factors (IL‐1β and IL‐6) expression levels of the supernatant of treated cells by ELISA. F) Intracellular ROS (HPF, DHE and DCFA) levels of treated cells by fluorescent microscope. G) Relative genes expression levels of treated cells by RT‐qPCR: TNF‐α (i), iNOS (ii), IL‐1β (iii), CD68 (iv), CD206 (v) and HSP70 (vi). H) IL‐6 expression level of treated cells by fluorescent microscope, and I) the corresponding quantified results. J) IL‐1β expression level of treated cells by fluorescent microscope, and K) the corresponding quantified results. The corresponding groups were: cells without treatment (normal group), LPS induced cells followed by PBS treatment (control group), LPS induced cells followed by 100 µg mL^−1^ CeO_2_ treatment (CeO_2_), LPS induced cells followed by 100 µg mL^−1^ CP treatment (CP), LPS induced cells followed by NIR irradiation (2 W cm^−2^) (NIR), and LPS induced cells followed by 100 µg mL^−1^ CP combining with NIR irradiation (2 W cm^−2^) (CP+NIR). (“*” symbol compared with normal group, *p < 0.05, **p < 0.01, ***p < 0.001 and ****p < 0.0001).

Macrophages are the major participants in the pathogenesis of inflammatory and immune diseases including sepsis.^[^ ^43^, ^44^ ^]^ For sepsis, M1 type macrophages can produce many inflammatory cytokines,^[^ ^45^ ^]^ including IL‐1β, TNF‐α and inducible nitric oxide synthase (iNOS) which are identified as important mediators and drivers of inflammation related diseases. In this part, mouse monocyte macrophage leukemia cells (RAW264.7) was selected as the research object. From **Figure** **3**A, it displayed the cell viability of CeO_2_ and CP, both of which were concentration depended. When the concentration was below 100 µg mL^−1^, it presented the outstanding biocompatibility for CeO_2_ and CP with the cell viability ≈100%. And it was significantly declined when the concentrations of CeO_2_ and CP were above 100 µg mL^−1^. Thus, 100 µg mL^−1^ was choosing as the concentration of CeO_2_ and CP for further experiments. Besides, live/dead staining was utilized to test the protection ability for LPS induced cells. As imaged in Figure 3B, compared to normal group with few dead cells (red fluorescence), many dead cells were shown for control group while it also displayed few dead cells for other groups. Specifically, almost no dead cells existed for NIR group, indicating that NIR irradiation did not kill cells. After statistical analysis, the live/dead ratio was 100.00 ± 2.77, 5.90 ± 1.82. 11.80 ± 1.99, 34.06 ± 1.41, 7.10 ± 1.80, and 63.71 ± 14.61% for normal group, control group, CeO_2_, CP, NIR and CP+NIR, respectively (Figure S9, Supporting Information). And the blood biocompatibility was evaluated by hemolysis testing. As shown in Figure 3C, compared to control group (DI water), the hemolysis ratio was below 5% for CP during the concentration ranging from 0 to 800 µg mL^−1^. It confirmed that CP presented good biocompatibility. Meanwhile, the cellular uptake ability was investigated by confocal microscopy. As imaged in Figure 3D, obvious red fluorescence (Cy5‐CP) was observed inside cells, indicated the strong cellular uptake capacities of CP.

The expression levels of inflammation related factors for treated cells were initially analyzed by enzyme linked immunosorbent assay (ELISA). As illustrated in i of Figure 3E, compared to normal group (18.84 ± 1.71), the IL‐1β expression level was highest for control group (134.80 ± 1.40) and NIR (130.10 ± 5.79). CeO_2_ slightly decreased its expression levels to 113.30 ± 6.49 while CP and CP+NIR effectively decreased the expression levels of IL‐1β to 64.16 ± 2.40 and 53.21 ± 3.48, respectively. It displayed the same tendency for IL‐6 expression levels with the order of control group (529.40 ± 6.07) > NIR (415.50 ± 33.97) > CeO_2_ (403.70 ± 3.46) > CP (264.30 ± 5.83) > CP+NIR (214.80 ± 6.53) > normal group (50.86 ± 3.86) (ii of Figure 3E). Next, the intracellular ROS levels of treated cells were also investigated by immunofluorescent staining and flow cytometry, respectively. As imaged in Figure 3F, obvious green fluorescence (hydroxyphenyl fluorescein (HPF) levels) existed in control group while it gradually became weak for CeO_2_, CP, CP+NIR and normal group, and slightly decreased for NIR. After statistical calculation, the mean fluorescence intensity (MFI) was 12.11 ± 4.13 for normal group, significantly increased to 144.70 ± 12.78 for control group and 136.70 ± 4.99 for NIR, and a certain degree of increase for CeO_2_ (95.07 ± 6.82), CP (56.62 ± 2.35), CP+NIR (35.20 ± 1.44) (Figure S10A, Supporting Information). It displayed the similar tendency for dihydroethidium (DHE, red fluorescence) and 2’, 7’‐dichlorodihydrofluorescein diacetate (DCFA, green fluorescence) levels, with the order of control group (210.50 ± 2.46 and 95.93 ± 2.21) > NIR (199.50 ± 14.06 and 81.46 ± 2.82) > CeO_2_ (117.00 ± 5.38 and 47.09 ± 8.03) > CP (90.62 ± 2.51 and 32.46 ± 6.04) > CP+NIR (62.42 ± 11.69 and 19.00 ± 0.67) > normal group (57.36 ± 7.43 and 4.91 ± 0.57) (Figure S10B,C, Supporting Information). In the meantime, by flow cytometry, the MFI was 610.70 ± 22.74, 2481.00 ± 405.10, 1194.00 ± 400.10, 1171.00 ± 345.20, 2531.00 ± 445.60, and 973.00 ± 231.70 for HPF levels in normal group, control group, CeO_2_, CP, NIR and CP+NIR, respectively (Figure S11A,B, Supporting Information). And for DHE and DCFA levels, the MFI was also in the order of control group (1605.00 ± 197.50 and 27 145.00 ± 13 751.00) > NIR (1541.00 ± 228.10 and 25 591.00 ± 9801.00) > CeO_2_ (811.30 ± 88.48 and 7793.00 ± 4435.00) > CP (766.70 ± 174.60 and 3211.00 ± 653.50) > CP+NIR (652.70 ± 103.50 and 797.00 ± 262.90) > normal group (578.30 ± 8.96 and 577.70 ± 30.66) (Figure S11C–F, Supporting Information). Next, the related genes expression levels were quantified analyzed by reverse transcription‐quantitative real‐time polymerase chain reaction (RT‐qPCR). For inflammatory factors: TNF‐α, iNOS and IL‐1β, their expression levels were lowest for normal group (0.19 ± 0.01, 0.66 ± 0.02 and 0.01 ± 0.01), significantly increased to (1.66 ± 1.18, 2.23 ± 0.10, and 7.36 ± 0.63) for control group, and slight increased to other groups including CeO_2_ (1.49 ± 0.13, 1.14 ± 0.26, and 4.75 ± 0.31), CP (1.31 ± 0.08, 0.81 ± 0.01, and 2.13 ± 0.48) and CP+NIR (1.02 ± 0.11, 0.43 ± 0.04, and 1.56 ± 0.12). No significant changes happened between control group and NIR (1.66 ± 0.12, 1.76 ± 0.08, and 6.68 ± 1.62). Meanwhile, for macrophage polarization, the CD68 expression was in low level for normal group (0.73 ± 0.10), gradually increased to 0.80 ± 0.08, 0.88 ± 0.12, 1.08 ± 0.17, 1.06 ± 0.10, and 1.79 ± 0.14 for CP+NIR, CP, CeO_2_, NIR and control group, respectively. Conversely, the order of CD206 expression levels was CP+NIR (2.21 ± 0.13) > CP (1.77 ± 0.52) > CeO_2_ (1.21 ± 0.24) > NIR (0.88 ± 0.04) > control group (0.73 ± 0.11) > normal group (0.35 ± 0.06). Significantly, the heat shock protein 70 (HSP70) expression was in highest level for CP+NIR (358.50 ± 40.36) while it was relatively low for other groups including normal group (0.01 ± 0.01), control group (0.01 ± 0.01), CeO_2_ (0.01 ± 0.01), CP 0.01 ± 0.01), and NIR (0.01 ± 0.01) (Figure 3G). Furthermore, immunofluorescent staining was also applied to characterize the inflammatory factors expression levels. As imaged in Figure 3H, compared to normal group with little green fluorescence, IL‐6 expression was in highest level for control group, followed by NIR, CP and CP+NIR. By analysis, the MFI was 7.84 ± 1.14 for normal group, increased to 84.42 ± 0.95, 75.47 ± 4.50, 39.60 ± 3.30, and 29.23 ± 2.99 for control group, NIR, CP and CP+NIR, respectively (Figure 3I). It presented the similar trend for IL‐1β expression with the order of control group (65.73 ± 3.63) > NIR (54.68 ± 1.99) > CP (41.05 ± 1.68) > CP+NIR (31.80 ± 1.66) > normal group (23.59 ± 1.17) (Figure 3J,K).

From the above, it displayed the good biocompability for CP within the concentration of 100 µg mL^−1^. And it confirmed that CP was easily uptake by cells to fulfill its intracellular functions, due to its nanosized diameter of ≈82 nm and positive charge of ≈27 mV. It also proved that CP could effectively downregulate the inflammatory factors expression levels, decrease intracellular ROS levels as well as induce macrophage M2 polarization. Combined with NIR irradiation, the foresaid effects were significantly enhanced. Significantly, CP+NIR also improved the HSP70 expression level, helpful to accelerating liver tissue repair.^[^ ^46^ ^]^

### 2.3 In Vivo SALI Therapy Evaluation

**Figure 4:**
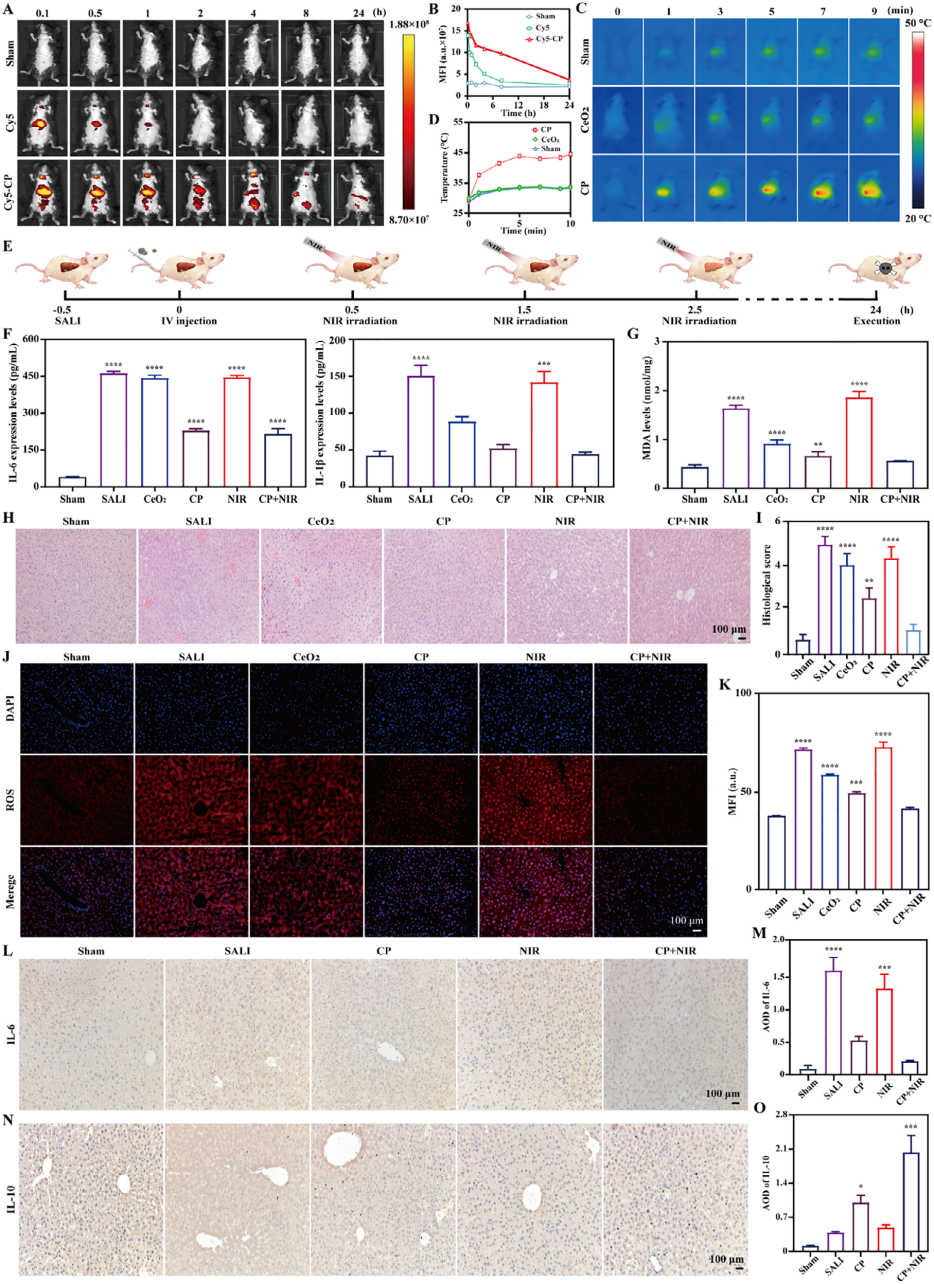
In vivo SALI therapy evaluation. A) In vivo bio‐distribution of CP at 0, 0.5, 1, 2, 4, 8, and 24 h by IVIS, and the corresponding quantified results (B). The corresponding groups were: mice injected with saline (sham group), Cy5 (Cy5), and Cy5‐CP (Cy5‐CP). C) In vivo photothermal images of treated mice versus time under NIR irradiation (2 W cm^−2^), and the corresponding quantified results (D). E) Time schedule of in vivo SALI therapy. F) Inflammatory factors (IL‐6 (i) and IL‐1β (ii)) expression levels of blood serum of treated mice by ELISA. G) MDA levels of liver homogenate of treated mice by the corresponding testing kits. H) H&E staining images of liver tissue of treated mice, and I) the corresponding histological score. J) ROS levels of liver tissue of treated mice, and K) the corresponding quantified results. L) IL‐6 expression levels of liver tissue of treated mice, and the corresponding quantified results (M). N) IL‐10 expression levels of liver tissue of treated mice, and the corresponding quantified results (O). The corresponding groups were: mice without treatment (sham group), LPS induced mice with saline injection (SALI), LPS induced mice with CeO_2_ injection (CeO_2_), LPS induced mice with CP injection (CP), LPS induced mice with NIR irradiation (2 W cm^−2^) (NIR), and LPS induced mice with CP injection and NIR irradiation (2 W cm^−2^) (CP+NIR). (“*” symbol compared with sham group, *p < 0.05, **p < 0.01, ***p < 0.001, and ****p < 0.0001).

For in vivo SALI therapy, in vivo biosafety was initially implemented after intravenous (IV) injection for 14 days. It displayed the similar tendency for the body weight of two groups, gradually increased versus time (Figure S12, Supporting Information). Similarly, no significant differences were observed for all blood indicators of sham group and CP (Figure S13, Supporting Information). And for hematoxylin and eosin (H&E) staining images of major organs, no apparent damages happened for CP, close to those of sham group (Figure S14, Supporting Information). Specifically, by in vivo animal imaging system (IVIS), no fluorescence was observed for sham group, while it could retain in lung and liver for a certain of time, and totally disappeared at 2 h for Cy5. However, for Cy5‐CP, obvious fluorescent intensity was observed in the lung, liver and kidney at the beginning. At 8 h, the fluorescence disappeared in liver, and a little of fluorescence happened in lung and kidney (**Figure** **4**A,B). Thus, it confirmed that the obvious in vivo biosafety existed for IV injection of CP, and CP could stay in liver for 8 h and was gradually cleared by the liver.

To further confirm the feasibility of in vivo photothermal therapy (PTT), photothermal camera was applied to monitor the temperature changes versus time after IV injection of CeO_2_ or CP for 0.5 h. Compared to sham group and CeO_2_, it clearly displayed that the temperature of liver gradually increased for CP (Figure 4C). After statistical calculation, the temperature started at 28.7, 29.9, and 29.3 °C for sham group, CeO_2_ and CP, respectively. After 10 min’ irradiation, the corresponding temperature separately became 33.3, 33.6, and 44.5 °C for sham group, CeO_2_ and CP (Figure 4D), indicating the feasibility of PTT for SALI.

SALI animal model was established by cecum ligation and puncture (CLP) of mice.^[^ ^47^, ^48^ ^]^ After 0.5 h, the corresponding therapy was implemented by IV injection. Specifically, for PTT, NIR irradiation was implemented for three times at 0.5, 1.5, and 2.5 h, respectively within 24 h after IV injection. The mice were sacrificed after treatment for 24 h, and the corresponding blood and organs were collected for further experiments (Figure 4E). As illustrated in Figure 4F, the inflammatory factors levels of blood serum were also investigated by ELISA. For IL‐6 and IL‐1β expression, they were in the lowest levels (39.08 ± 4.75 and 41.89 ± 11.44) for sham group, subsequently increased to 461.40 ± 15.30 and 150.10 ± 26.69 for SALI, 444.50 ± 15.91 and 141.40 ± 25.87 for NIR, 442.10 ± 21.34 and 88.36 ± 12.24 for CeO_2_, 228.50 ± 15.64 and 51.79 ± 9.74 for CP, and 214.40 ± 40.47 and 43.88 ± 5.60 for CP+NIR, respectively. Similarly, compared to sham group (0.43 ± 0.05), the malondialdehyde (MDA) was in the highest level for SALI (1.63 ± 0.04) and NIR (1.86 ± 0.07) while it was still in a high level for other groups with the order of CeO_2_ (0.91 ± 0.08) > CP (0.66 ± 0.05) > CP+NIR (0.56 ± 0.02) (Figure 4G). By H&E staining, the morphology of liver for sham group was intact with clear structures, and arranged in a cord like pattern, and no inflammatory infiltration was observed. For SALI, the structural arrangement was disordered with visible cell deformation and swelling. The increased number of inflammatory cells was observed in hepatic sinusoids accompanied by cell necrosis. And NIR did not alleviate the above situations, while obvious healing happened to other groups including CeO_2_, CP and CP+NIR. Among them, CP+NIR presented the best therapeutic effect, with the liver cell structure arranged neatly, reduced number of deformed cells, and significantly reduced infiltration of inflammatory cells (Figure 4H). After statistical analysis, the score was 0.70 ± 0.26, 5.33 ± 0.58, 4.33 ± 0.58, 2.67 ± 0.58, 4.67 ± 0.58, and 1.67 ± 0.29 for sham group, SALI, CeO_2_, CP, NIR and CP+NIR, respectively (Figure 4I). However, for other organs including heart, lung and kidney, no significant differences existed, indicating no damages to other organs during SALI therapy (Figure S15, Supporting Information). Meanwhile, the ROS levels of liver were also investigated. For SALI and NIR, their ROS (red fluorescence) were in high levels with the MFI of 72.00 ± 1.37 and 73.25 ± 4.48, while it significantly decreased for other groups with the MFI order of CeO_2_ (59.11 ± 0.55) > CP (49.78 ± 1.15) > CP+NIR (41.71 ± 2.39) > sham group (37.76 ± 0.66) (Figure 4K). In addition, the IL‐6 expression level of liver was also investigated. As shown in Figure 4L,M, it was in high levels for SALI with the average optical densities (AOD) of 1.60 ± 0.28, followed by NIR (1.31 ± 0.35), CP (0.55 ± 0.06), CP+NIR (0.23 ± 0.06) and sham group (0.07 ± 0.02). Conversely, for IL‐10 expression, it was in high levels for CP+NIR (2.03 ± 0.59), followed by CP (1.01 ± 0.27), NIR (0.50 ± 0.13), SALI (0.39 ± 0.03) and sham group (0.20 ± 0.01) (Figure 4N,O). Markedly, the HSP70 expression levels of livers were relative low (below 0.01) for sham group, SALI, CP and NIR, prominently enhanced for CP+NIR (1.28 ± 0.05) (Figure S16, Supporting Information).

In this part, it confirmed the favorable in vivo biosafety and feasibility of in vivo photothermal therapy by the strategy of SALI therapy. By IV injection, CP could be enriched in the liver for 4 h, and was gradually cleared by the liver. Significantly, it proved that the strategy of CP+NIR could most efficiently downregulate the IL‐6 and IL‐1β expression levels of blood serum, decrease the MDA and ROS levels of liver, downregulate the IL‐6 expression level of liver, and upregulate IL‐10 and HSP70 expression levels of liver, therapy alleviating SALI with high efficiency and biosafety.^[^ ^49^ ^]^

### 2.4 Therapeutic Mechanism Investigation

**Figure 5:**
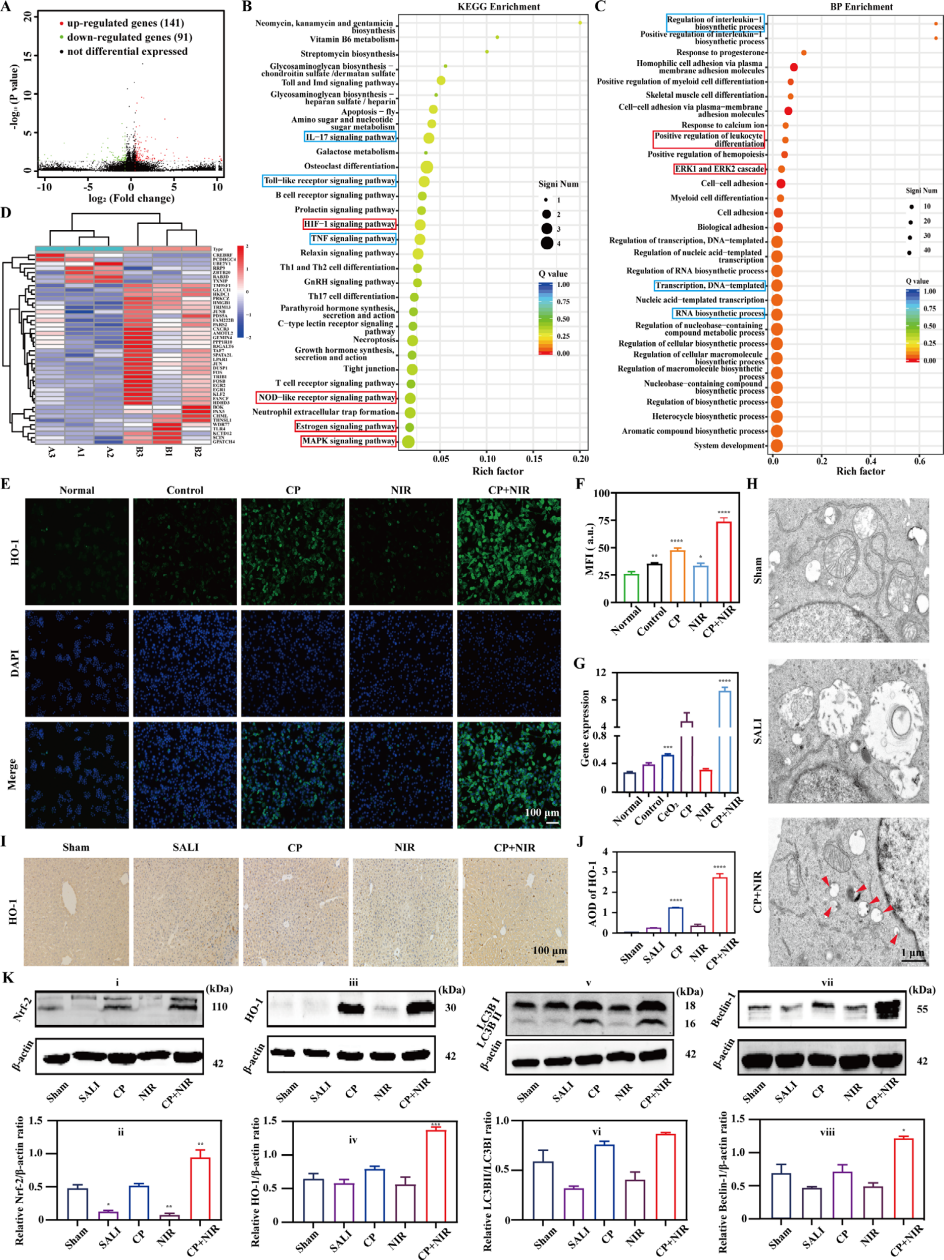
A) A volcano plotting of the landscape of DEGs between control group and CP+NIR. B) DEGs by KEGG enrichment analysis: autophagy related pathways (red box) and inflammation related pathways (blue box). C) DEGs by BP enrichment analysis: autophagy related pathways (red box) and inflammation related pathways (blue box). D) A heatmap of 44 autophagy related DEGs between control group and CP+NIR. E) HO‐1 expression levels of treated cells by fluorescent microscope and F) the corresponding quantified results. G) Relative HO‐1 gene expression levels of treated cells by RT‐qPCR. The corresponding groups were: cells without treatment (normal group), LPS induced cells followed by PBS treatment (control group), LPS induced cells followed by 100 µg mL^−1^ CP treatment (CP), LPS induced cells followed by NIR irradiation (2 W cm^−2^) (NIR), and LPS induced cells followed by 100 µg mL^−1^ CP combining with NIR irradiation (2 W cm^−2^) (CP+NIR). (“*” symbol compared with normal group, *p < 0.05, **p < 0.01, ***p < 0.001, and ****p < 0.0001) H) SEM images of liver tissue of treated mice. (autophagesome: red arrow) I) HO‐1 expression levels of liver tissue of treated mice, and J) the corresponding quantified results. K) The relative proteins expression levels of liver tissue of treated mice by WB: Nrf‐2 (i), HO‐1 (iii), LCB3I and LCB3II (v) and Beclin‐1 (vii), and the corresponding quantified results: relative Nrf‐2/β‐actin (ii), HO‐1/β‐actin (iv), LC3B/β‐actin (vi) and Beclin‐1/β‐actin (viii) ratios. The corresponding groups were: mice without treatment (sham group), LPS induced mice with saline injection (SALI), LPS induced mice with CP injection (CP), LPS induced mice with NIR irradiation (2 W cm^−2^) (NIR), and LPS induced mice with CP injection and NIR irradiation (2 W cm^−2^) (CP+NIR). (“*” symbol compared with sham group, *p < 0.05, **p < 0.01, ***p < 0.001 and ****p < 0.0001).

It had confirmed that IV injection of CP+NIR could efficiently alleviate SALI via reprogramming redox homeostasis both in vitro and in vivo. To investigate the therapeutic mechanism, the whole transcriptome sequence was implemented. By differential analysis, there were 232 differential expressed genes (DEGs) existed between control group and CP+NIR, where 142 upregulated and 91 downregulated genes (**Figure** **5**A). And it was found that some pathway functions had complex interactions with inflammation and autophagy, including mitogen activated protein kinase (MAPK), Estroge, nucleotide‐binding oligomerization domain (NOD) like receptor and hypoxia inducible factor‐1 (HIF‐1) signaling pathway which were associated with autophagy, and interleukin‐7 (IL‐7), Toll like receptor and TNF which were associated with inflammation by kyoto encyclopedia of genes and genomes (KEGG) (Figure 5B) and gene ontology (GO) (Figure 5C) enrichment analysis. In details, there were 4 genes enriched in the MAPK pathway. Previous studies had shown that MAPK signaling pathway could affect the formation and degradation of autophagosomes by adjusting the expression and activity of autophagy related proteins like Beclin‐1 and microtubule‐associated protein 1 light chain 3 (LC3).^[^ ^50^ ^]^ Activation of NOD containing 1 (NOD1) and NOD containing 2 (NOD2) receptor signaling pathway could induce autophagy by interacting with autophagy related proteins (autophagy related 5 (ATG5), autophagy related 7 (ATG7) and autophagy related 16 like 1 (ATG16L1)).^[^ ^51^ ^]^ And It also confirmed that Estrogen signaling pathway could affect autophagy activity by regulating mammalian target of rapamycin (mTOR) signaling pathway, and also affect the process of autophagy by regulating the expression of Beclin‐1 and LC3,^[^ ^52^ ^]^ where 2 genes were observed. Furthermore, 20 genes were enriched in extracellular regulated protein kinases (ERK1/2) cascade reaction, which could directly induce the phosphorylation of Beclin‐1, promote the formation of autophagosomes, and thus increase autophagy levels.^[^ ^53^ ^]^ Significantly, 3 genes were enriched in HIF‐1 signaling pathway which could upregulate the expression of BNIP3, and then bind to Rheb of mTOR to inhibit target of rapamycin complex 1 (TORC1), and promote autophagy. Similarly, there were 3, 3, 4, and 10 genes enriched in interleukin‐17 (IL‐17), Toll like receptor, TNF and interleukin‐1 (IL‐1) signaling pathway, respectively. It had proved that all of them could efficiently upregulate the expression levels of many inflammatory mediators by activating the nuclear factor‐kappaB (NF‐κB) and MAPK signaling pathway, thereby promoting neutrophil proliferation, maturation, and chemotaxis, and leading to severe inflammatory reactions.^[^ ^54^, ^55^ ^]^ And it also revealed the complex expression correlations among the 44 autophagy related genes (*p* value < 0.05) by Pearson correlation analysis (Figure S17, Supporting Information). Additionally, as shown in Figures S18 and S19, Supporting Information, there was a significant correlation between DEGs and autophagy and Keap1/Nrf‐2/HO‐1 pathways related genes. Among them, most of DEGs were positively correlated with autophagy related genes, while a small number of DEGs were negatively correlated with them. After calculation, the absolute average correlation coefficient between DEGs and autophagy related genes was 0.442. Meanwhile, the absolute average correlation coefficient between DEGs and Keap1/Nrf‐2/HO‐1 pathways genes was 0.570. Both indicated a strong correlation between them. Prominently, by multidimensional scaling (MDS) analysis, the expression patterns of DEGs were consistent with those related to autophagy or Keap1/Nrf‐2/HO‐1 pathway. It manifested as partial overlap in 2D spatial distribution and maintained some degree of interconnection, but was relatively dispersed in expression patterns, showing high heterogeneity (Figures S20 and S21, Supporting Information). Subsequently, the intersection of 44 autophagy, 1753 ROS clearance, and 2650 anti‐inflammation related genes was implemented to obtain 8 intersection genes (Figure S22, Supporting Information). The 8 genes not only participated in cellular autophagy, but also had the function of ROS clearance and anti‐inflammation. Furthermore, as shown in Figures S23 and S24, Supporting Information, there was a significant correlation between the expression levels of Keap1/Nrf‐2/HO‐1, NF‐κB and phosphoinositide 3‐kinase/protein kinase b (PI3K/Akt) pathway related genes. Most Keap1/Nrf‐2/HO‐1 pathway genes were positively correlated with NF‐κB and PI3K/Akt pathways related genes, while a small number of DEGs were negatively correlated with them. At last, based on GeneMANIA analysis, the interaction network diagram of Keap1/Nrf‐2/HO‐1 pathway related genes was shown in Figure S25, Supporting Information. There were 20 genes related to Keap1/Nrf‐2/HO‐1 pathway predicted. Most genes were associated with co‐expression and physical interaction, while a small number were associated with co‐localization and pathway sharing. From the above, it provided a proof that Keap1/Nrf‐2/HO‐1 pathway activation together with macrophage M2 polarization, and NF‐κB and PI3K/Akt pathways inhibition mediated by CP+NIR could be the possible mechanism for SALI therapy.

Previous studies confirmed that Keap1/Nrf‐2/HO‐1 signaling pathway induced cellular autophagy to alleviate liver injury.^[^ ^56^ ^]^ Therefore, activating Keap1/Nrf‐2/HO‐1 signaling pathway could be helpful to alleviating inflammation storm and promoting liver injury repair. In cellular levels, HO‐1 expression level was initially investigated by immunofluorescent staining. As illustrated in Figure 5E,F, the HO‐1 expression (green fluorescence) was in highest level for CP+NIR (73.88 ± 3.32), followed by CP (47.72 ± 1.91), control group (35.38 ± 0.76), NIR (33.59 ± 2.28), and normal group (26.14 ± 2.19). Similarly, the HO‐1 gene expression was 0.28 ± 0.01, 0.39 ± 0.05, 0.53 ± 0.03, 4.92 ± 2.21, 0.31 ± 0.03, and 9.35 ± 1.46 for normal group, control group, CeO_2_, CP, NIR, and CP+NIR by RT‐qPCR, respectively (Figure 5G). And the other keap1/Nrf‐2/HO‐1 pathway related genes expression levels were also illustrated in Figure S26, Supporting Information. For Keap1 gene expression, it was in high levels for control group (1.46 ± 0.05) and NIR (1.50 ± 0.04), sequentially decreased to 1.00 ± 0.03 for normal group, 0.75 ± 0.03 for CeO_2_, 0.62 ± 0.01 for CP, and 0.13 ± 0.01 for CP+NIR (Figure S26A, Supporting Information). On the contrary, for normal group, glutamate‐cysteine ligase modifier subunit​​ (GCLM) and NAD(P)H quinone dehydrogenase 1​​ (NQO1) gene expression levels were 1.00 ± 0.04 and 1.00 ± 0.02, slightly declined to 0.70 ± 0.05 and 0.16 ± 0.04 for control group. NIR irradiation almost did not change their expression levels (0.71 ± 0.02 and 0.17 ± 0.01), while CeO_2_ (1.22 ± 0.03 and 1.98 ± 0.16), CP (1.37 ± 0.03 and 3.49 ± 0.13), and CP+NIR (1.69 ± 0.02 and 4.52 ± 0.11) could effectively ascended their expression levels. Among them, CP+NIR played the most efficient roles (Figure S26B,C, Supporting Information). The above results indicated that CP+NIR affected Nrf‐2 gene expression by downregulating Keap1 gene, and upregulating GCLM and NQO1 genes.

Besides, the expression levels of pathway related proteins were also investigated by western blotting (WB), where glyceraldehyde‐3‐phosphate dehydrogenase (GAPDH) or β‐actin (Proteintech, USA) was applied as internal reference. For Nrf‐2 protein, it was in relative high level for CP+NIR with the Nrf‐2/β‐actin ratio of 0.96 ± 0.25, decreased for control group (0.09 ± 0.14) and NIR (0.07 ± 0.08), and significantly ascended for CP (0.44 ± 0.21) and normal group (0.36 ± 0.16). Similarly, the HO‐1, LC3B and Beclin‐1 expression levels were low for normal group (0.15 ± 0.04, 0.25 ± 0.01, and 0.08 ± 0.04), slightly increased for control (0.32 ± 0.22, 0.23 ± 0.07, and 0.04 ± 0.04) and NIR (0.23 ± 0.08, 0.33 ± 0.04, and 0.12 ± 0.05), and obviously increased for CP (0.73 ± 0.11, 0.81 ± 0.18, and 0.61 ± 0.12) and CP+NIR (0.93 ± 0.10, 1.11 ± 0.16, and 1.09 ± 0.25), respectively. Conversely, for sequestosome 1 (p62) expression, it was in the lowest level for normal group (0.20 ± 0.04), significantly ascended for control group (0.98 ± 0.21) and NIR (0.98 ± 0.28). However, compared to control group, the p62 expression level obviously decreased for CP (0.64 ± 0.22) and CP+NIR (0.45 ± 0.13). respectively (Figures S27 and S28, Supporting Information). Meanwhile, chloroquine (CQ) was applied as the autophagy/lysosome inhibitor to confirm the autophagy mediated anti‐inflammation mechanism.^[^ ^57^, ^58^ ^]^ As shown in Figures S29 and S30, Supporting Information, compared to control group, CQ did not affect LC3B and HO‐1 expression levels. They were obviously enhanced for CP+NIR while CQ+CP+NIR showed no clear changed compared to control group. After statistical analysis, the relative LC3B/β‐actin ratio and HO‐1/β‐actin ratios were 0.20 ± 0.12 and 4.09 ± 0.21 for control group, changed to 0.17 ± 0.10 and 4.66 ± 0.33 for CQ, 0.55 ± 0.32 and 4.58 ± 0.26 for CP+NIR, and 0.20 ± 0.12, and 3.59 ± 0.17 for CQ+CP+NIR, respectively. Prominently, the other autophagy related proteins expression levels of treated cells with or without CQ were also analyzed by WB.^[^ ^59^, ^60^, ^61^ ^]^ As displayed in Figures S31 and S32, Supporting Information, the relative ATG5/GAPDH, LC3BII/LC3BI and ATG7/β‐actin ratios were almost the similar levels for control group (0.58 ± 0.03, 0.20 ± 0.11, and 4.09 ± 0.21), CQ (0.52 ± 0.02, 0.17 ± 0.10, and 4.66 ± 0.33) and CQ+CP+NIR (0.60 ± 0.03, 0.59 ± 0.17, and 3.59 ± 0.17). CQ itself, as the inhibitor, did not affect the autophagy related proteins expression levels of LPS treated cells. And it also inhibited the autophagy related proteins expression levels of LPS treated cells after incubating with CP+NIR, also confirming the autophagy related anti‐inflammation mechanism of CP+NIR.

Meanwhile, autophagosomes fuse with lysosomes to form autolysosomes and degrade their contents, and their effective fusion reflects the occurrence of cellular autophagy.^[^ ^62^, ^63^ ^]^ Thus, the co‐localization of lysosomes and autophagosomes (LC3B expression) was investigated by confocal scanning microscopy. As displayed in Figure S33, Supporting Information, the degree of overlapping between lysosome (red) and LC3B (green) was low for control group while it was almost complete overlapped for CP+NIR. CP+NIR markedly promoted the co‐localization of lysosome and autophagosomes compared to control group. And during the inflammatory response, Nrf‐2 is released from the complex, and translocated to the nucleus, where it forms heterodimers with antioxidant genes, and induces the production of NQO1 and HO‐1.^[^ ^64^, ^65^ ^]^ As shown in Figure S34A, Supporting Information, compared to normal group, control group and NIR with few green fluorescence (Nrf‐2 protein) in the nucleus, obvious green fluorescence happened in the nucleus of CP and CP+NIR. After statistical analysis, the intranuclear MFI was 0.34 ± 0.06, 0.43 ± 0.10, and 0.47 ± 0.10 for normal group, control group, NIR, enhanced for CP (1.12 ± 0.05) and CP+NIR (2.15 ± 0.45) (Figure S34B, Supporting Information). The obvious nuclear translocation of Nrf‐2 indicated the enhanced autophagy happened for CP+NIR. Similarly, by chromatin immunoprecipitation assay (ChIP), compared to control group with no bands (Nrf‐2) observed, an obvious band was displayed for the eluent of CP+NIR. For IgG as the positive control, no bands were shown ≈110 kDa, corresponding to the isomers of Nrf‐2 (Figure S35A, Supporting Information). After RT‐qPCR, the Nrf‐2/input ratio was relative low in IgG (0.05 ± 0.01) and eluent (0.10 ± 0.13) of control group, and IgG of CP+NIR (0.21 ± 0.27), significantly ascended to 1.95 ± 0.20 for the eluent of CP+NIR (Figure S35B, Supporting Information). It also confirmed that CP+NIR significantly enhanced the binding of Nrf‐2 to the antioxidant gene HO‐1.

Furthermore, generally, autophagy is a dynamic cellular degradation process that involves multiple key steps. Thus, different concentration of CP+NIR or incubation time of CP+NIR was considered as the therapeutic conditions to investigate the autophagy related proteins expression of treated cells. As illustrated in Figures S36 and S37A, Supporting Information, compared to control group, the LC3B expression increased, and p62 expression decreased with the increased of CP+NIR concentrations from 20 to 100 µg mL^−1^. After statistical analysis, the relative LC3BII/LC3BI ratio was 1.27 ± 0.01 and 1.18 ± 0.01 for normal group and control group, increased to 1.57 ± 0.07, 2.14 ± 0.02, and 2.63 ± 0.07 for CP+NIR with the concentration of 20, 50, and 100 µg mL^−1^. Conversely, the relative p62/GAPDH ratio was 0.40 ± 0.02 in normal group, jumped to 0.63 ± 0.02 for control group. And it began to decrease to 0.51 ± 0.02, 0.38 ± 0.02, and 0.33 ± 0.02 with the increase of CP+NIR concentrations from 20, 50, to 100 µg mL^−1^ (Figure S37B, Supporting Information). In the meantime, the therapeutic time was also considered. As shown in Figures S38 and S39, Supporting Information, the LC3B expression was ascended and p62 expression was declined compared to control group with the increased of incubation time with the same concentration of CP+NIR. The relative LC3BII/LC3BI ratio was 0.40 ± 0.04 for control group, sequentially increased to 0.54 ± 0.06, 0.73 ± 0.05, 0.80 ± 0.07, and 0.13 ± 0.07 for CP+NIR with the incubation time of 4, 8, 12, and 24 h. However, the relative p62/GAPDH ratio decreased with the increase of incubation time, which was 0.24 ± 0.01, 0.21 ±0.01, 0.18 ± 0.01, and 0.14 ± 0.01 for CP+NIR with the incubation time of 4, 8, 12, and 24 h compared to control group (0.34 ±0.01) (Figure S39B, Supporting Information). Under certain circumstances, increasing the concentration of CP+NIR and its treatment time could both be effective to increase the relative LC3BII/LC3BI ratio, and decreased the relative p62/GAPDH ratio, promoting autophagy activation. In addition, the effects of CP+NIR on other classic inflammatory pathways like NF‐κB, janus kinase‐signal transducer and activator of transcription (JAK‐STAT) and NLRP3 were also explored. As shown in Figure S40, Supporting Information, for p65 gene expression, it was in the similar levels for all groups with no significant differences, while CeO_2_ (1.63 ± 0.13), CP (1.19 ± 0.02) and CP+NIR (1.05 ± 0.03) could decrease the gene expression of cyclooxygenase‐2 (COX‐2) compared to control group (2.19 ± 0.13) and NIR (2.26 ± 0.34). And for JAK‐STAT pathway, the interferon regulatory factor‐1 (IRF‐1) gene expression was in high levels for control group (1.19 ± 0.03) and NIR (1.15 ± 0.04), sequentially reduced for normal group (1.00 ± 0.03), CeO_2_ (0.78 ± 0.10), CP (0.41 ± 0.02), and CP+NIR (0.16 ± 0.06) (Figure S41A, Supporting Information). On the contrary, the suppressor of cytokine signaling 1 (SOCS1) gene expression was in the relative low levels for control group (0.37 ± 0.04) and NIR (0.40 ± 0.04), which was significantly ascended to 1.01 ± 0.21 for normal group, 1.64 ± 0.04 for CeO_2_, 2.16 ± 0.18 for CP, and 2.67 ± 0.24 for CP+NIR, respectively (Figure S41B, Supporting Information). Remarkably, the NLRP3 gene expression level was high for control group (1.70 ± 0.20) and NIR (1.79 ± 0.10) compared to normal group (1.00 ± 0.03). However, their expression levels were obviously reduced to 0.54 ± 0.03, 0.44 ± 0.01, and 0.14 ± 0.09 for CeO_2_, CP and CP+NIR, respectively (Figure S42, Supporting Information). It gave the proof that all treatment groups except for NIR could efficiently achieve anti‐inflammation via inhibiting NF‐κB (COX‐2 downregulation),^[^ ^66^ ^]^ JAK‐STAT (IRF‐1 downregulation and SOCS1 upregualtion),^[^ ^67^ ^]^ and NLRP3 (NLRP3 downregulation)^[^ ^68^ ^]^ pathways. Among all treatment groups, CP+NIR was the most effective.

Finally, the relative mechanism for SALI therapy was also confirmed in animal levels. As shown in Figure 5I,J, the HO‐1 expression was in high level for CP+NIR (2.72 ± 0.35) with obvious brown sites observed, followed by CP (1.24 ± 0.04), NIR (0.35 ± 0.14), SALI (0.24 ± 0.03), and sham group (0.05 ± 0.01). Significantly, SEM was applied to analyze the microstructure of liver tissue. Compared to the liver of sham group, server edema happened to liver cells of SALI, with local damaged cell membranes, reduced number of organelles, and severe swelling or disintegration of mitochondria. However, the edema degree was reduced with intact cell membrane and visible abundance of autophagosomes for CP+NIR (Figure 5H). Significantly, the pathway protein expression of liver was also investigated by WB. As imaged in Figure S43, Supporting Information and Figure 5K, the low levels of HO‐1 happened for sham group, SALI, NIR with the HO‐1/β‐actin ratio of 0.64 ± 0.15, 0.58 ± 0.10, and 0.56 ± 0.19, respectively. And it significantly ascended to 0.79 ± 0.07 and 1.37 ± 0.08 for CP and CP+NIR, consistent with the corresponding protein expression levels in cellular level. Similarly, the Nrf‐2, LC3B and Beclin‐1 expression were in highest levels for CP+NIR (0.94 ± 0.20, 0.87 ± 0.03, and 1.19 ± 0.06), which still maintained a certain of levels for sham group (0.47 ± 0.11, 0.59 ± 0.20, and 0.67 ± 0.25) and CP (0.52 ± 0.06, 0.76 ± 0.06, and 0.70 ± 0.19), and were in relative low levels for SALI (0.12 ± 0.04, 0.32 ± 0.05, and 0.45 ± 0.04) and NIR (0.07 ± 0.05, 0.40 ± 0.14, and 0.47 ± 0.12).

Altogether, it had confirmed that the strategy of CP+NIR efficiently achieve SALI immunotherapy via inducing macrophage M2 polarization and autophagy to reprogram the redox homeostasis of liver microenvironment by transcriptional gene analysis. In cellular level and animal model, HO‐1, Nrf‐2, LC3B and Beclin‐1 gene and protein, and GCLM and NQO1 gene expression were in the high levels for CP+NIR compared to other groups. Specifically, it was also observed that a huge abundant of autophagosomes were visible for CP+NIR. Significantly, the pathway related proteins of Nrf‐2, HO‐1, LC3B and Beclin‐1 were in high levels, and Keap1 and p62 was in low levels for CP+NIR, indicating the activation of cellular autophagy pathway.^[^ ^69^, ^70^ ^]^ Meanwhile, the effect of CP+NIR on other related pathways including NF‐κB, JAK‐STAT and NLRP3 also contributed to anti‐inflammation and accelerated tissue repair.

## 3 Conclusions

In summary, a Pd doped CeO_2_ nano‐heterojunction (CP) was developed to act as a catalytic nanomedicine with enhanced ROS scavenging capacity for SALI immunotherapy. It had confirmed that CP possessed multiple enzymatic activities and favorable cell viability and hemocompatibility. Combining with NIR irradiation, it displayed the demoted intracellular ROS levels, downregulation of inflammatory factors, upregulation of antioxidant and anti‐inflammatory factors, induced macrophage M2 directional polarization, and accelerated tissue repair, to achieve enhanced pulmonary redox homeostasis reprogramming for SALI therapy. In the meantime, CP+NIR strengthened the upregulation of HO‐1, Nrf‐2, LC3B and Beclin‐1 expression levels, and downregulation of Keap1 and p62 expression, leading to activating Keap1/Nrf‐2/HO‐1 pathway, therapy inducing cellular autophagy. This work provides a strategy of CP+NIR with multiple enzymatic activities to systematically manage SALI with high efficacy and biosafety, which extends the design of developing multi‐functional nanozymes for ROS related diseases immunotherapy.

## 4 Experimental Section

### Materials and Chemicals

Anhydrous ethanol (99.5%) and cerium dioxide (CeO_2_, 99.9%) were commercially purchased from Macklin (China). L‐ascorbic acid and potassium hexachloropalladate (IV) (K_2_PdCl_6_) were commercially obtained from Acmec (Shanghai, China). Hydrogen peroxide (H_2_O_2_, 30% w/w) was obtained from Nanning Fuxiang Scientific Instruments Co. Ltd. Methanol (≥ 99.9%), CQ (97%) and acetic acid (99.5%) were commercially obtained from Aladdin (Shanghai, China). And phosphate buffered saline (PBS, pH = 7.4) was purchased from Solarbio (China). Sodium chloride was obtained from Junobio (Nanning, China). All chemicals were directly utilized without purification.

### Preparation and Physicochemical Characterization of CP

CP was fabricated by the simple previously reported method. Briefly, 5 mL Ce(OAc)_3_ (5 mM) and 5 mL K_2_PdCl_6_ (5 mM) were mixed in 150 mL DI water followed by slow drop‐wised addition of 40 mL ascorbic acid solution (14.2 mM). The mixture reacted for 4 h till the mixture became dark. After centrifuge at 12 000 rpm min^−1^ for 10 min and re‐dispersion against with DI water, the product (CP) was collected by vacuum drying, and saved for further experiments.

CeO_2_ and CP were characterized by zeta sizer (Nano ZS90, Malvern, UK) to test the zeta potential. And the morphology, elemental distribution, and 3D structure of CP were investigated by TEM (Hitachi, Japan) combined with energy‐dispersive X ray spectroscopy (EDS, Hitachi, Japan), and AFM (Bruker, USA). Besides, CP was also characterized by XPS (ESCALAB 250XI+, USA) and ICP‐MS, (Varian, USA) to investigate the elemental composition and metal element contents. Furthermore, CP was also characterized by ultraviolet‐visible spectroscopy (UV–vis, Shimadzu, Japan), XRD (MiniFlex 600, Japan), and Raman spectroscopy (Raman, HORIBA HR Evolution, France) to investigate their chemical structure, crystallization structure and molecular structure, respectively. Finally, CP was dispersed in PBS (pH = 7.4), DMEM (Gibco, USA), FBS (Every Green, China), or 5 mM H_2_O_2_ in PBS, and then imaged by camera at 0, 0.5, 1, 2, 6, 12, and 24 h, respectively.

In vitro photothermal effects investigation was implemented by placing CP with different concentration of 0, 100, 200 or 500 µg mL^−1^ with NIR irradiation of different power intensity (808 nm, 1, 1.5, or 2 W cm^−2^) for 10 min. Specially, for photothermal stability, 200 µg mL^−1^ CP was irritated with 5 repeated “on” and “off” cycles with NIR light (2 W cm^−2^). The images were collected, and temperatures were recorded by thermal imager (FLIR, USA).

### ROS Scavenging Investigation

ROS scavenging ability was initially investigated by electron spin resonance (ESR, Bruker A300, Germany). Briefly, 5‐tert‐butoxycarbonyl 5‐methyl‐1‐pyrroline‐N‐oxide (BMPO, 100 mM), xanthione (10 mM) and xanthione oxidase (XOD, 1 U mL^−1^), and 2, 2, 6, 6‐Tetramethylpiperidine (TEMPONE, 100 mM) were respectively applied as the working solutions for ·OH, ·O_2_^−^, and ^1^O_2_ scavenging testing. After mixing with 100 µg mL^−1^ CeO_2_ or CP for 10 min, the corresponding solutions were observed by ESR. In particular, for CP+NIR, NIR irradiation (1 W cm^−2^) was implemented for 10 min after mixing the working solutions with CP. Besides, ROS scavenging capacities were investigated by ROS testing kits (Beyotime, China) including superoxide anion (·O_2_^−^), hydroxyl free radical (·OH), CAT assay kits by following the protocols. In brief, CeO_2_ and CP of 50, 100, and 200 µg mL^−1^ were mixed with testing solutions. After waiting for 30 min, the supernatant of mixtures was observed at 560, 550, and 520 nm, respectively by microplate reader (Thermo Scientific, USA) for ·O_2_^−^, ·OH and H_2_O_2_ levels testing. In specific, for NIR or CP+NIR, NIR irradiation was implemented for 10 min (1 W cm^−2^) before observation.

### Cell Culture and Cell Biocompatibility

RAW264.7 were directly obtained from American type culture collection (ATCC, USA), and cultured in DMEM containing 10% FBS and 1% penicillin/streptomycin (Procell, China). When reaching 85% confluence, RAW264.7 were passaged, and the third passage was utilized for further experiments.

Cell viability was initially evaluated by using cell counting kit‐8 (CCK‐8, Biosharp, China) by following the protocols. In details, RAW264.7 were cultured in 96‐well plate with the density of 10 000 cells per well. Later, the cultured medium was replaced with 100 µL mixture containing CeO_2_ or CP of 0, 5, 10, 20, 50, 100, 200, and 500 µg mL^−1^, respectively. After 24 h’ incubation, cells were washed against with PBS for three times before adding 100 µL 10% CCK‐8 solution for another 2 h. Finally, the supernatant was observed at 450 nm by spectrophotometer (Thermo Fisher, USA).

Besides, live/dead staining was implemented to investigate the protection ability for LPS (BD Pharmingen America) induced RAW264.7. Briefly, cells were treated with LPS (1 µg mL^−1^) for 30 min, and then incubated with 100 µg mL^−1^ CeO_2_ or CP for another 24 h. Specific for CP+NIR, NIR irradiation was implemented for three times (10 min per time, 1 W cm^−2^) at 0, 1, and 2 h after incubation. The corresponding groups were: cells without treatment (normal group), LPS induced cells followed by PBS treatment (control group), LPS induced cells followed by 100 µg mL^−1^ CeO_2_ treatment (CeO_2_), LPS induced cells followed by 100 µg mL^−1^ CP treatment (CP), LPS induced cells followed by NIR irradiation (NIR), and LPS induced cells followed by 100 µg mL^−1^ CP combining with NIR irradiation (CP+NIR). Later, the treated cells were washed against with PBS for three times, and incubated with calcein‐AM/propidium iodide (PI) (Beyotime Biotechnology, China) before observation by fluorescent microscopy (Olympus, Japan).

Finally, the hemocompatibility of CP was implemented by hemolysis test. The fresh blood was obtained from C57BL/6J mice, and centrifuged at 3000 rpm for 10 min. After removing the supernatant, the pellet was re‐suspended in PBS, and 0.5 mL suspension was taken to mix with 0.5 mL DI water, or CP of 0, 5, 10, 20, 50 100, 200, 400, and 800 µg mL^−1^, respectively. After incubation for 1 h, the mixture was centrifuged (3000 rpm, 10 min), and the supernatant was collected and observed by microplate reader at 540 nm. The hemolysis ratio was calculated: [(OD_s_‐OD_n_)/(OD_p_‐OD_n_)]/100, where OD_n_, OD_p_ and OD_s_ was the optical density (OD) of PBS, DI water and samples, respectively.

### Cellular Uptake Investigation

At the beginning, 30 mg CP was well dispersed in 45 mL ethanol, followed by adding with 3 mg Cy5‐PEG2000‐Thiol (Lumiprobe, China). The mixture reacted for 24 h before centrifuge at 12 000 rpm for 30 min, and re‐dispersing in ethanol for three times. Cy5‐CP was isolated by vacuum drying. For cellular uptake investigation, RAW264.7 was cultured in confocal dishes before incubating with Cy5‐CP for 3 h. And the treated cells were washed against with PBS for three times, fixed with 4% paraformaldehyde (PFA, Biosharp, China), and stained with actin‐tracker green‐488 (actin, Biosharp, China) and 4, 6‐diamidino‐2‐phenyindole dilactate (DAPI, Biosharp, China), respectively. At last, the treated cells were washed with PBS buffer for three times before observation by confocal scanning microscopy (ZEISS, Germany). And the collected images were finally analyzed by Image J.

### Antioxidant and Anti‐Inflammatory Activities in Cellular Levels

Macrophages were initially treated by LPS (1 µg mL^−1^) for 30 min, and treated with 100 µg mL^−1^ CeO_2_ or CP overnight. The supernatant of treated cells was collected, and tested by ELISA kits (Meimian, China) to investigate the expression levels of IL‐6 and IL‐1β following the instruction of manufacture. Besides, the intracellular ROS levels of treated RAW264.7 were investigated by ROS testing probes. In details, the cultured cells were replaced with fresh medium containing HPF (maokangbio, China), DHE (maokangbio, China) or DCFA (maokangbio, China) for ·OH, ·O_2_^−^ or total ROS levels testing, respectively. After incubation for 30 min, the cells were rinsed with PBS for three times before PFA fixation and observation by fluorescent microscopy (ZEISS, Germany). In addition, the intracellular ROS levels of treated cells were also analyzed by flow cytometry (BD FACSCanto II, America). Next, the inflammation pathway related genes including inflammatory genes (TNF‐α, iNOS and IL‐1β), M1 type gene (CD68), M2 type gene (CD206), tissue repair gene (HSP70), autophagy pathway related genes (HO‐1, Keap1, GCLM and NQO1), NF‐κB pathway related genes (p65 and COX2), JAK‐STAT pathway related genes (IRF‐1 and SOCS1), and NLRP3 pathway related gene (NLRP3) expression levels were quantified analyzed by RT‐qPCR. The total RNA was extracted from treated cells by RNA extraction kit (Magen, China), and qRT‐PCR was implemented by using LightCycler System (Roche, Switzerland), analyzed by 2^−ΔΔCt^ method and compared with β‐actin (ACTB, Thermo Fisher). The primer sequences for RT‐qPCR were detailed illustrated in Table S6, Supporting Information. Furthermore, the related proteins expression levels were investigated by immunofluorescence staining. Detailed, the treated cells were fixed by PFA for 30 min, and blocked with goat serum before incubation with primary antibody: IL‐6, IL‐1β or HO‐1 (1:200 dilutions, Proteintech, China) at 4 °C overnight. The cells were incubated with secondary antibody (FITC conjugated AffiniPure Goat Anti‐Rabbit IgG) (Boster, China) for another 1 h before washing against with PBS for three times. And the treated cells were stained with DAPI for 10 min. After PBS buffer washing, the cells were imaged by fluorescent microscope.

### Anti‐Inflammation Related Mechanisms Exploration

RNA sequencing was applied to study the collection of all intracellular transcripts. In brief, LPS induced cells were cultured, and treated with PBS (control group) or 100 µg  mL^−1^ CP+NIR (CP+NIR) for 24 h, respectively. The total RNA was extracted by RNA extraction kit (Magen, China), and genomic DNA was digested by using DNase I (Takara, Japan) before performing high throughput RNA sequencing. The corresponding sequencing analysis was implemented to identify the DEGs using R software (version = 4.42) with the statistical significance of *p* value < 0.05 and Log_2_ Fold change > 1. Based on the sequencing results, the data cleaning and standardization was performed. Subsequently, differential analysis was conducted on standardized gene expression to screen DEGs, further used for KEGG and GO enrichment analysis to explore the possible pathways about CP+NIR in SALI therapy. To further explore the relationship between anti‐inflammation and Keap1/Nrf‐2/HO‐1 pathway, the Pearson correlation and MDS analysis were conducted. Based on the mean absolute correlation coefficient and the visualized scattering plots, their expression correlation was explained. At last, in order to explore how autophagy regulated oxidation stress and inflammation, autophagy related genes were identified from DEGs. And the gene sets of “(Oxidative stress) AND” Homo sapiens “[porgn: __txid9606]” and “(Inflammation) AND” Homo sapiens “[porgn: __txid9606]” in gene expression omnibus (GEO) database were retrieved, and intersected with autophagy related DEGs. Thus, the Venn diagrams were drawn to reflect which genes autophagy acted on oxidative stress and inflammation regulation. Meanwhile, to further investigate the crosstalk mechanisms between Keap1/Nrf2/HO‐1 pathway and other signaling pathways, two classic anti‐inflammatory pathways NF‐κB and PI3K‐Akt, were chosen for analysis. And Pearson correlation analysis was performed between genes of the two pathways and those of Keap1/Nrf2/HO‐1 pathway. Additionally, GeneMANIA analysis was employed to predict the interactions among pathways related genes, enabling the construction of a protein‐protein interaction network.

To confirm the anti‐inflammation mechanism, WB was implemented to detect the proteins expression levels of treated cells. In brief, the treated cells were washed against with ice‐cold PBS, and treated with lysis buffer containing protease inhibitors and phosphatase inhibitors. And the supernatant was collected, and the protein concentration was tested by BCA protein assay kit (Beyotime, China). And then supernatant was separated by implementing protein gel electrophoresis, and transferred to polyvinylidene fluoride (IPVH00010, Millipore, USA) membranes. After blocking for 2 h and rinsing with tris buffered saline with Tween‐20 (TBST, Sigma, US) for three times, the membranes were incubated with primary antibody (anti‐Nrf‐2, anti‐HO‐1, anti‐LC3B, anti‐Beclin‐1, anti‐p62, anti‐ATG5, and anti‐ATG7, Proteintech, China), respectively overnight at 4 °C. After washing against with TBST for three times, the membranes were incubated with secondary antibody (Goat Anti‐Rabbit, Sangon, China) for 2 h. Finally, the strips were soaked in 1 mL of UltraSignal ECL Western Blotting Detection Reagent (Beyotime, China), and scanned by automatic chemiluminescence image analysis system (Bio‐Rad, USA). The relative proteins expression levels were compared with GAPDH or β‐actin. In particular, the cells were treated with LPS followed by incubating with 100 µg mL^−1^ CP+NIR for different time points (4, 8, 12, and 24 h) or different concentrations (20, 50, and 100 µg mL^−1^) of CP+NIR for 12 h. After extracting the proteins, the expression levels of p62, LC3BI and LC3BII in treated cells were analyzed by WB. Besides, the LPS induced cells were treated with CQ (50 µM) for 12 h followed by incubating with 100 µg mL^−1^ CP+NIR overnight. The expression levels of HO‐1, LC3BI, LC3BII, ATG5 and ATG7 were also analyzed by WB.

To further confirm the autophagy related anti‐inflammation mechanism, the co‐localization of lysosome and autophagy related protein (LC3B) was also investigated. Briefly, the cultured cells were treated with LPS followed by incubating with 100 µg mL^−1^ CP+NIR overnight. After washing against with PBS for three times, the cells were fixed with PFA, and sequentially stained with lysosome probe (BBcellProbe L05, Bestbio, China) for 30 min. And then, the cells were incubated with 50 µL LC3B Rabbit mAb (Zenbio, China) at 4 °C overnight. After rinsed with PBS for three times, the cells were incubated with 200 µL FITC conjugated AffiniPure Goat Anti‐Rabbit IgG (H+L) for another 1 h. The final sample were observed by confocal scanning microscopy after DAPI staining. Specifically, for nuclear translocation co‐localization investigation, the cells were incubated with 50 µL Nrf‐2 Rabbit mAb (Zenbio, China) as the primary antibody before observation. Prominently, ChIP was applied to investigate the relationship between protein modification and gene expression. In detail, the treated cells were fixed with 37% formaldehyde (Sigma, USA) at 37 °C for 10 min. The reaction was stopped by 1.1 mL glycine solution (Beyotime, China) per well at ice bath. And the cells were washed with cold PBS containing 1 mM phenylmethylsulfonyl fluoride (PMSF, Sigma, USA) for three times. The cells were collected after centrifuge at 4 °C (8000 rpm, 2 min), and re‐suspended with SDS lysis buffer (Beyotime, China) containing 1 mM PMSF for 10 min. After ultrasonification for 10 min, 10 µL samples were saved at 65 °C for 3 h, and their DNA were extracted by phenol‐chloroform (Sigma, USA) extraction method for DNA gel electrophoresis (1.5% agarose gel (Sigma, USA), 100 V for 2 h). In addition, the samples were mixed with Protein A+G agarose (Beyotime, China) for 30 min before centrifuge. Later, the supernatant was mixed with Nrf‐2 Rabbit mAb or Rabbit IgG Isotype control (Zenbio, China) at 4 °C overnight. And then, the samples were mixed with Protein A+G Agarose at 4 °C for 1 h, and the supernatant was removed after centrifuge. The agarose beads were sequentially washed with low salt washing buffer, high salt washing buffer, LiCl washing buffer and Tris‐EDTA (TE) buffer (Beyotime, China), and dispersed with elution buffer (1% SDS and 0.1 M NaHCO₃). The final solution was collected after centrifuge at 4 °C (8000 rpm, 1 min), and the DNA was extracted by phenol‐chloroform extraction method. The relative Nrf‐2 gene expression levels after ChIP were analyzed by RT‐qPCR.

### In Vivo Biodistribution, Photothermal Effect and Biosafety

In vivo animal experiment was conducted under the approval of ethics committee of animal experiments of Guangxi Medical University (No. 202 306 191). C57BL/6J mice (≈20–30 g, n ≥ 6) were selected and treated for animal experiment. To evaluate in vivo biodistribution, in vivo animal imaging system (IVIS, Pekin Elmer, US) was applied after intravenous (IV) injection of 0.8 mL Cy5 or Cy5‐CP (100 µg mL^−1^). The images were collected at 0.1, 0.5, 1, 2, 4, 8, and 24 h by IVIS with the excitation and emission wavelength at 646 and 664 nm. And in vivo photothermal effects were implemented by IV injection of 0.8 mL CeO_2_ or CP (100 µg mL^−1^) into mice. After 30 min, the liver of mice was irradiated by NIR light for 15 min. And the photothermal images and the corresponding temperatures were collected and recorded at predetermined time points by thermal camera.

To evaluate in vivo biosafety, mice were IV injected with 0.8 mL PBS (sham group) or CP (100 µg mL^−1^) (CP), respectively. The body weight of each mouse weighed every day for 7 days. After 7 days, the blood samples of mice were respectively analyzed by blood routine testing (fully automatic blood analyzer, BC‐6800Plus, Mindray, China), and biochemistry testing (fully automatic biochemical analyzer, 7600, HITACHI, China). Particularly, the major organs including heart, liver, spleen, lung and kidney were collected. After embedding, the organs were sectioned and stained by H&E before observation by Olympus microscopy.

### In Vivo SALI Therapy Evaluation

To establish SALI animal models, C57BL/6J mice were intraperitoneal injected with LPS (1 mg mL^−1^, 5 mg kg^−1^) for 2 h. And mice were separated divided into 6 groups (6 mice per group): mice without treatment (sham group), SALI mice with IV injection of saline (SALI group), SALI mice with IV injection of 0.8 mL, 100 µg mL^−1^ CeO_2_ (CeO_2_), SALI mice with IV injection of 0.8 mL, 100 µg mL^−1^ CP (CP), SALI mice with NIR irradiation, and SALI mice with IV injection of 0.8 mL, 100 µg mL^−1^ CP combining with NIR irradiation (CP+NIR). Specifically, NIR irradiation was irradiated for three times, 10 min per time at 0.5, 1, and 2 h, respectively after IV injection respectively for NIR and CP+NIR. After 24 h’ treatment, the blood samples and major organs were collected and saved for further application. The fresh blood was centrifuged, and serum was collected to test the IL‐6 and IL‐1β expression levels by ELISA. In addition, other organs were cut into sections and stained with H&E for Olympus microscopy observation.

Besides, the livers were cut into pieces and homogenized. After centrifuge, the supernatant was saved to detect the lipid peroxidation level by MDA detection kit (Solarbio, China). And the isolated livers were fixed by 4% PFA for 24 h, and cut into sections of 3 µm thickness for the following experiments. To observe the autophagosome of liver tissue, scanning electron microscopy (SEM, Thermo Fisher, USA) was applied. And the liver was also stained with H&E before observation. In addition, the ROS levels of liver tissues were investigated by using DHE probe following the instruction. The nuclei were stained with DAPI. After mounting, the corresponding slides were observed by fluorescent microscopy.

Significantly, for immunohistochemical staining, the slides were initially incubated with primary antibody (rabbit polyclonal anti‐IL‐6, anti‐IL‐10, anti‐HSP70 and anti‐HO‐1, Servicebio, China) at 4 °C overnight before washing against with PBS buffer for three times. And the slides were incubated with biotinylated secondary antibodies (Servicebio, China). After rinsing and sealing, the slides were imaged by Olympus microscopy, and the AOD was analyzed by Image J. To further investigate the anti‐inflammation mechanism, the proteins including Nrf‐2, HO‐1, LC3BI, LC3BII and Beclin‐1 expression levels in the livers of treated mice were also analyzed by WB.

### Statistical Analysis

All results were expressed as mean ± standard deviation, and the significance was defined as *: *p* < 0.05, **: *p* < 0.01, ***: *p* < 0.001, and ****: *p* < 0.0001, where * indicates comparison with normal group in cellular levels or sham group in animal levels.

## Conflict of Interest

The authors declare no conflict of interest.

## Supporting information



Supporting Information

Supporting Information

## Data Availability

The data that support the findings of this study are available from the corresponding author upon reasonable request.
