# Supplementary material for: NIR Driven Pd/Cerium Oxide Nano‐Heterojunction for Enhanced Salvaging Sepsis Induced Acute Liver Injury via Reprogramming Redox Homeostasis in Synergy with Inducing Autophagy
Source: Adv Sci (Weinh). 2025 Jun 29;12(32):e17252. doi: 10.1002/advs.202417252 (PMC12407304; doi:10.1002/advs.202417252)
Supplement: Supplementary file 1 — Supporting Information [file ADVS-12-e17252-s002.docx]

NIR driven Pd/cerium oxide nano-heterojunction for enhanced salvaging sepsis induced acute liver injury via reprogramming redox homeostasis in synergy with inducing autophagy

Tao Qin ^a 1^, Lian Qin ^b 1^, Yang Zhao ^c 1^, Yin Chen ^b^, Qianyue Liu ^d^, Xiaoguang Lin ^a^, Yongfeng Lan ^d^, Yaohui Huang ^d^, Yan Liu ^d, e^, Ke Zhang ^d^, Lifan Pan ^b^, Jiaxiao Li ^b^, Kunpeng Duan ^f^, Hao Liang ^d^, Mingjing Yin ^d^, Guiyang Fan ^g^, Lian Liu ^a^, Yu Deng ^a^, Lin Liao ^a^, Danke Su ^c *^, Ming Gao ^f *^, and Junyu Lu ^b *^

^a^ Department of Emergency, Guangxi Medical University Cancer Hospital, Nanning, Guangxi 530201, China

^b^ Intensive Care Unit, The Second Affiliated Hospital of Guangxi Medical University, Nanning, Guangxi 530007, China

^c^ Medical Imaging Center, Guangxi Medical University Cancer Hospital, Nanning, Guangxi 530021, China

^d^ Department of Clinical Laboratory, Key Laboratory of Clinical Laboratory Medicine of Guangxi Department of Education, The First Affiliated Hospital of Guangxi Medical University, Nanning, Guangxi 530021, China

^e^ Key Laboratory of Micro-Nanoscale Bioanalysis and Drug Screening, Education Department of Guangxi Zhuang Autonomous Region, Nanning, Guangxi 530021, China

^f^ Life Sciences Institute, Guangxi Medical University, Nanning, Guangxi 530021, China

^g^ Department of Anesthesiology, Guangxi Medical University Cancer Hospital, Nanning, Guangxi 530201, China

**Supporting information**

**
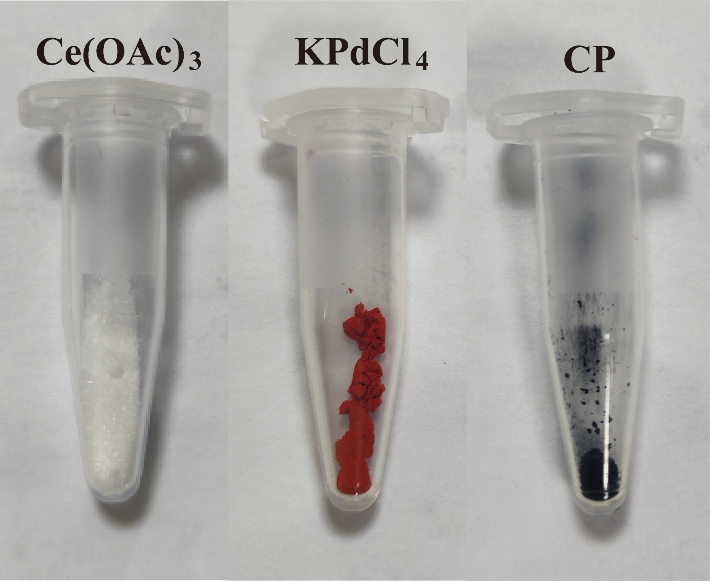
**

**Figure S1.** Optical image of Ce(OAc)_3_, KPdCl_4_ and CP.

**
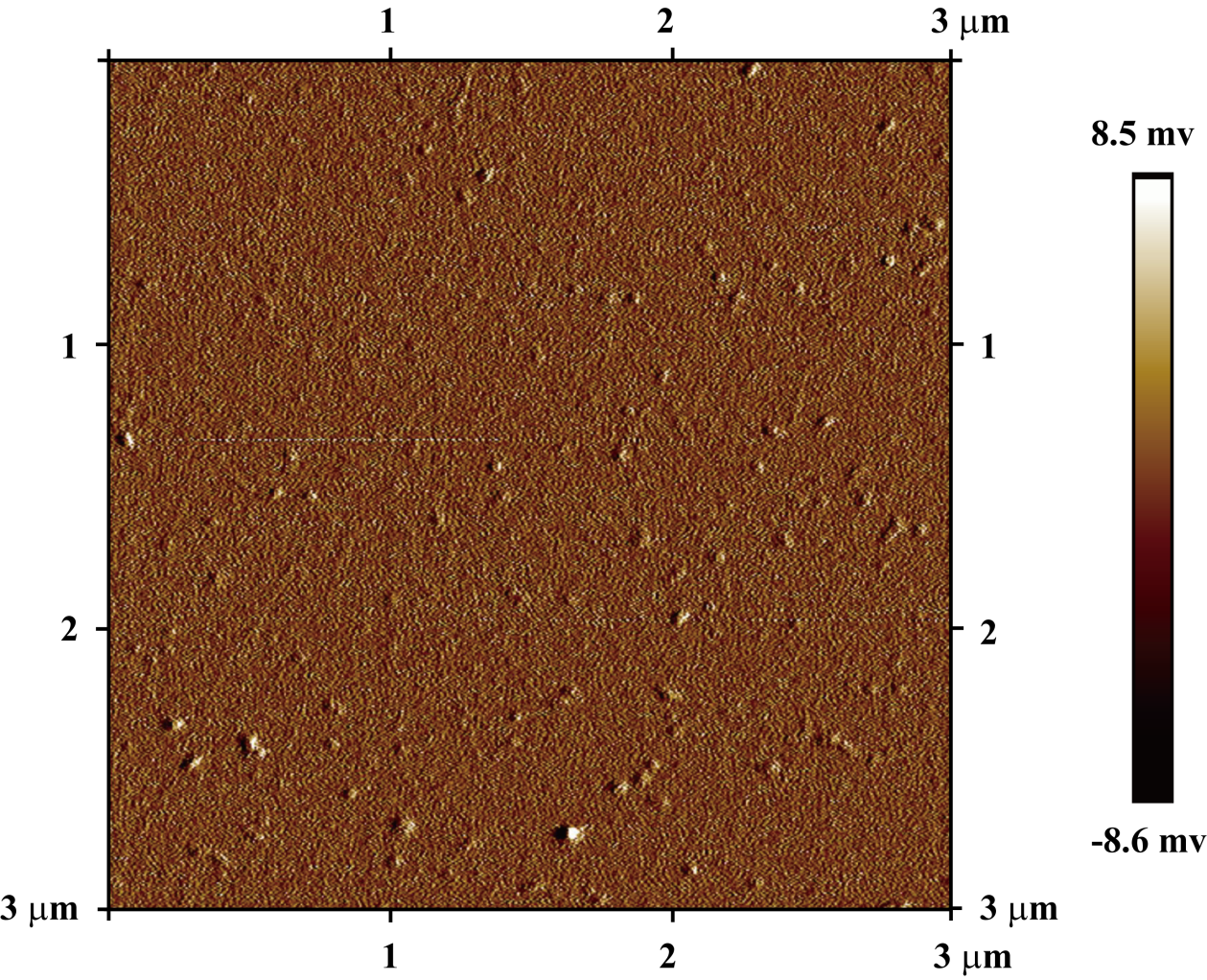
**

**Figure S2.** AFM image of CP.

**
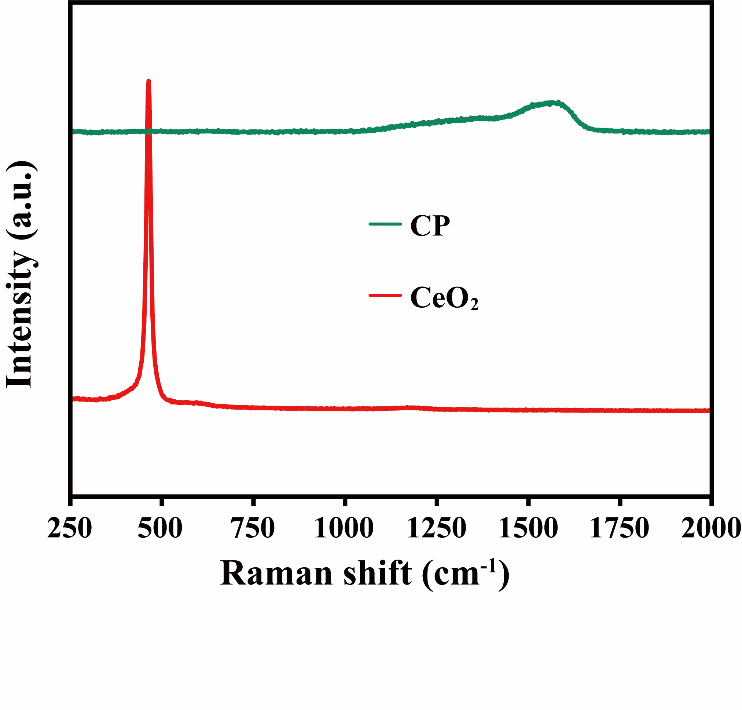
Figure S3.** Raman spectrum results of CeO_2_ and CP.

**
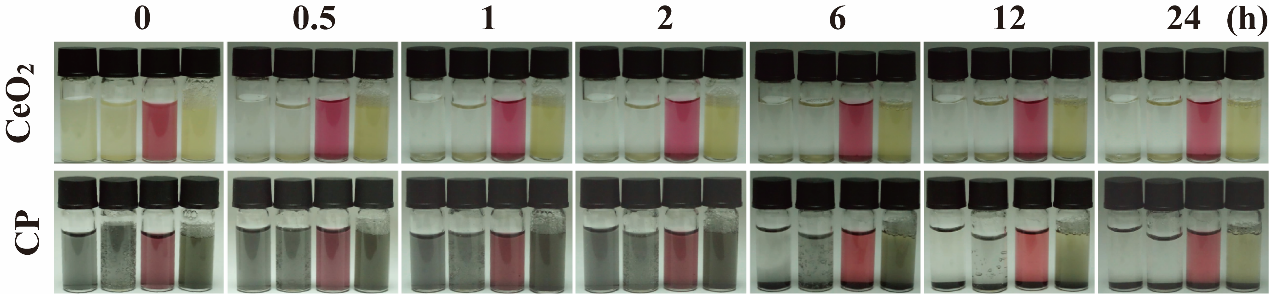
**

**Figure S4.** Dispersion and stability of CeO_2_ and CP in different solutions (from left to right: PBS, 5 mM H_2_O_2_, DMEM and FBS) at 0, 0.5, 1, 2, 6, 12 and 24 h.

**
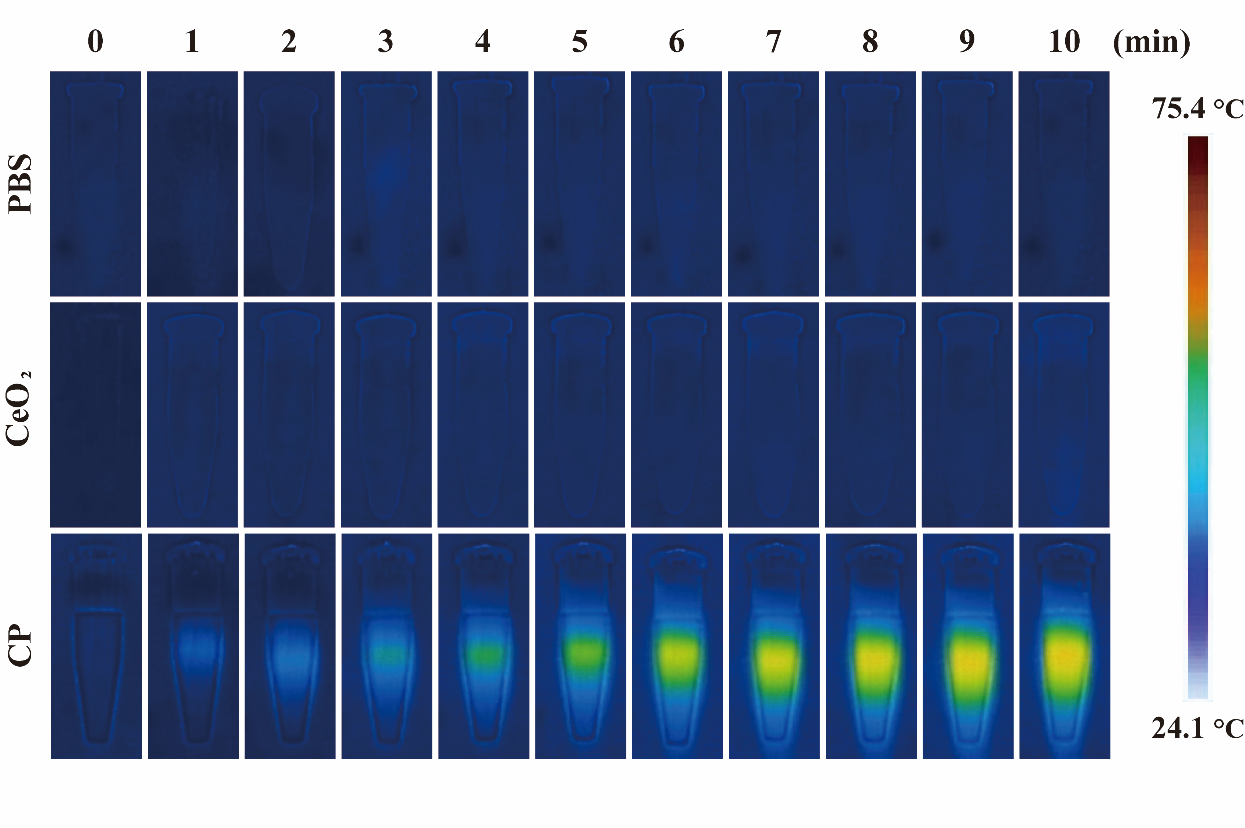
**

**Figure S5.** Photothermal images of PBS, CeO_2_ and CP with the same concentration of 200 μg/mL under NIR irradiation (2 W/cm^2^) versus time.

**
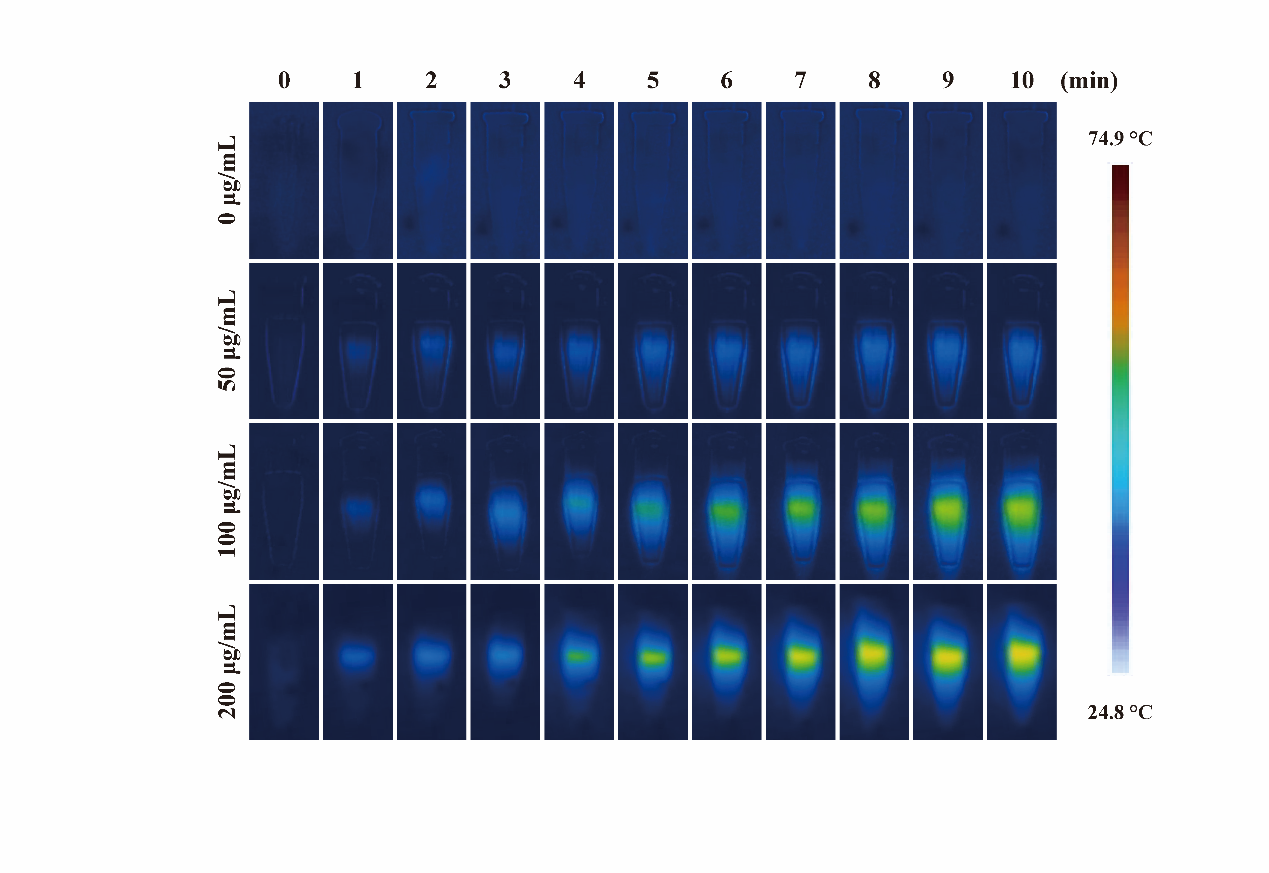
**

**Figure S6.** Photothermal images of different concentration (0, 50, 100 and 200 μg/mL) of CP under NIR irradiation (2 W/cm^2^) versus time.

**
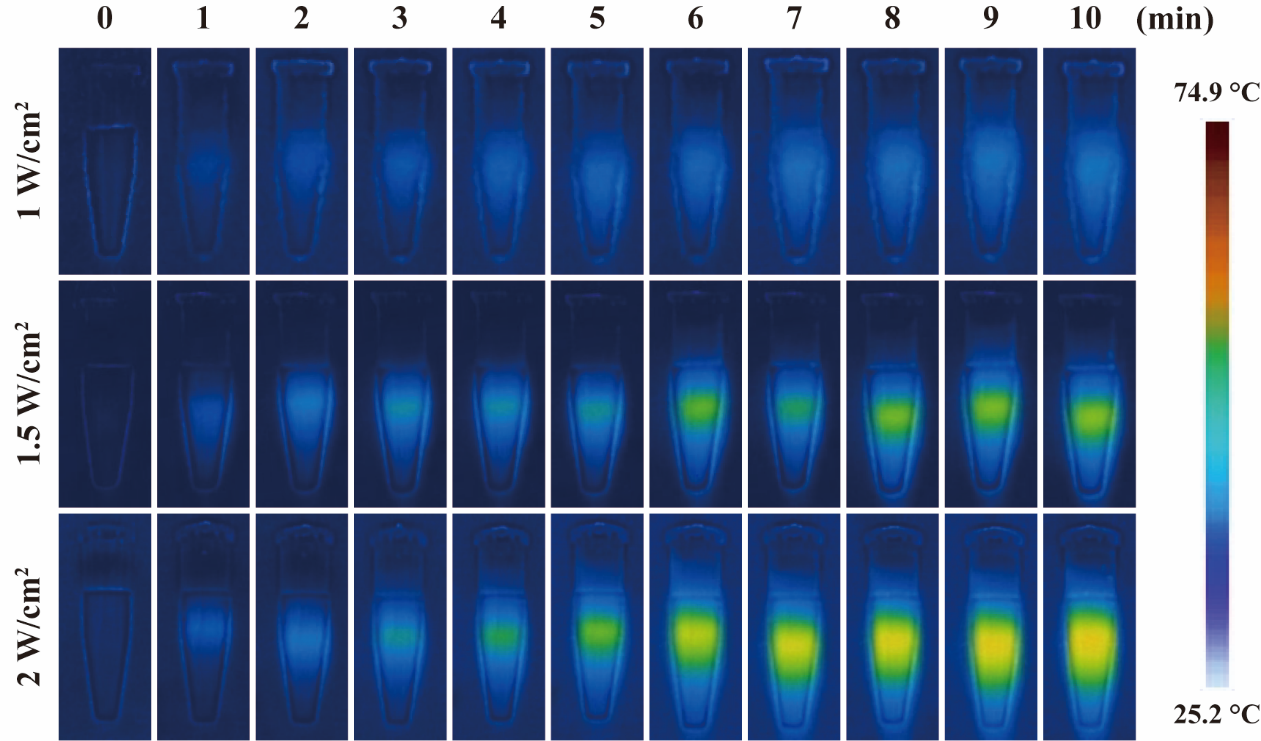
**

**Figure S7.** Photothermal images of 200 μg/mL CP under different NIR irradiation intensity (1, 1.5 and 2 W/cm^2^) versus time.


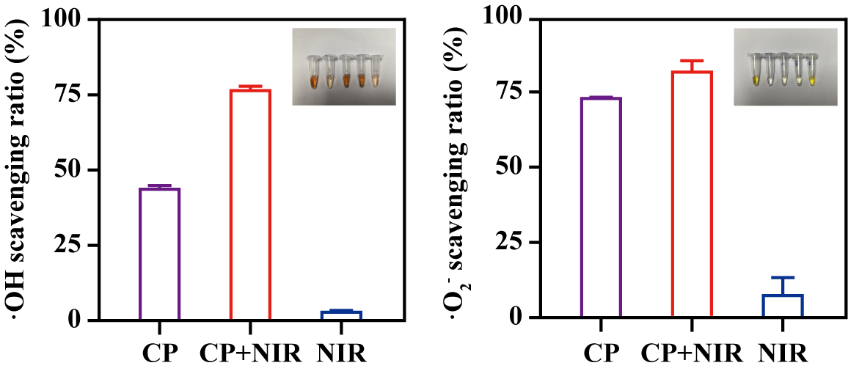


**Figure S8.** ROS scavenging capacity by ·OH (i) and ·O_2_^-^ (ii) testing kits: 100 μg/mL CP, NIR (2 W/cm^2^) and 100 μg/mL CP+NIR.

**
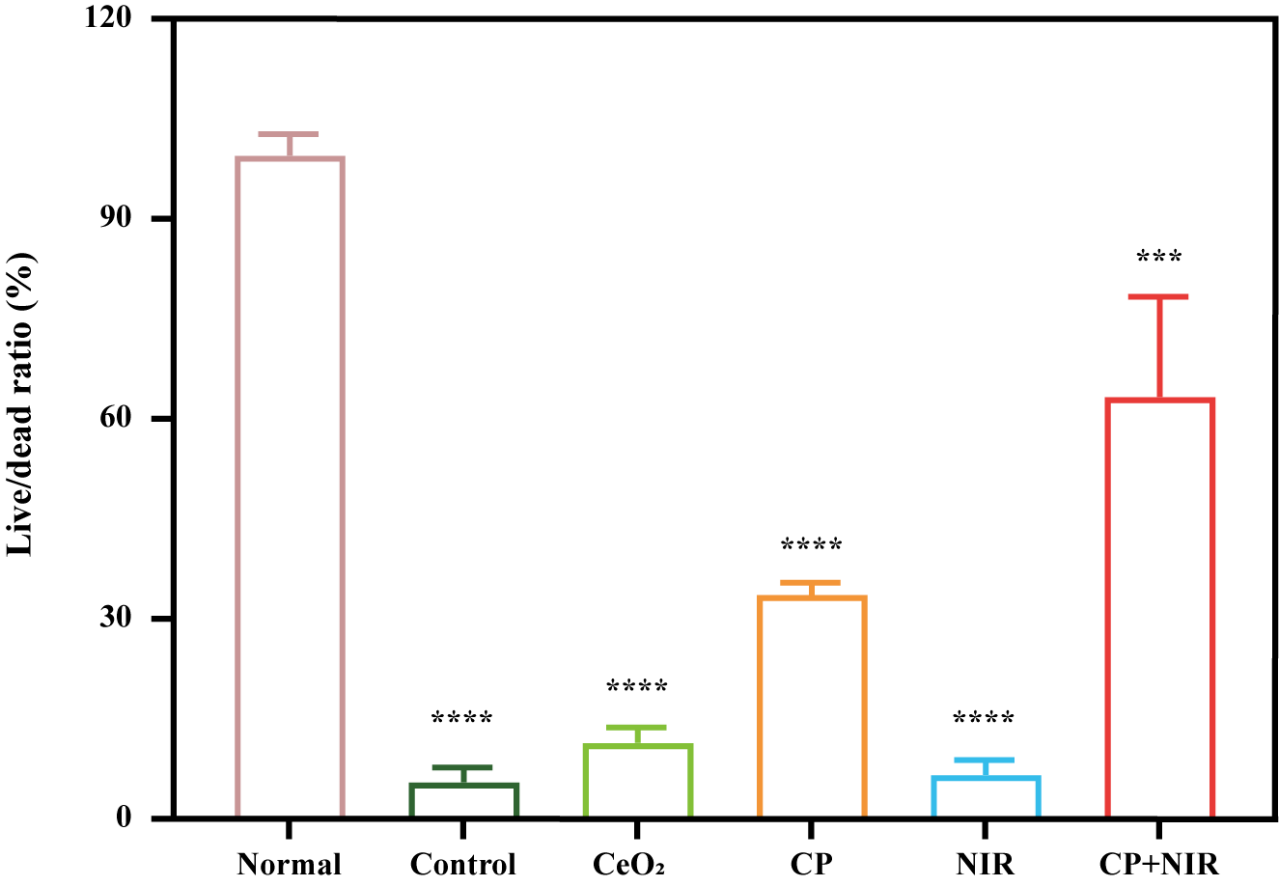
**

**Figure S9.** Quantified results of live/dead staining of treated cells. The corresponding groups were: cells without treatment (normal group), LPS induced cells followed by PBS treatment (control group), LPS induced cells followed by 100 μg/mL CeO_2_ treatment (CeO_2_), LPS induced cells followed by 100 μg/mL CP treatment (CP), LPS induced cells followed by NIR irradiation (2 W/cm^2^) (NIR), and LPS induced cells followed by 100 μg/mL CP combining with NIR irradiation (2 W/cm^2^) (CP+NIR). (“*” symbol compared with normal group, *p < 0.05, **p < 0.01, ***p < 0.001 and ****p < 0.0001)


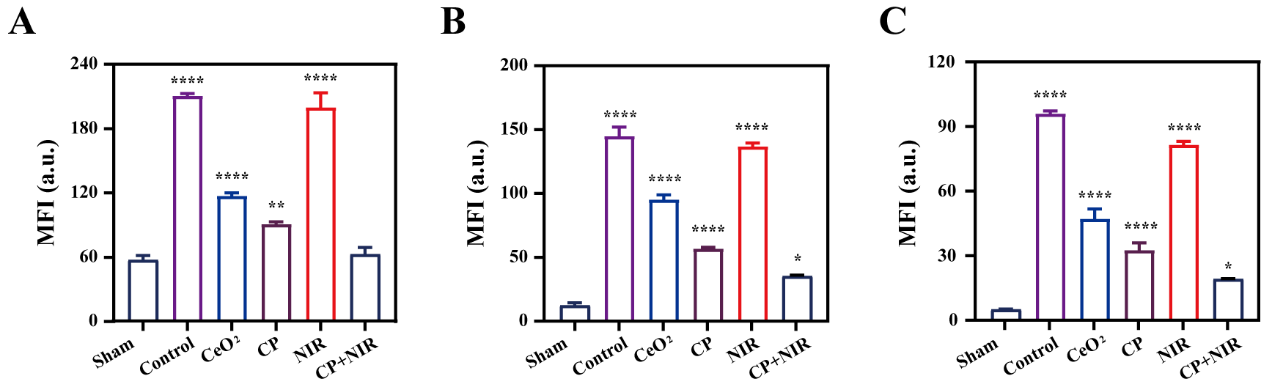


**Figure S10.** Quantified results of intracellular ROS levels of treated cells: HPF (A), DHE (B) and DCFA (C). The corresponding groups were: cells without treatment (normal group), LPS induced cells followed by PBS treatment (control group), LPS induced cells followed by 100 μg/mL CeO_2_ treatment (CeO_2_), LPS induced cells followed by 100 μg/mL CP treatment (CP), LPS induced cells followed by NIR irradiation (2 W/cm^2^) (NIR), and LPS induced cells followed by 100 μg/mL CP combining with NIR irradiation (2 W/cm^2^) (CP+NIR). (“*” symbol compared with normal group, *p < 0.05, **p < 0.01, ***p < 0.001 and ****p < 0.0001)

**
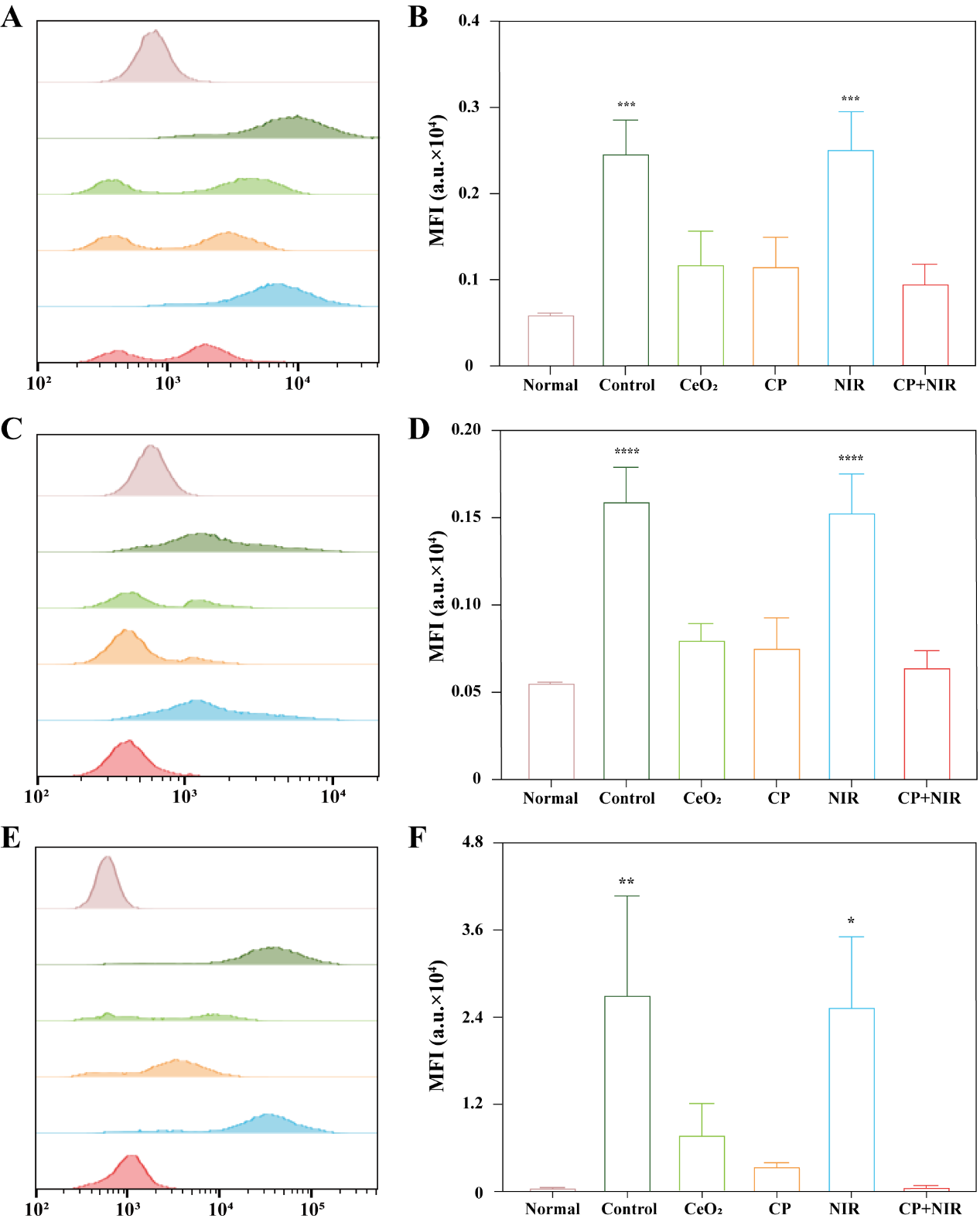
**

**Figure S11.** Flow cytometry results of intracellular ROS levels of treated cells, and the corresponding quantified results: HPF (A and B), DHE (C and D) and DCFA (E and F). The corresponding groups were: cells without treatment (normal group), LPS induced cells followed by PBS treatment (control group), LPS induced cells followed by 100 μg/mL CeO_2_ treatment (CeO_2_), LPS induced cells followed by 100 μg/mL CP treatment (CP), LPS induced cells followed by NIR irradiation (2 W/cm^2^) (NIR), and LPS induced cells followed by 100 μg/mL CP combining with NIR irradiation (2 W/cm^2^) (CP+NIR). (“*” symbol compared with normal group, *p < 0.05, **p < 0.01, ***p < 0.001 and ****p < 0.0001)

**
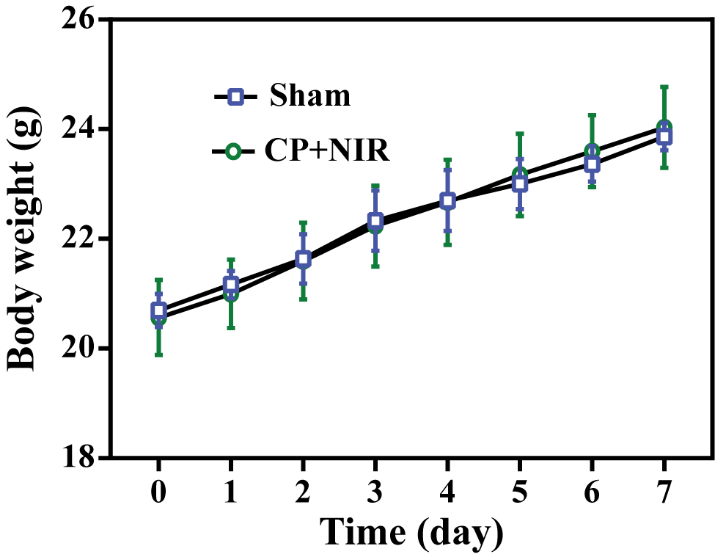
**

**Figure S12.** The body weight change of treated mice after 7 days. The corresponding groups were: mice with saline injection (sham), and mice with CP injection and NIR irradiation (2 W/cm^2^) (CP+NIR).

**
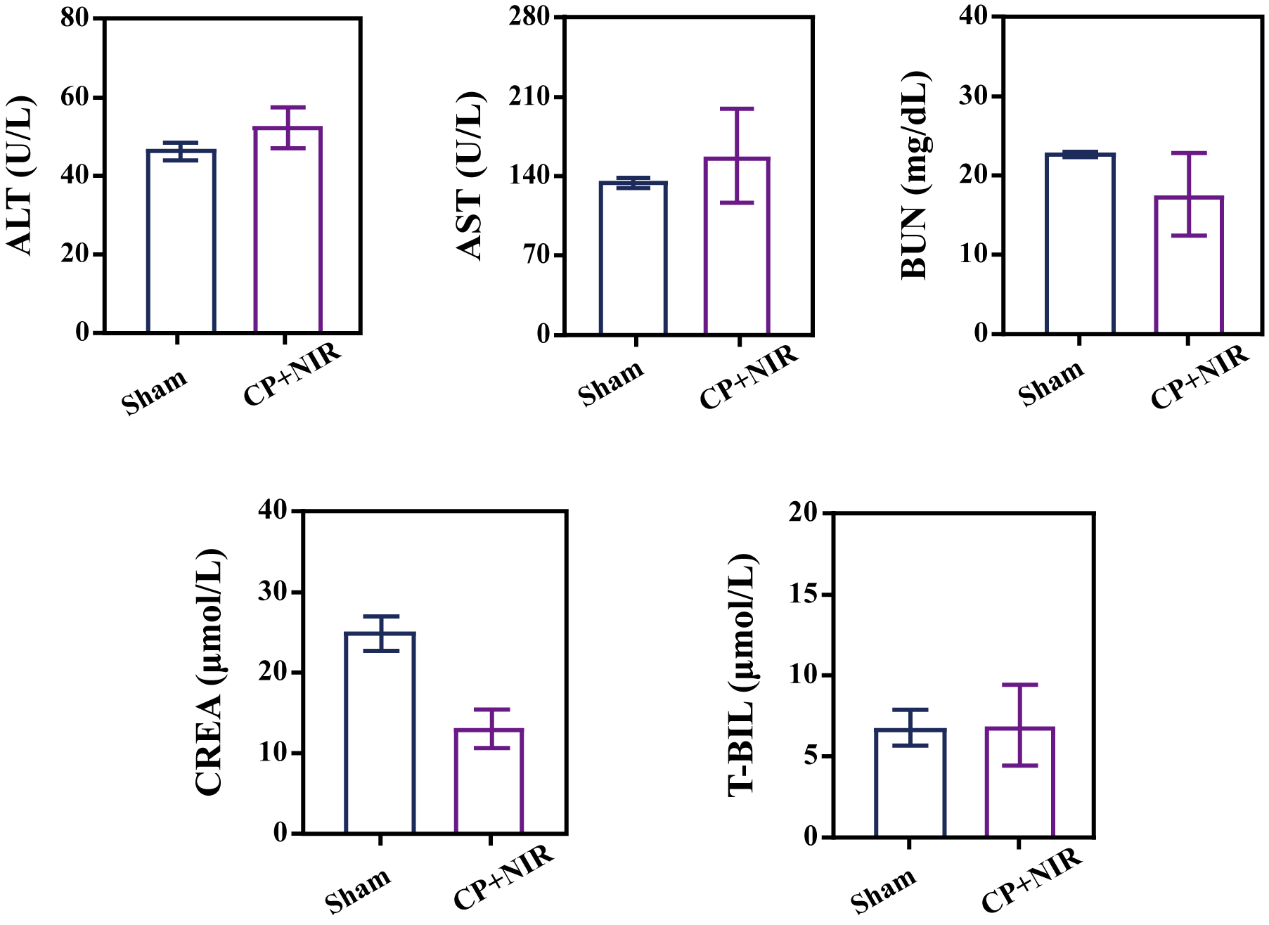
**

**Figure S13.** Blood indicators of treated mice after 7 days. The corresponding groups were: mice with saline injection (sham), and mice with CP injection and NIR irradiation (2 W/cm^2^) (CP+NIR).

**
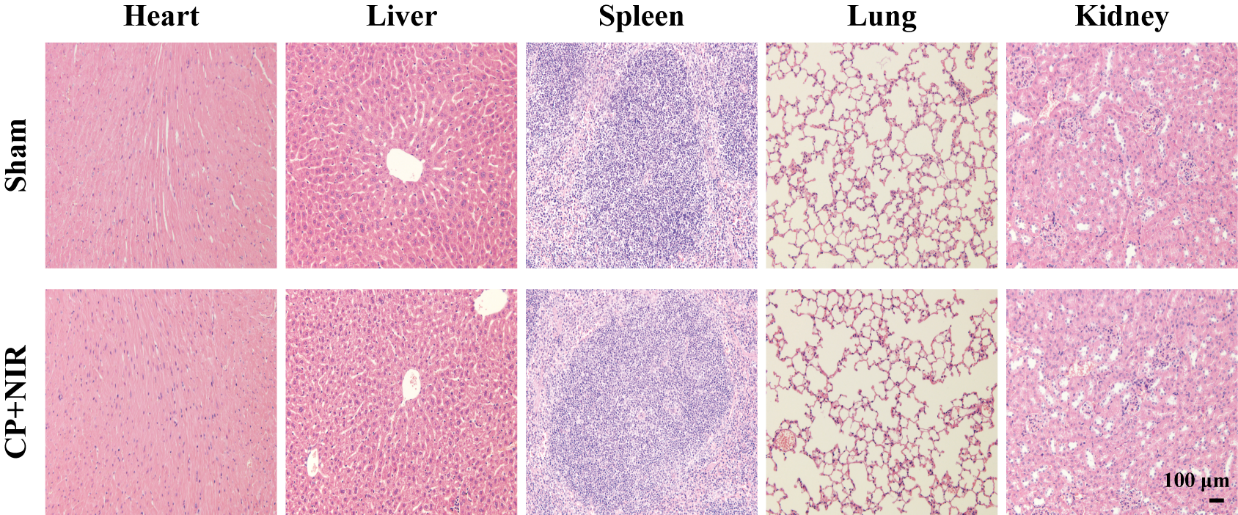
**

**Figure S14.** H&E staining images of major organs (heart, liver, spleen, lung and kidney) of treated mice after 7 days. The corresponding groups were: mice with saline injection (sham), and mice with CP injection and NIR irradiation (2 W/cm^2^) (CP+NIR).

**
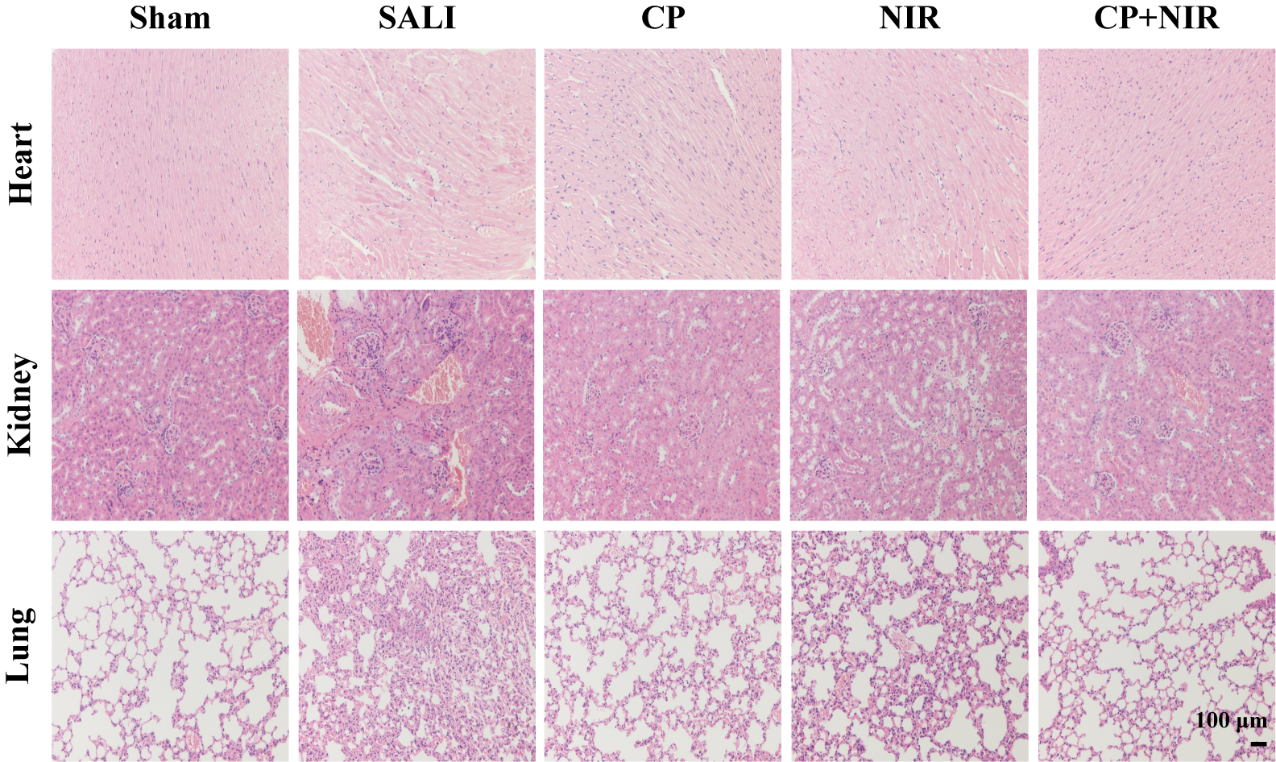
**

**Figure S15.** H&E staining images of other tissues (heart, kidney and lung) of treated mice. The corresponding groups were: mice without treatment (sham group), LPS induced mice with saline injection (SALI), LPS induced mice with CP injection (CP), LPS induced mice with NIR irradiation (2 W/cm^2^) (NIR), and LPS induced mice with CP injection and NIR irradiation (2 W/cm^2^) (CP+NIR).

**
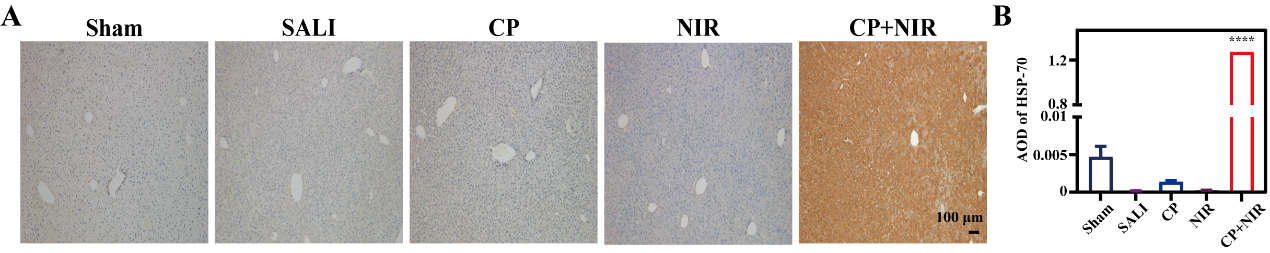
**

**Figure S16.** HSP70 expression levels of liver tissue of treated mice, and the corresponding quantified results. The corresponding groups were: mice without treatment (sham group), LPS induced mice with saline injection (SALI), LPS induced mice with CP injection (CP), LPS induced mice with NIR irradiation (2 W/cm^2^) (NIR), and LPS induced mice with CP injection and NIR irradiation (2 W/cm^2^) (CP+NIR). (“*” symbol compared with sham group, *p < 0.05, **p < 0.01, ***p < 0.001 and ****p < 0.0001)

**
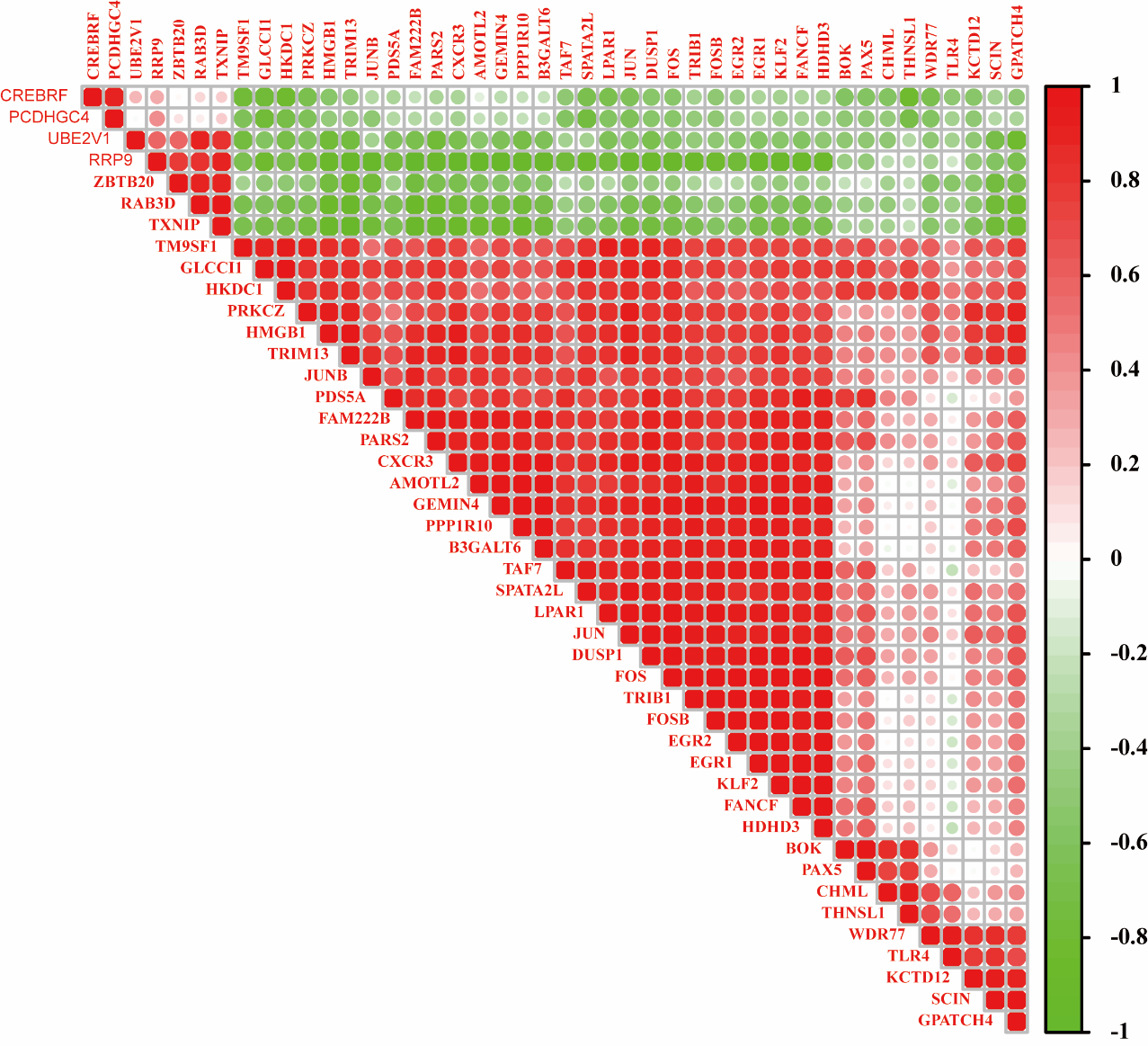
**

**Figure S17.** The complex expressional correlations among 44 autophagy related genes by Pearson correlation analysis. (P value < 0.05)

**
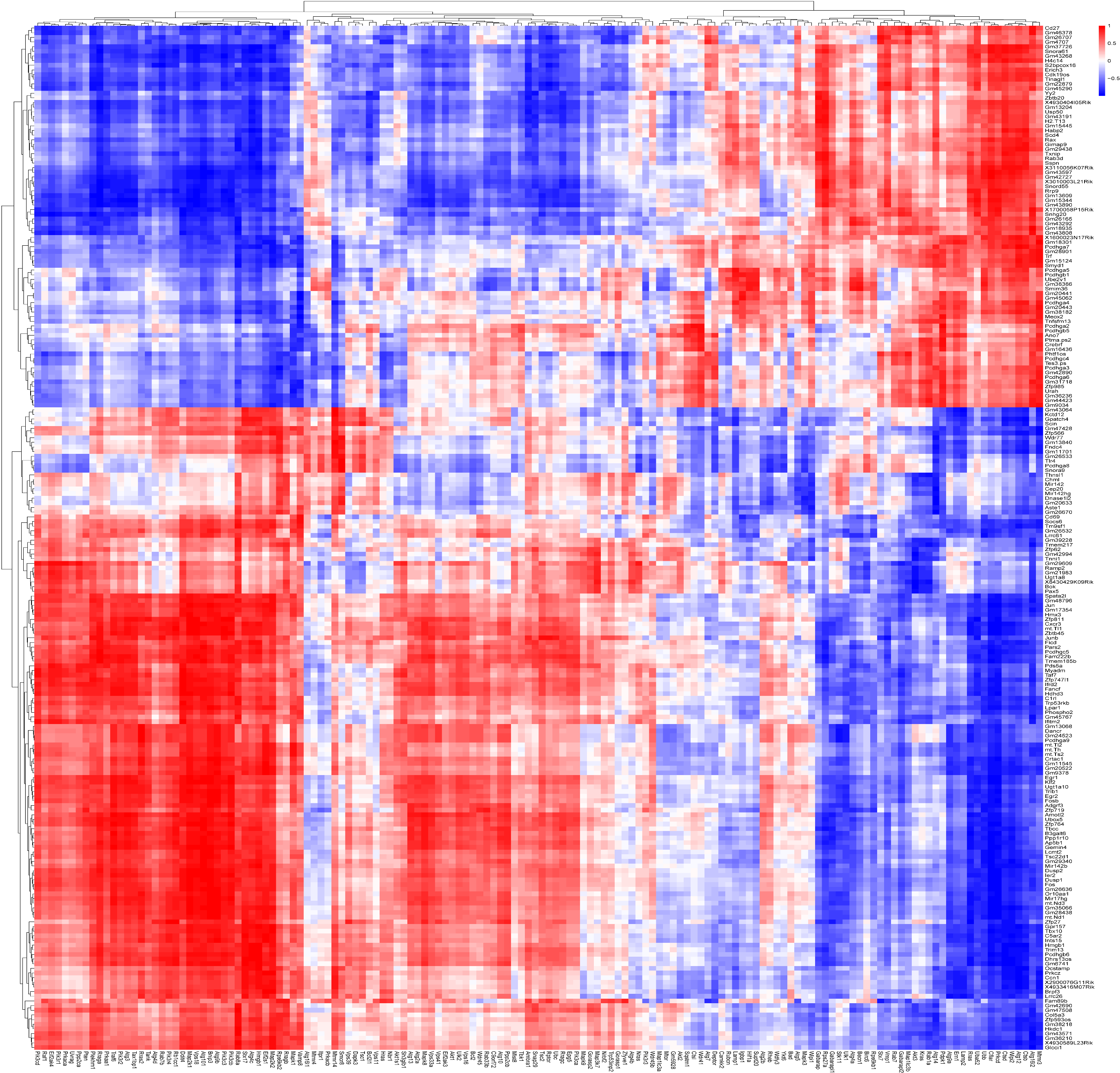
**

**Figure S18.** A correlation heatmap between DEGs and autophagy pathway genes.

**
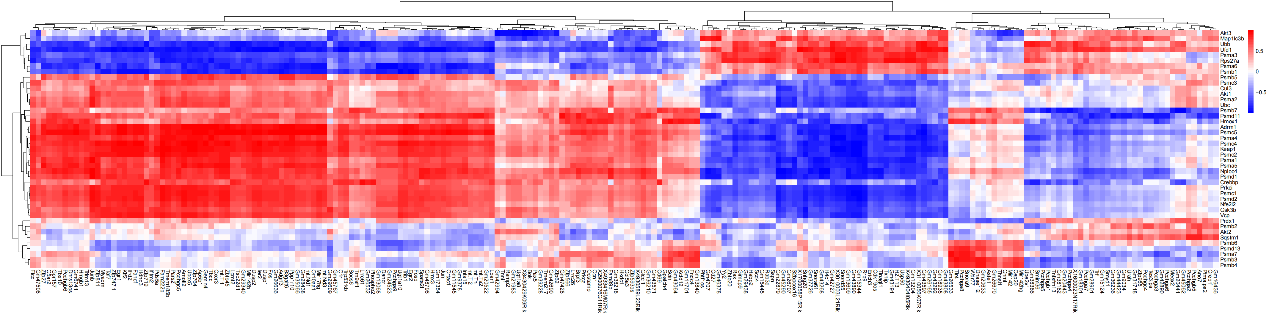
**

**Figure S19.** A correlation heatmap between DEGs and Keap1/Nrf-2/HO-1 pathway genes.

**
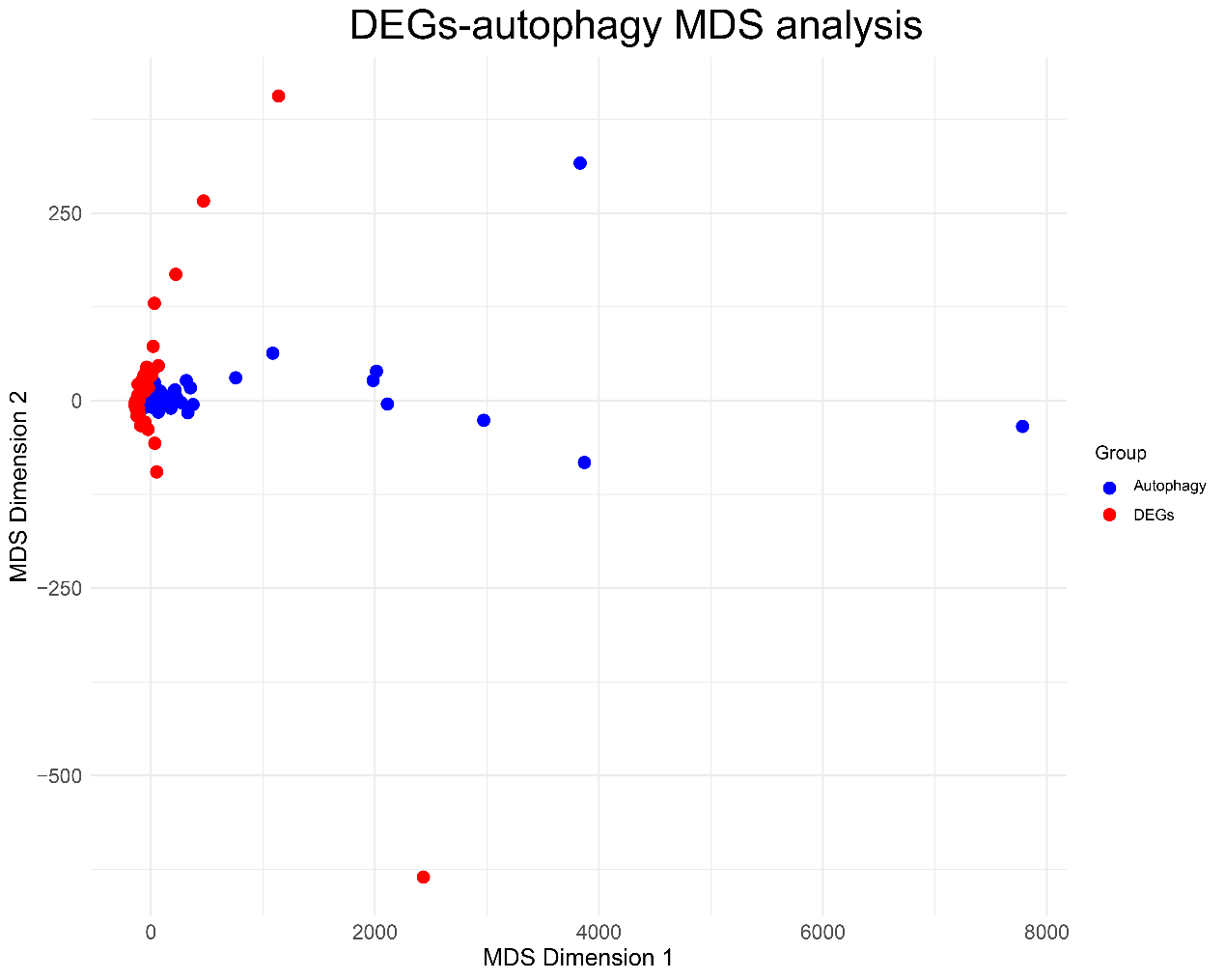
**

**Figure S20.** The MDS analysis of DEGs and autophagy pathway genes.

**
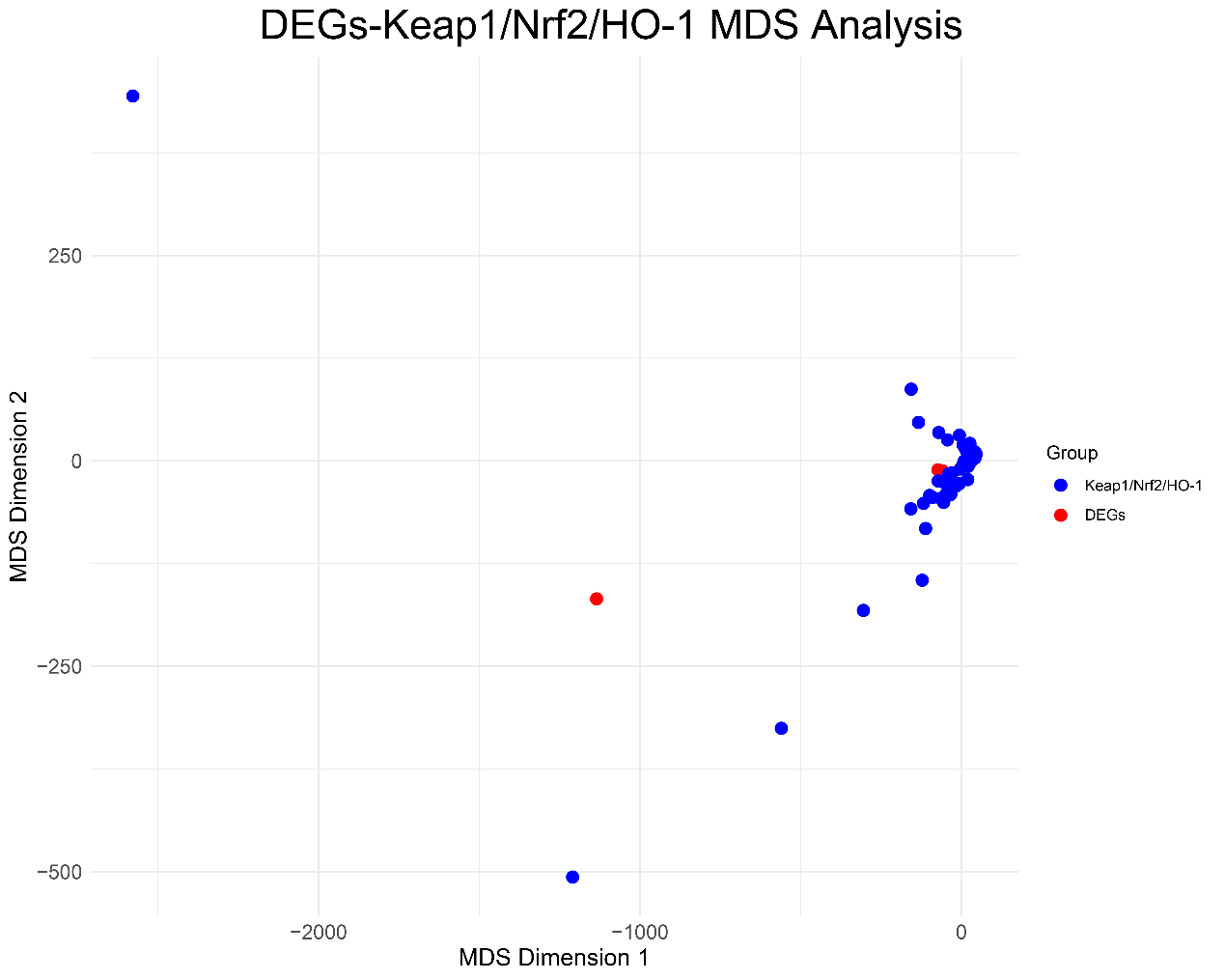
**

**Figure S21.** The MDS analysis of DEGs and Keap1/Nrf-2/HO-1 pathway genes.

**
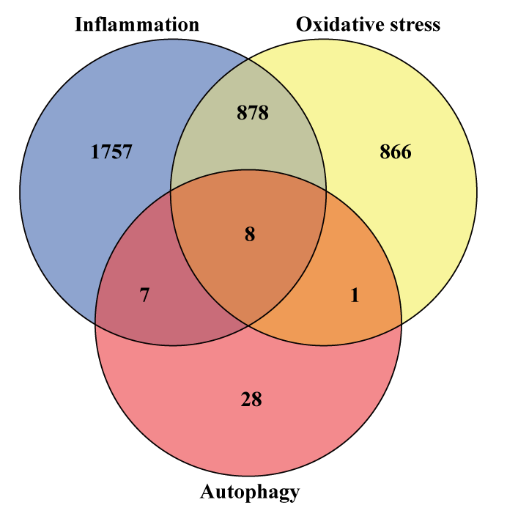
**

**Figure S22.** The Venn diagram of autophagy related DEGs, ROS clearance related genes, and anti-inflammation related genes.

**
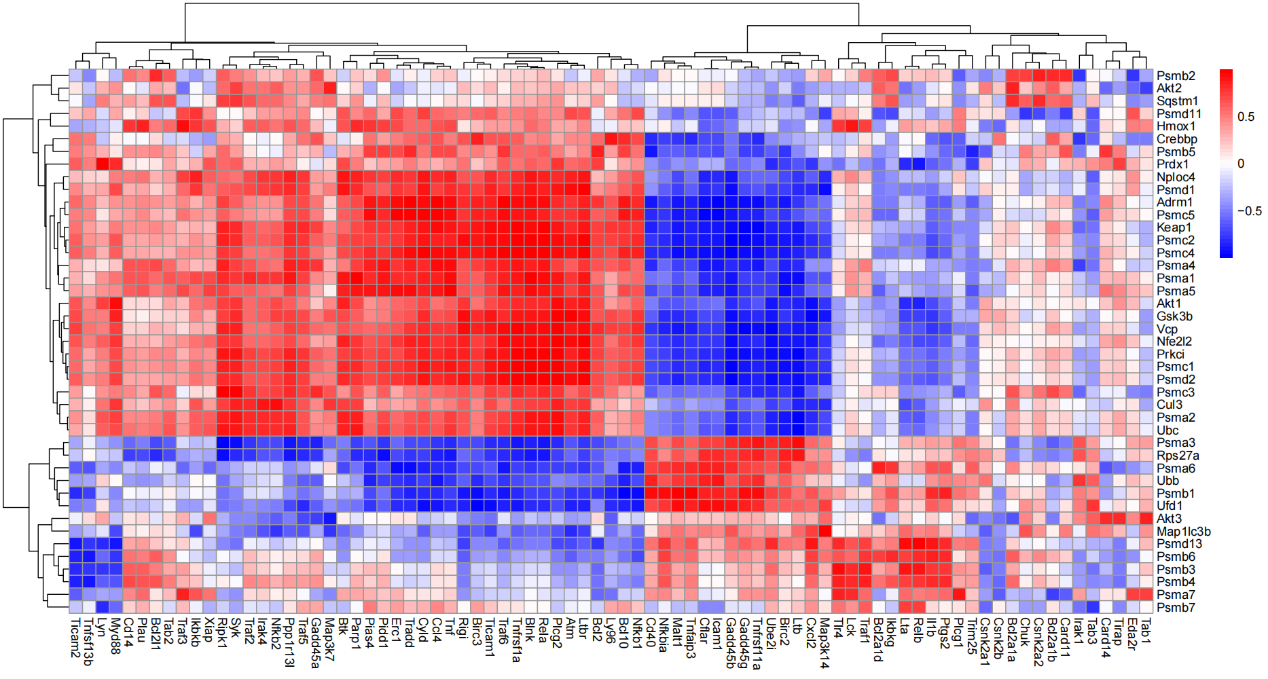
**

**Figure S23.** A correlation heatmap between NF-κB pathway genes and Keap1/Nrf-2/HO-1 pathway genes.

**
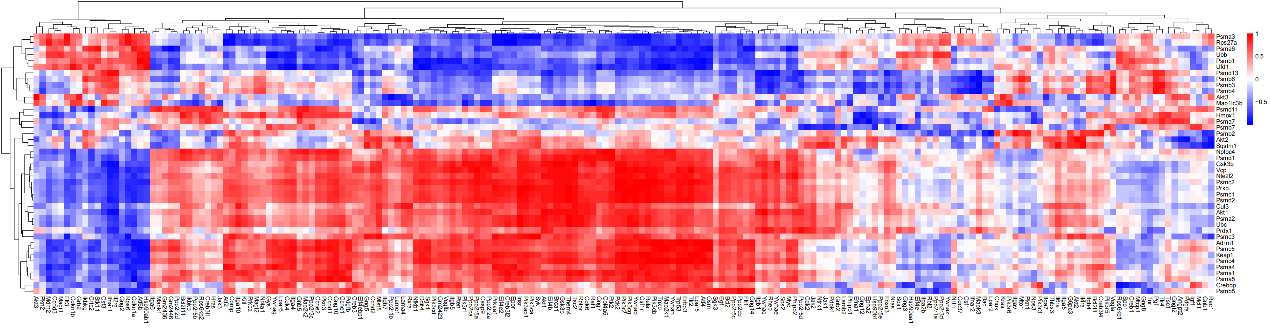
**

**Figure S24.** A correlation heatmap between PI3K-Akt pathway genes and Keap1/Nrf-2/HO-1 pathway genes.

**
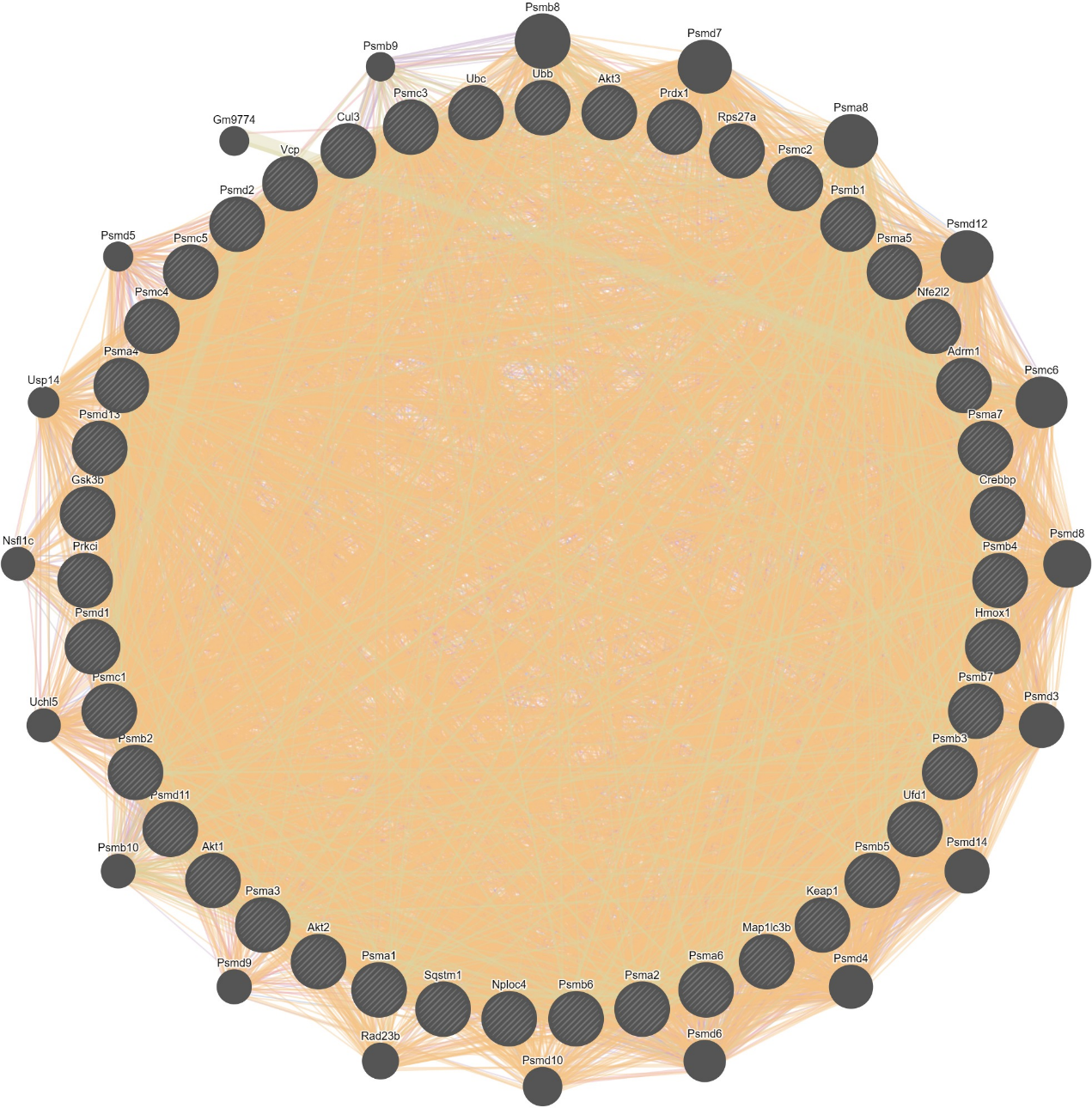
**

**Figure S25.** The interaction network diagram of Keap1/Nrf-2/HO-1 pathway related genes by the analysis of GeneMANIA. Nodes represented genes, and its size reflected the connectivity of genes in the network (more connections were equal to larger nodes). The inner circle represented pathways related genes, and the outer circle represented predicted related genes (orange yellow: prediction, purple: co-expression, red: physical interaction, blue: co-localization and indigo blue: pathway sharing).


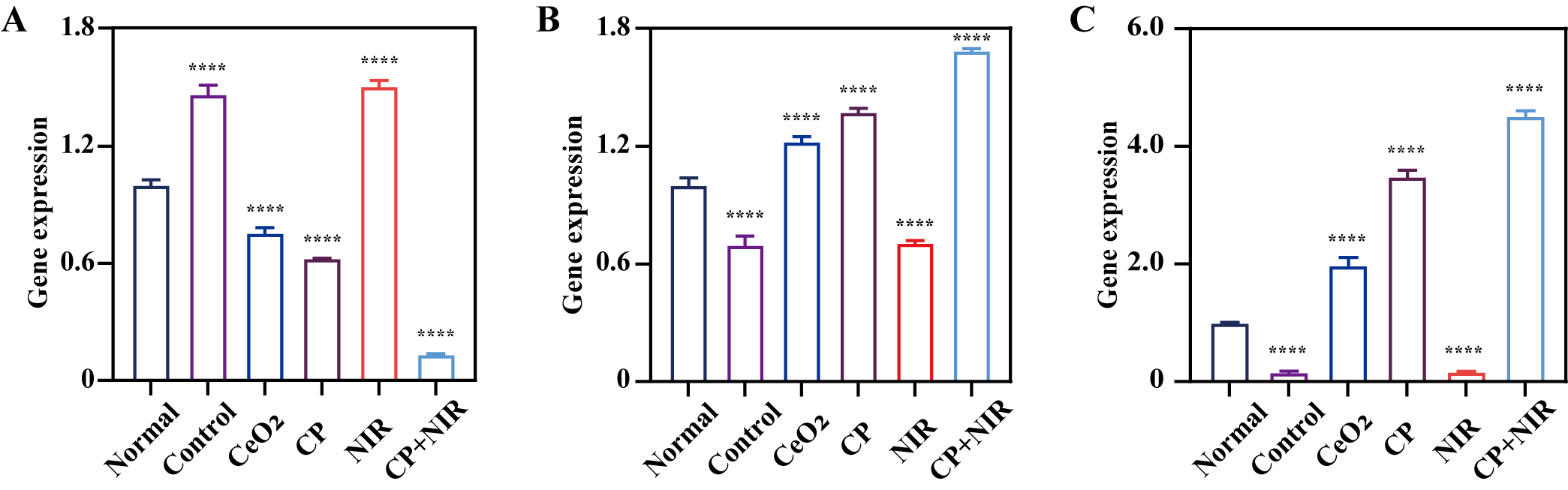


**Figure S26.** Relative genes expression levels of treated cells by RT-qPCR: Keap1 (A), GCLM (B) and NQO1 (C). The corresponding groups were: cells without treatment (normal group), LPS induced cells followed by PBS treatment (control group), LPS induced cells followed by 100 μg/mL CeO_2_ treatment (CeO_2_), LPS induced cells followed by 100 μg/mL CP treatment (CP), LPS induced cells followed by NIR irradiation (2 W/cm^2^) (NIR), and LPS induced cells followed by 100 μg/mL CP combining with NIR irradiation (2 W/cm^2^) (CP+NIR). (“*” symbol compared with normal group, *p < 0.05, **p < 0.01, ***p < 0.001 and ****p < 0.0001)

**
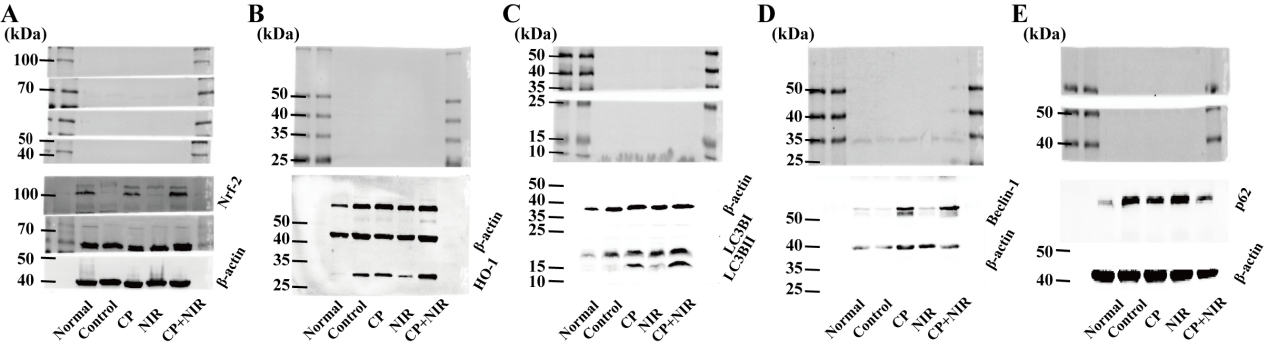
**

**Figure S27.** Original images of relative proteins expression levels of treated cells by WB: Nrf-2 (A), HO-1 (B), LC3BI and LC3BII (C), Beclin-1 (D) and p62 (E). The corresponding groups were: cells without treatment (normal group), LPS induced cells followed by PBS treatment (control group), LPS induced cells followed by 100 μg/mL CP treatment (CP), LPS induced cells followed by NIR irradiation (2 W/cm^2^) (NIR), and LPS induced cells followed by 100 μg/mL CP combining with NIR irradiation (2 W/cm^2^) (CP+NIR).


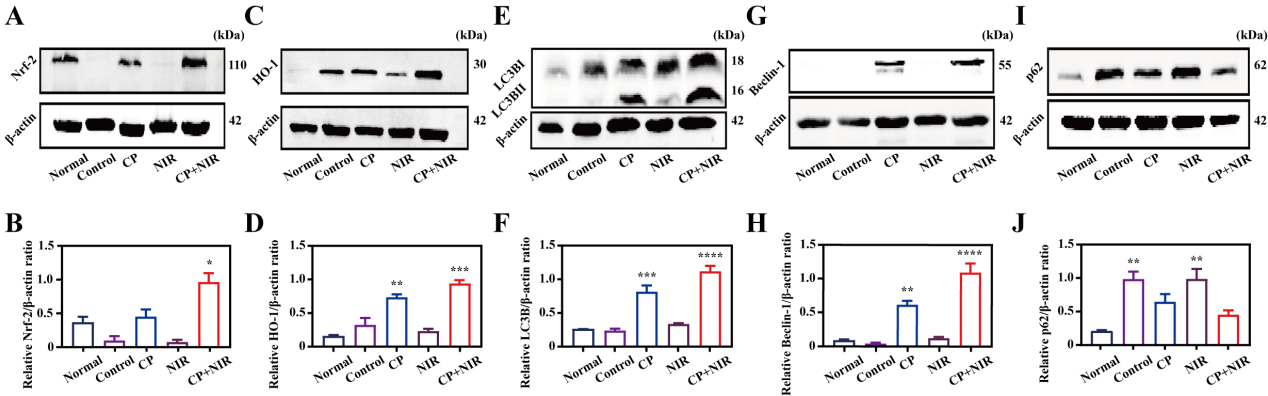


**Figure S28.** The relative proteins expression levels of treated cells by WB: Nrf-2 (A), HO-1 (C), LC3BI and LC3BII (E), Beclin-1 (G) and P62 (I), and the corresponding quantified results: relative Nrf-2/β-actin (B), HO-1/β-actin (D), LC3B/β-actin (F), Beclin-1/β-actin (H) and p62/β-actin (J) ratios. The corresponding groups were: cells without treatment (normal group), LPS induced cells followed by PBS treatment (control group), LPS induced cells followed by 100 μg/mL CP treatment (CP), LPS induced cells followed by NIR irradiation (2 W/cm^2^) (NIR), and LPS induced cells followed by 100 μg/mL CP combining with NIR irradiation (2 W/cm^2^) (CP+NIR). (“*” symbol compared with normal group, *p < 0.05, **p < 0.01, ***p < 0.001 and ****p < 0.0001)


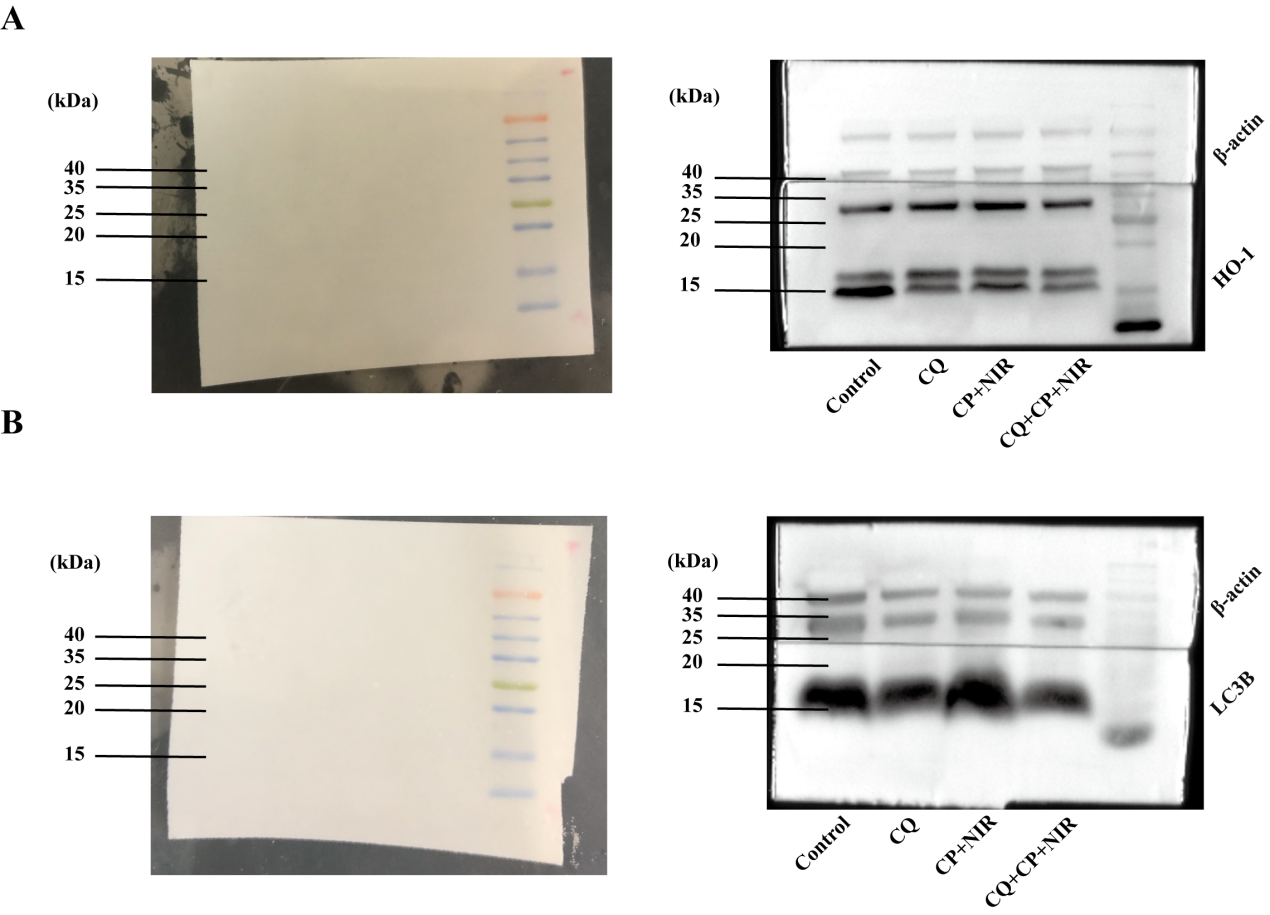


**Figure S29.** Original images of relative proteins expression levels of treated cells by WB: HO-1 (A) and LC3B (B). The corresponding groups were: LPS induced cells followed by PBS treatment (control group), LPS induced cells followed by CQ treatment (CQ), LPS induced cells followed by 100 μg/mL CP combining with NIR irradiation (2 W/cm^2^) (CP+NIR), and LPS induced cells followed by CQ treatment, and then 100 μg/mL CP combining with NIR irradiation (2 W/cm^2^) (CQ+CP+NIR).


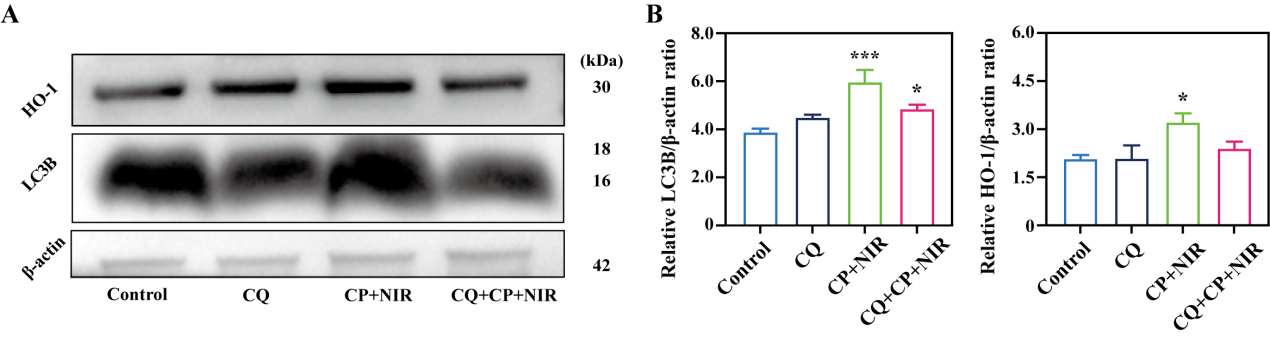


**Figure S30.** A) The relative proteins expression levels of treated cells by WB: HO-1 and LC3B, and the corresponding quantified results: relative LC3B/β-actin and HO-1/β-actin ratios (B). The corresponding groups were: LPS induced cells followed by PBS treatment (control group), LPS induced cells followed by CQ treatment (CQ), LPS induced cells followed by 100 μg/mL CP combining with NIR irradiation (2 W/cm^2^) (CP+NIR), and LPS induced cells followed by CQ treatment, and then 100 μg/mL CP combining with NIR irradiation (2 W/cm^2^) (CQ+CP+NIR). (“*” symbol compared with control group, *p < 0.05, **p < 0.01, ***p < 0.001 and ****p < 0.0001)

**
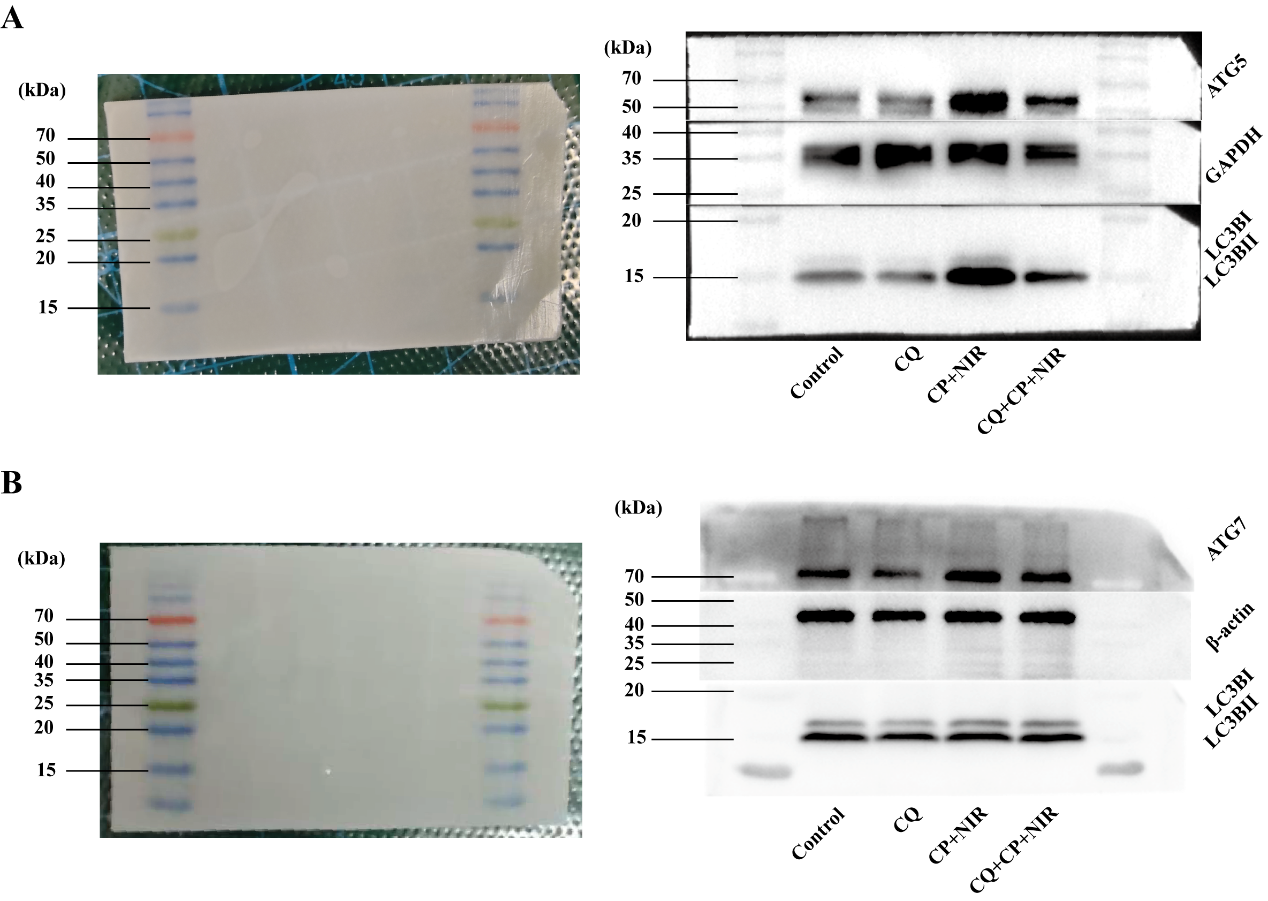
**

**Figure S31.** Original images of relative proteins expression levels of treated cells by WB: ATG5, LC3BI and LC3BII (A), and ATG7, LC3BI and LC3BII (B). The corresponding groups were: LPS induced cells followed by PBS treatment (control group), LPS induced cells followed by CQ treatment (CQ), LPS induced cells followed by 100 μg/mL CP combining with NIR irradiation (2 W/cm^2^) (CP+NIR), and LPS induced cells followed by CQ treatment, and then 100 μg/mL CP combining with NIR irradiation (2 W/cm^2^) (CQ+CP+NIR).


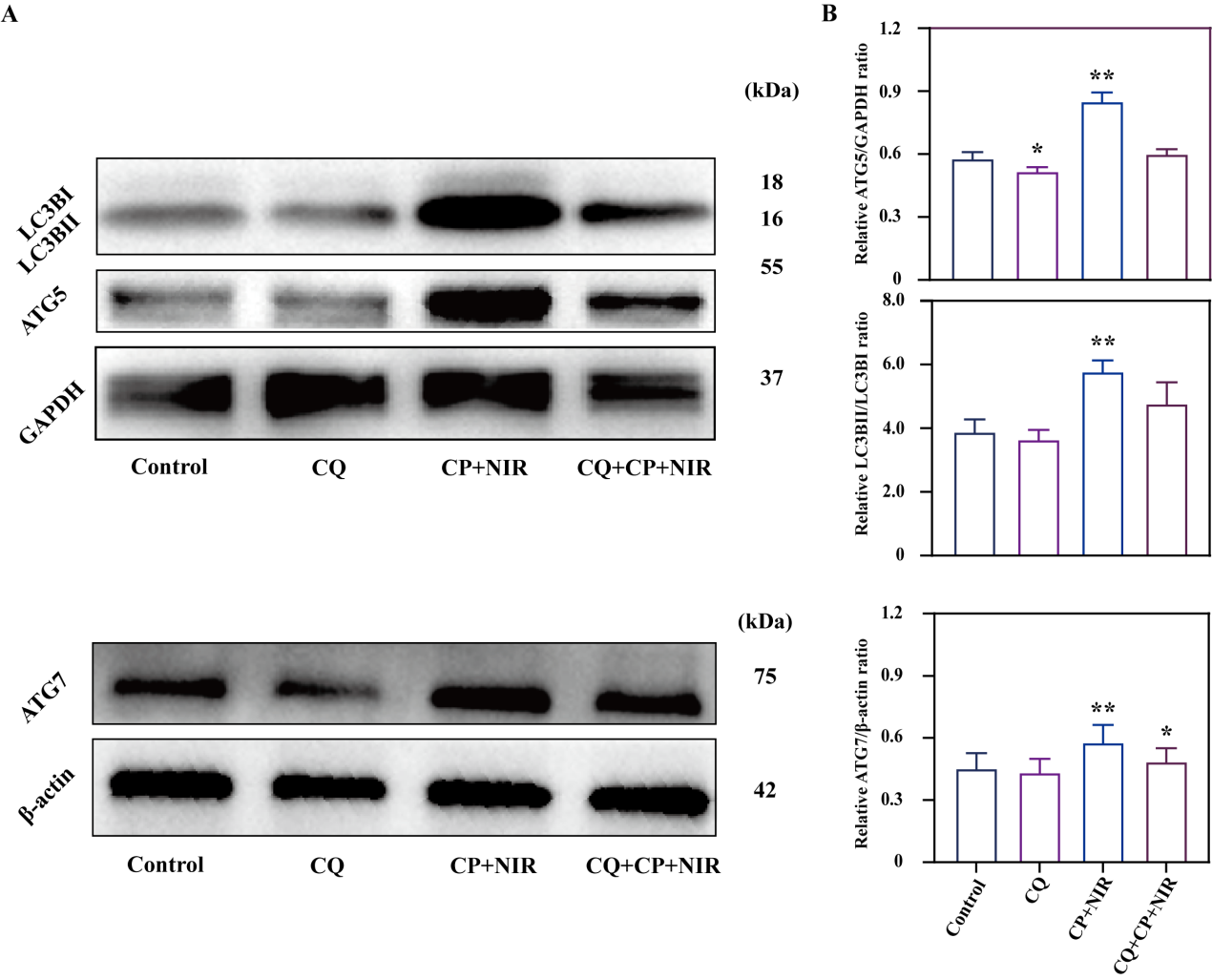


**Figure S32.** A) The relative proteins expression levels of treated cells by WB: ATG5, LC3BI, LC3BII, and ATG7, and the corresponding quantified results: relative ATG5/GAPDH, LC3BI/LC3BII and ATG7/β-actin ratios (B). The corresponding groups were: LPS induced cells followed by PBS treatment (control group), LPS induced cells followed by CQ treatment (CQ), LPS induced cells followed by 100 μg/mL CP combining with NIR irradiation (2 W/cm^2^) (CP+NIR), and LPS induced cells followed by CQ treatment, and then 100 μg/mL CP combining with NIR irradiation (2 W/cm^2^) (CQ+CP+NIR). (“*” symbol compared with control group, *p < 0.05, **p < 0.01, ***p < 0.001 and ****p < 0.0001)


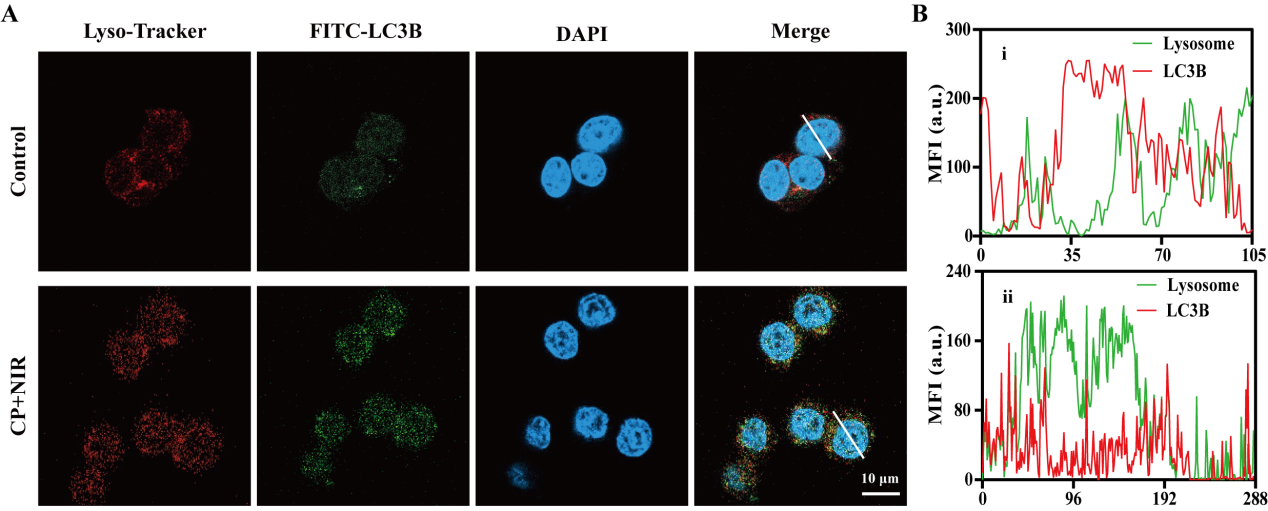


**Figure S33.** A) Co-immunostaining images of treated cells (Lysosome: red, LC3B: green and DAPI: blue) by confocal microscope, and the corresponding co-localization results (B): control group (i) and CP+NIR (ii). The corresponding groups were: LPS induced cells followed by PBS treatment (control group), and LPS induced cells followed by 100 μg/mL CP combining with NIR irradiation (2 W/cm^2^) (CP+NIR).

**
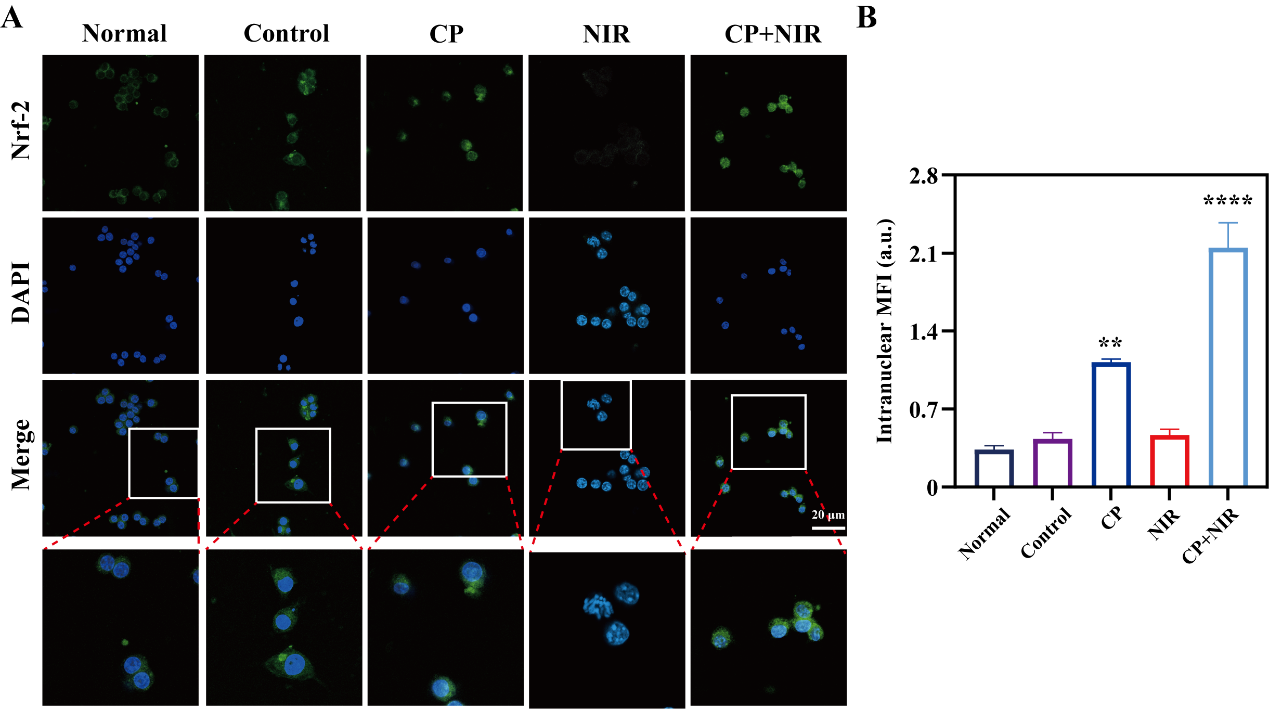
**

**Figure S34.** A) Co-immunostaining images of treated cells (Nrf-2: green and DAPI: blue) by confocal microscope, and the corresponding intranuclear MFI (B). The corresponding groups were: cells without treatment (normal group), LPS induced cells followed by PBS treatment (control group), LPS induced cells followed by 100 μg/mL CP treatment (CP), LPS induced cells followed by NIR irradiation (2 W/cm^2^) (NIR), and LPS induced cells followed by 100 μg/mL CP combining with NIR irradiation (2 W/cm^2^) (CP+NIR). (“*” symbol compared with normal group, *p < 0.05, **p < 0.01, ***p < 0.001 and ****p < 0.0001)

**
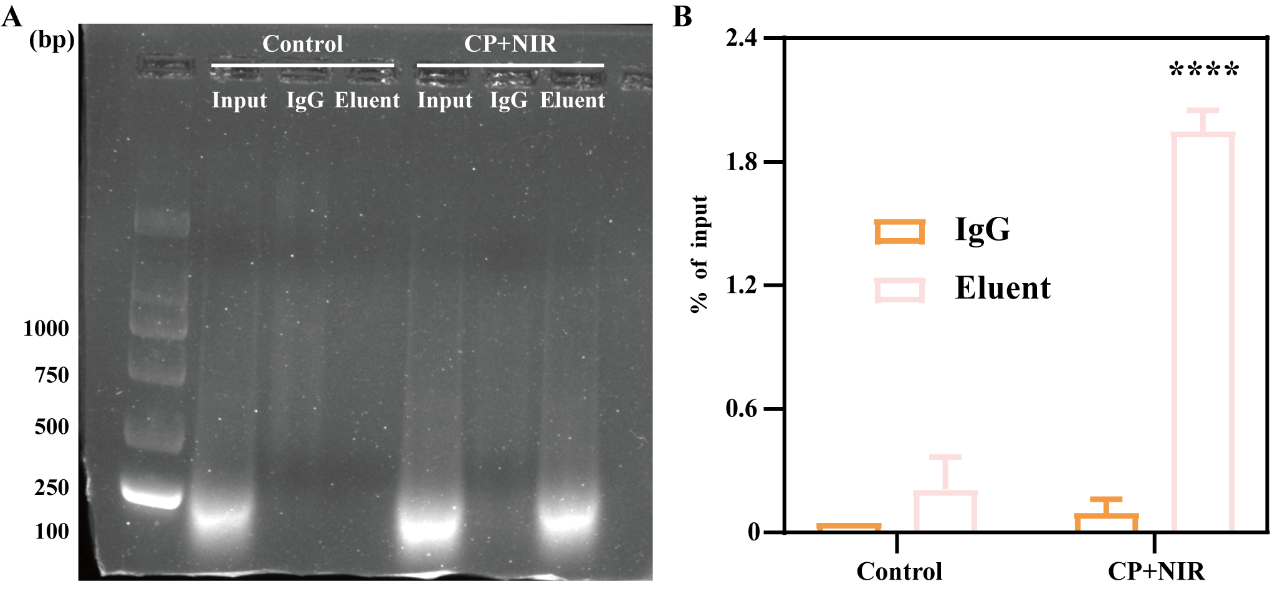
**

**Figure S35.** A) Nrf-2 gene content of different samples of treated cells after ChIP by DNA gel electrophoresis, and the relative Nrf-2 gene expression levels of different samples of treated cells after ChIP by RT-qPCR. The corresponding groups were: LPS induced cells followed by PBS treatment (control group) and LPS induced cells followed by 100 μg/mL CP combining with NIR irradiation (2 W/cm^2^) (CP+NIR). (“*” symbol compared with control group, *p < 0.05, **p < 0.01, ***p < 0.001 and ****p < 0.0001)

**
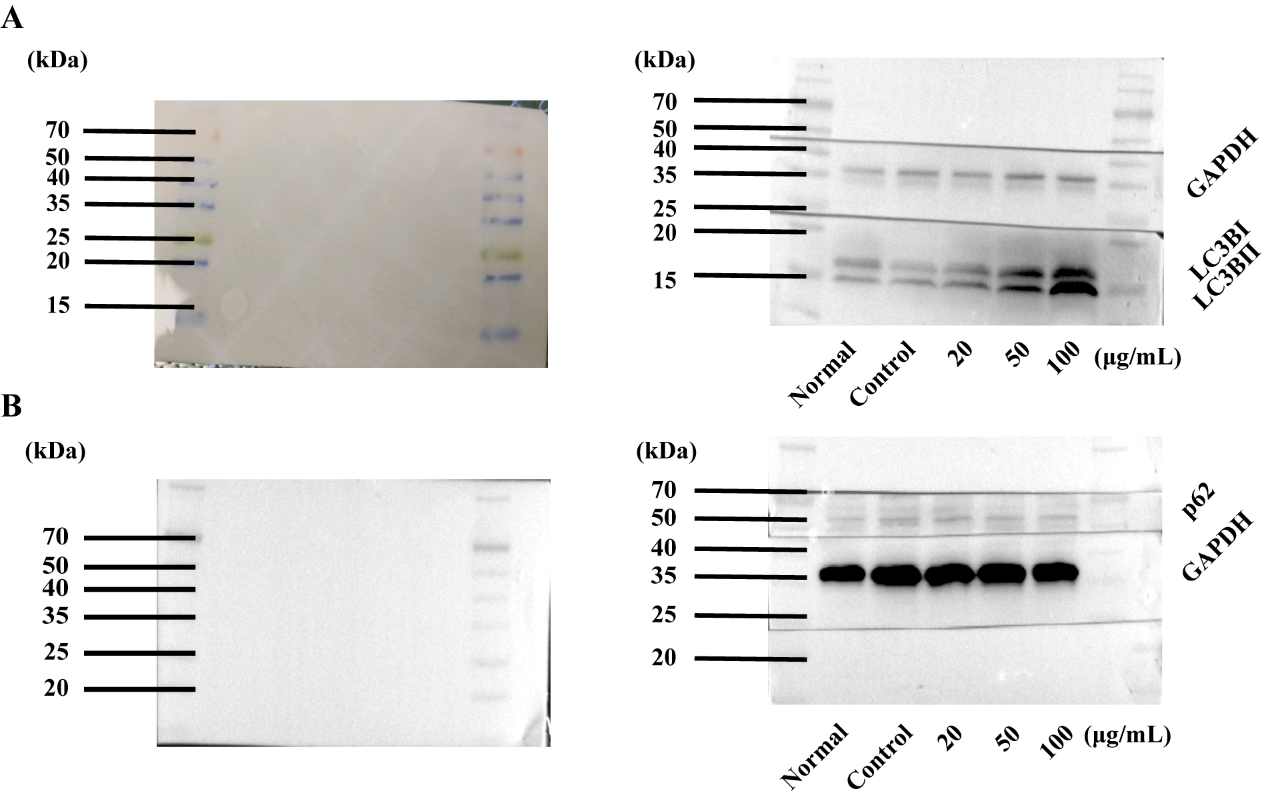
Figure S36.** Original images of relative proteins expression levels of treated cells by WB: LC3BI and LC3BII (A), and p62 (B). The corresponding groups were: cells without treatment (normal group), LPS induced cells followed by PBS treatment (control group), and LPS induced cells followed by 20, 50 and 100 μg/mL CP combining with NIR irradiation (2 W/cm^2^) overnight.


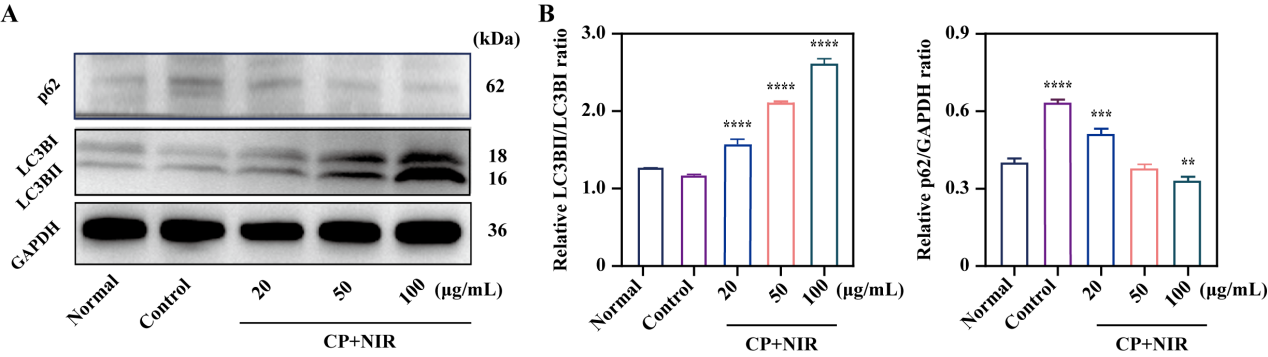


**Figure S37.** A) The relative proteins expression levels of treated cells by WB: p62, LC3BI and LC3BII, and the corresponding quantified results: relative LC3BI/LC3BII and p62/GAPDH ratios (B). The corresponding groups were: cells without treatment (normal group), LPS induced cells followed by PBS treatment (control group), and LPS induced cells followed by 20, 50 and 100 μg/mL CP combining with NIR irradiation (2 W/cm^2^) overnight. (“*” symbol compared with normal group, *p < 0.05, **p < 0.01, ***p < 0.001 and ****p < 0.0001)

**
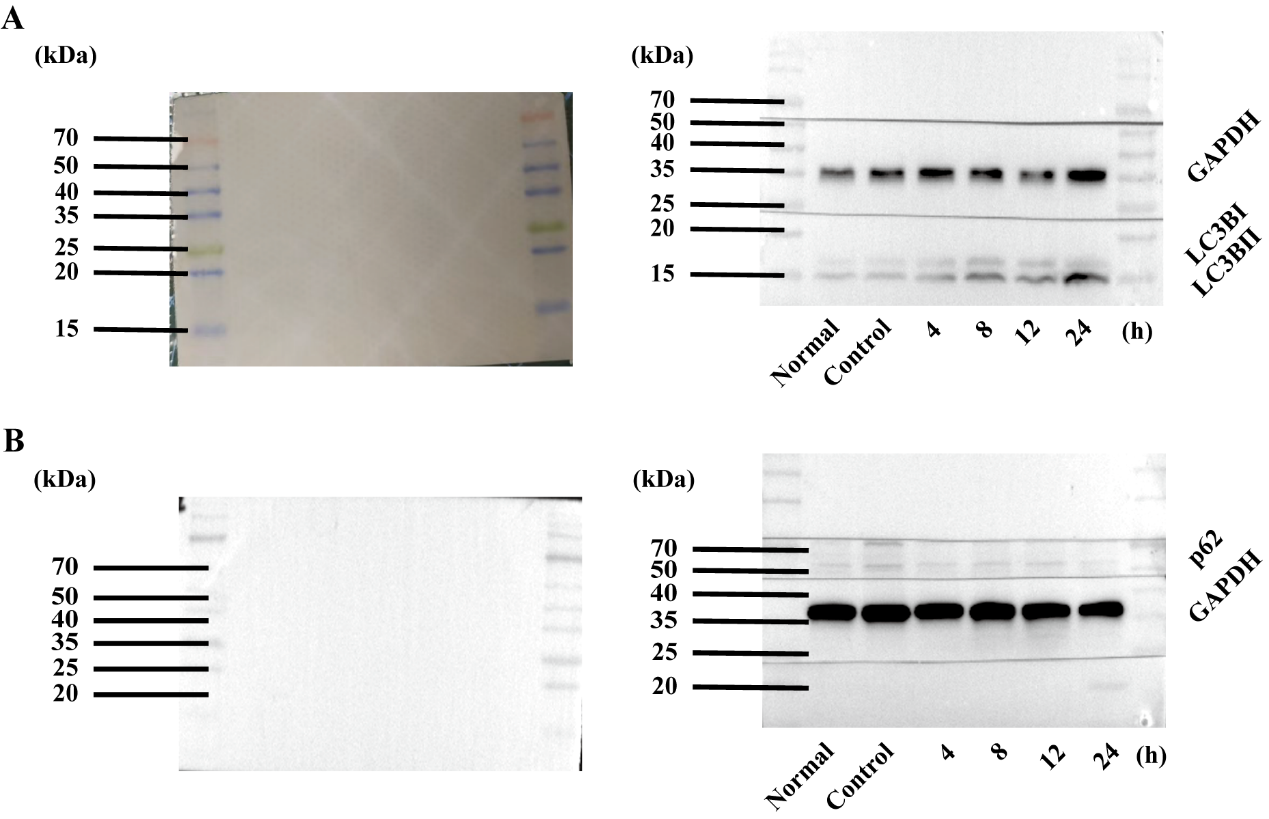
**

**Figure S38.** Original images of relative proteins expression levels of treated cells by WB: LC3BI and LC3BII (A), and p62 (B). The corresponding groups were: cells without treatment (normal group), LPS induced cells followed by PBS treatment (control group), and LPS induced cells followed by 100 μg/mL CP combining with NIR irradiation (2 W/cm^2^) for 4, 8, 12 and 24 h.


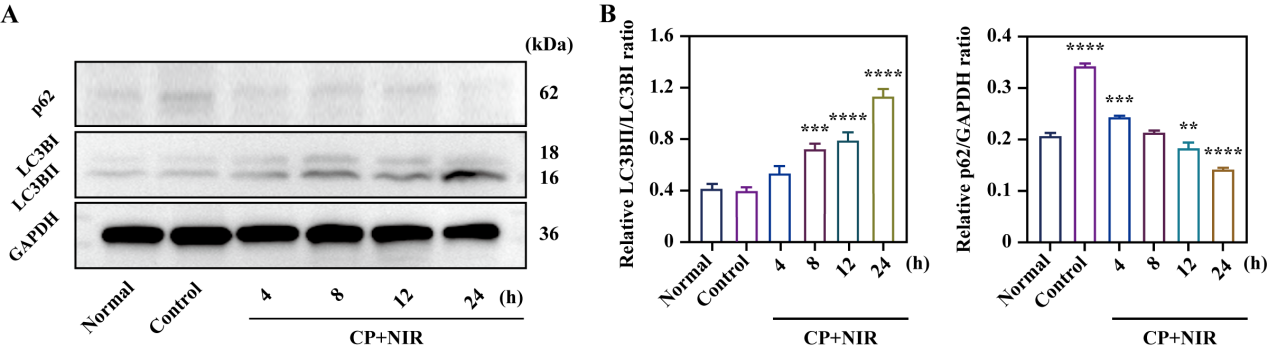


**Figure S39.** A) The relative proteins expression levels of treated cells by WB: p62, LC3BI and LC3BII, and the corresponding quantified results: relative LC3BI/LC3BII and p62/GAPDH ratios (B). The corresponding groups were: cells without treatment (normal group), LPS induced cells followed by PBS treatment (control group), and LPS induced cells followed by 100 μg/mL CP combining with NIR irradiation (2 W/cm^2^) for 4, 8, 12 and 24 h. (“*” symbol compared with normal group, *p < 0.05, **p < 0.01, ***p < 0.001 and ****p < 0.0001)


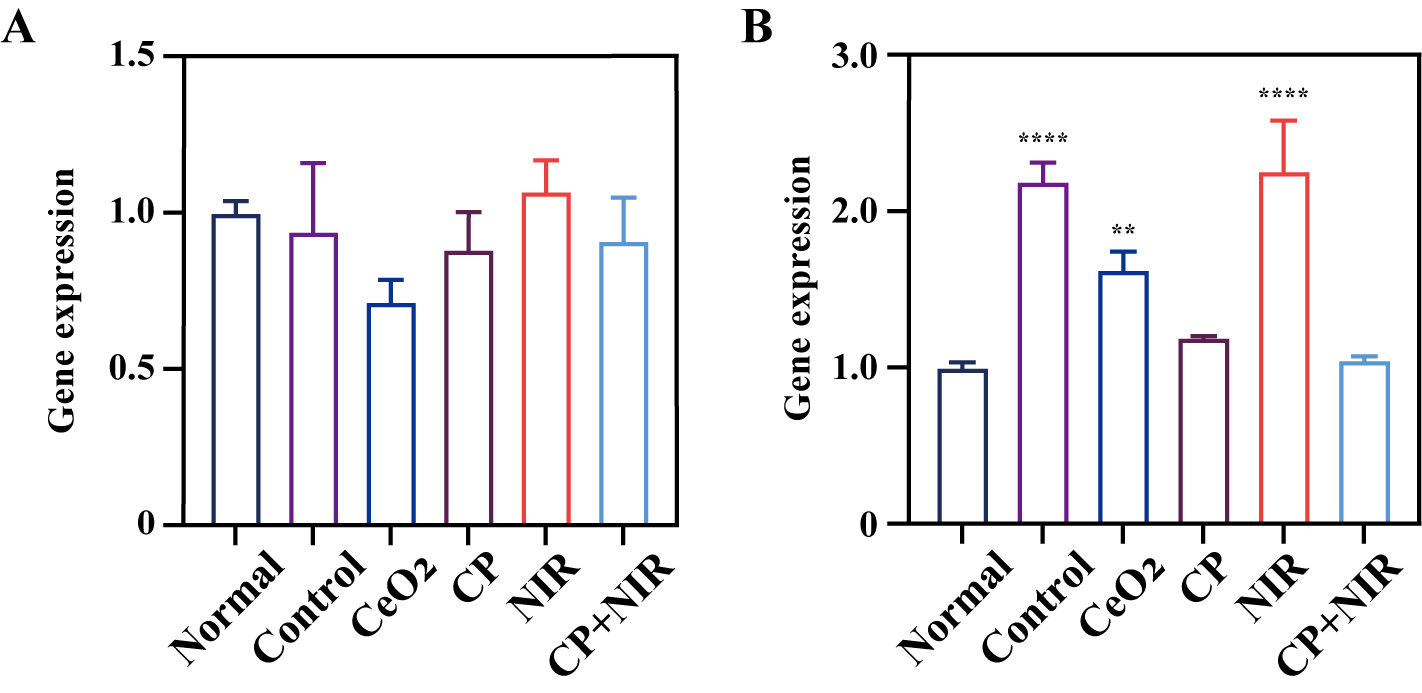


**Figure S40.** Relative genes expression levels of treated cells by RT-qPCR: p65 (A) and COX-2 (B). The corresponding groups were: cells without treatment (normal group), LPS induced cells followed by PBS treatment (control group), LPS induced cells followed by 100 μg/mL CeO_2_ treatment (CeO_2_), LPS induced cells followed by 100 μg/mL CP treatment (CP), LPS induced cells followed by NIR irradiation (2 W/cm^2^) (NIR), and LPS induced cells followed by 100 μg/mL CP combining with NIR irradiation (2 W/cm^2^) (CP+NIR). (“*” symbol compared with normal group, *p < 0.05, **p < 0.01, ***p < 0.001 and ****p < 0.0001)


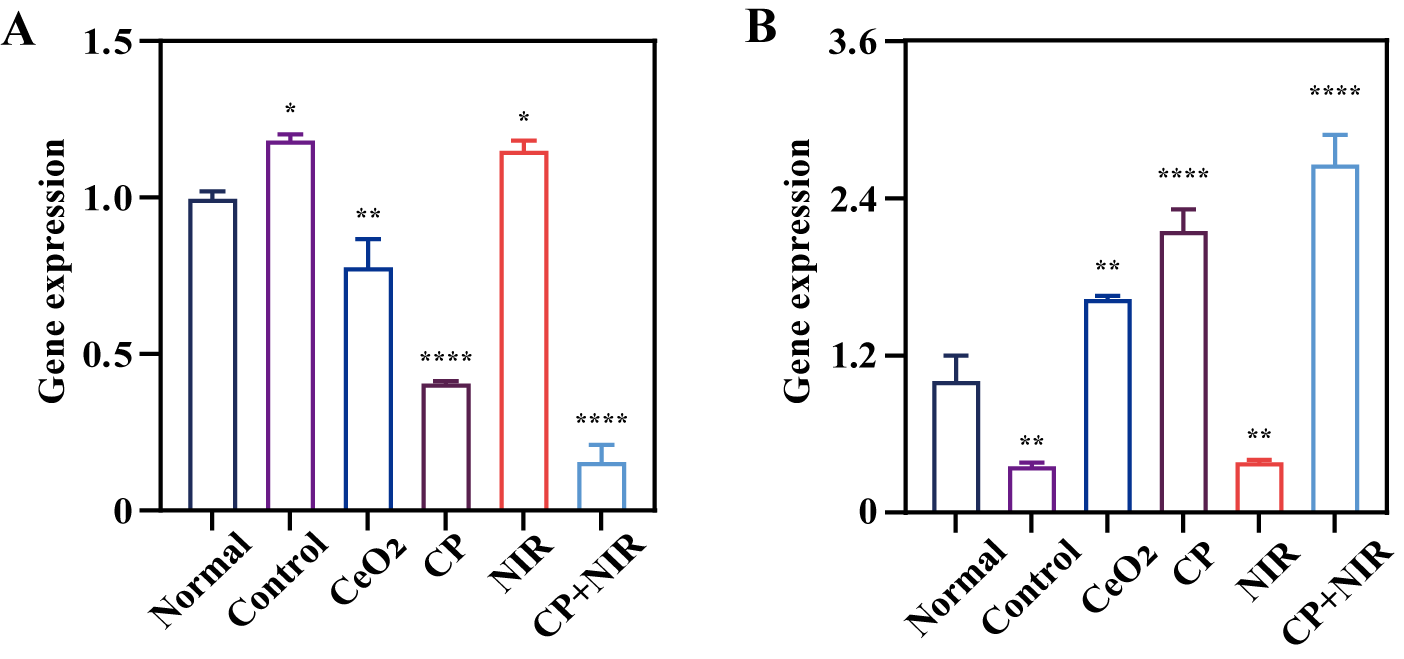


**Figure S41.** Relative genes expression levels of treated cells by RT-qPCR: IRF-1 (A) and SOCS1 (B). The corresponding groups were: cells without treatment (normal group), LPS induced cells followed by PBS treatment (control group), LPS induced cells followed by 100 μg/mL CeO_2_ treatment (CeO_2_), LPS induced cells followed by 100 μg/mL CP treatment (CP), LPS induced cells followed by NIR irradiation (2 W/cm^2^) (NIR), and LPS induced cells followed by 100 μg/mL CP combining with NIR irradiation (2 W/cm^2^) (CP+NIR). (“*” symbol compared with normal group, *p < 0.05, **p < 0.01, ***p < 0.001 and ****p < 0.0001)


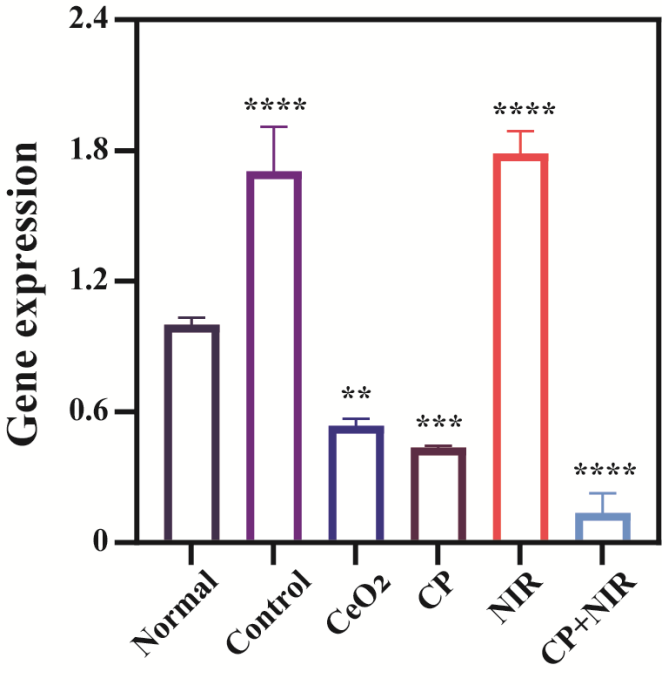


**Figure S42.** Relative gene expression levels of treated cells by RT-qPCR: NLRP3. The corresponding groups were: cells without treatment (normal group), LPS induced cells followed by PBS treatment (control group), LPS induced cells followed by 100 μg/mL CeO_2_ treatment (CeO_2_), LPS induced cells followed by 100 μg/mL CP treatment (CP), LPS induced cells followed by NIR irradiation (2 W/cm^2^) (NIR), and LPS induced cells followed by 100 μg/mL CP combining with NIR irradiation (2 W/cm^2^) (CP+NIR). (“*” symbol compared with normal group, *p < 0.05, **p < 0.01, ***p < 0.001 and ****p < 0.0001)

**
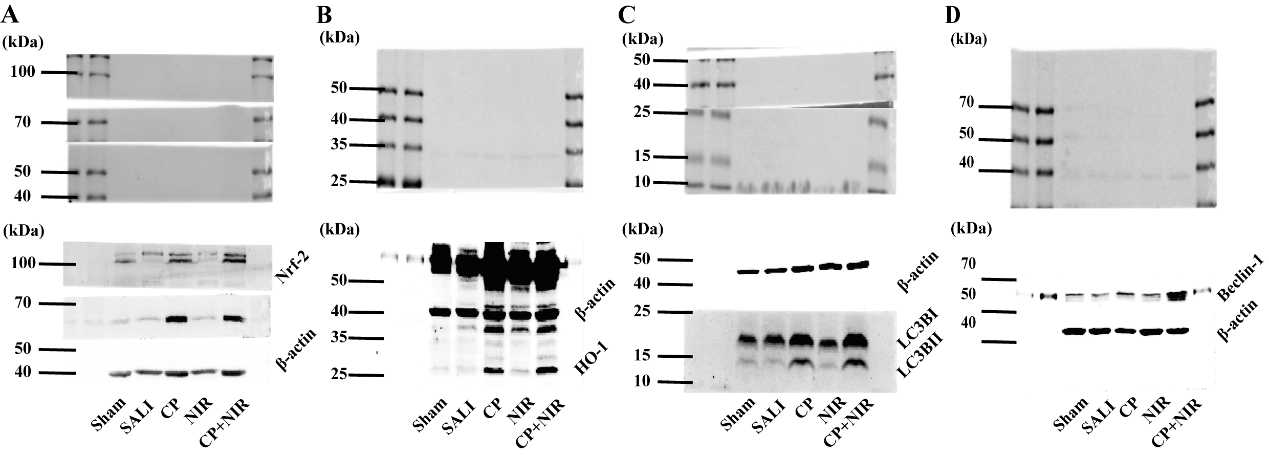
**

**Figure S43.** Original images of relative proteins expression levels of liver tissue of treated mice by WB: Nrf-2 (A), HO-1 (B), LCB3 I and LCB3 II (C) and Beclin-1 (D). The corresponding groups were: mice without treatment (sham group), LPS induced mice with saline injection (SALI), LPS induced mice with CP injection (CP), LPS induced mice with NIR irradiation (2 W/cm^2^) (NIR), and LPS induced mice with CP injection and NIR irradiation (2 W/cm^2^) (CP+NIR).
