# Supplementary material for: NIR Driven Pd/Cerium Oxide Nano‐Heterojunction for Enhanced Salvaging Sepsis Induced Acute Liver Injury via Reprogramming Redox Homeostasis in Synergy with Inducing Autophagy
Source: Adv Sci (Weinh). 2025 Jun 29;12(32):e17252. doi: 10.1002/advs.202417252 (PMC12407304; doi:10.1002/advs.202417252)
Supplement: Supplementary file 2 — Supporting Information [file ADVS-12-e17252-s001.docx]

Table S1. Element composition of CP by XPS and ICP-MS.

| Name | XPS | | | | ICP-MS | |
| --- | --- | --- | --- | --- | --- | --- |
|  | C (%) | O (%) | Ce (%) | Pd (%) | Ce (%) | Pd (%) |
| CP | 15.99 | 25.96 | 43.13 | 14.92 | 8.46 ± 0.91 | 8.02 ± 0.83 |

Table S2. ROS scavenging ratio of CeO_2_, CP and CP+NIR with the concentration of 100 μg/mL.

| Name | CeO_2_ (%) | CP (%) | CP +NIR (%) |
| --- | --- | --- | --- |
| H_2_O_2_ | 31.00 ± 0.77 | 66.02 ± 0.38 | 83.78 ± 0.17 |
| ·OH | 21.77 ± 0.43 | 38.75 ± 0.57 | 67.66 ± 1.42 |
| ·O_2_^-^ | 63.94 ± 0.53 | 92.10 ± 0.28 | 96.50 ± 0.20 |

Table S3. ROS scavenging ratio of CP with different concentrations.

| Name | 50 μg/mL (%) | 100 μg/mL (%) | 200 μg/mL (%) |
| --- | --- | --- | --- |
| H_2_O_2_ | 50.61 ± 0.65 | 66.02 ± 0.38 | 67.71 ± 0.77 |
| ·OH | 24.04 ± 1.35 | 38.75 ± 0.57 | 63.72 ± 1.70 |
| ·O_2_^-^ | 63.98 ± 0.44 | 92.10 ± 0.28 | 94.57 ± 0.10 |

Table S4. ROS scavenging ratio of 100 μg/mL CP, NIR and 100 μg/mL CP+NIR.

| Name | CP (%) | NIR (%) | CP+NIR (%) |
| --- | --- | --- | --- |
| ·OH | 44.40 ± 0.90 | 3.21 ± 0.21 | 76.79 ± 1.15 |
| ·O_2_^-^ | 73.91 ± 0.05 | 8.67 ± 5.22 | 82.61 ± 3.01 |

Table S5. Blood indicators of treated mice. The corresponding groups were: mice without treatment (Sham) and with CP+NIR injection (CP+NIR) after 7 days.

| Name | Sham | CP+NIR |
| --- | --- | --- |
| ALT (U/L) | 46.86 ± 7.86 | 52.95 ± 10.48 |
| AST (U/L) | 136.00 ± 15.27 | 158.10 ± 82.82 |
| CREA (μmol/L) | 24.94 ± 7.40 | 13.27 ± 4.79 |
| CK (U/L) | 562.30 ± 85.19 | 776.10 ± 495.00 |
| TBIL (μmol/L) | 6.74 ± 3.86 | 6.91 ± 4.98 |
| BUN (mg/dL) | 22.88 ± 1.20 | 17.57 ± 10.45 |

| Gene | Forward sequences (5’ to 3’) | Reverse sequences (3’ to 5’) |
| --- | --- | --- |
| ACTB | TGAGAGGGAAATCGTGCGTGAC | GGAAGAGGATGCGGCAGTGG |
| TNF-α | CACGCTCTTCTGTCTACTGAACTTC | CTTGGTGGTTTGTGAGTGTGAGG |
| IL-1β | CTCGCAGCAGCAC | CCACGGGAAAGA |
| iNOS | TCACTCAGCCAAGCCCTCAC | TCCAATCTCTGCCTATCCGTCTC |
| CD68 | CTCTTGCTGCCTCT | GCTGGTAGGTTGA |
| CD206 | TCTGGTGAACGGAATGATTGTGTAG | GCTTTGGTTGTAATGGATGAGTGTG |
| HSP70 | TGGTGCTGACGAAGATGAAGGA | TGCCGCTGAGAGTCGTTGAAG |
| HO-1 | AGAAGAGGCTAAGACCGCCT | ACGCCATCTGTGAGGGACTC |
| NLRP3 | TCACAACTCGCCCAAGGAGGAA | AAGAGACCACGGCAGAAGCTAG |
| IRF-1 | TCCAAGTCCAGCCGAGACACTA | ACTGCTGTGGTCATCAGGTAGG |
| SOCS1 | AGTCGCCAACGGAACTGCTTCT | GTAGTGCTCCAGCAGCTCGAAA |
| Keap1 | ATCCAGAGAGGAATGAGTGGCG | TCAACTGGTCCTGCCCATCGTA |
| GCLM | CTAGACAAGACACAGTTGGAGCAG | AGAGCAGTTCTTTCGGGTCATTG |
| p65 | CACCTTATGGAGACCAAGCC | TGTGGAGATCATCGGCTCAG |
| COX-2 | TGAGTACCGCAAACGCTTCTC | TGGACGAGGTTTTTCCACCAG |
| NQO1 | ATGAAGGAGGCTGCTGTAGAGG | TGCTAGAGATGACTCGGAAGGATAC |

Table S6. Detailed primer sequences for qRT-PCR.
